# Developing and evaluating rare disease educational materials co-created by expert clinicians and patients: the paradigm of congenital hypogonadotropic hypogonadism

**DOI:** 10.1186/s13023-017-0608-2

**Published:** 2017-03-20

**Authors:** COST Action BM1105, Corin Badiu, Marco Bonomi, Ivan Borshchevsky, Martine Cools, Margarita Craen, Cristina Ghervan, Michael Hauschild, Eli Hershkovitz, Erik Hrabovszky, Anders Juul, Soo-Hyun Kim, Phillip Kumanov, Beatriz Lecumberri, Manuel C. Lemos, Vassos Neocleous, Marek Niedziela, Sandra Pekic Djurdjevic, Luca Persani, Franziska Phan-Hug, Duarte Pignatelli, Nelly Pitteloud, Vera Popovic, Richard Quinton, Nicos Skordis, Neil Smith, Magdalena Avbelj Stefanija, Cheng Xu, Jacques Young, Andrew A. Dwyer

**Affiliations:** 1 Department of Endocrinology, National Institute of Endocrinology, C. Davila University of Medicine and Pharmacy, Bucharest, 050474 Romania; 2 Deptartment of Clinical Sciences & Community Health and the Division of Endocrine and Metabolic Diseases & Laboratory of Endocrine and Metabolic Research, University of Milan, Milan, Italy; 3 Ospedale San Luca, IRCCS Istituto Auxologico Italiano, Piazzale Brescia 20, 20149 Milan, Italy; 4 Patient Advocacy Working Group and the International Medical Interpreters Association, Pyatigorsk, Russia; 5 University Hospital Ghent Department of Pediatric Endocrinology, Ghent University, De Pintelaan 185, 9000 Ghent, Belgium; 6 University of Medicine and Pharmacy “Iuliu Hatieganu”, 8, V Babes str., 400012 Cluj-Napoca, Romania; 7 Endocrinology, Diabetes & Obesity Service of the Department of Pediatric Medicine and Surgery, Children’s Hospital of Lausanne, Chemin de Montétan 16, 1004 Lausanne, Switzerland; 8 Pediatric Endocrinology and Metabolism Unit, Faculty of Health Sciences, Soroka Medical Center, Ben-Gurion University of the Negev, PO Box 151, IL-84101 Beer-Sheva, Israel; 9 Laboratory of Endocrine Neurobiology of the Institute of Experimental Medicine of the Hungarian Academy of Sciences, 43 Szigony St., 1083 Budapest, Hungary; 10 Department of Growth and Reproduction GR, Rigshospitalet section 5064, Blegdamsvej 9, 2100 Copenhagen, Denmark; 11 St. George’s Medical School of the University of London, Molecular & Clinical Sciences Research Institute, Cell Biology & Genetics Research Centre, Cranmer Terrace, London, SW17 0RE UK; 12 Clinical Center of Endocrinology, Medical University, 2 Zdrawe St, 1431 Sofia, Bulgaria; 13 Autónoma University of Madrid, Hospital La Paz Institute of Health Research (IdiPAZ), Endocrinology and Nutrition Service of La Paz University Hospital, Castellana 261, 28046 Madrid, Spain; 14 CICS-UBI, Health Sciences Research Centre, University of Beira Interior, Covilha, 6200-506 Portugal; 15 Department of Molecular Genetics of the Cyprus Institute of Neurology and Genetics, P.O. Box 23462, Nicosia, Cyprus; 16 Department of Pediatric Endocrinology and Rheumatology, Poznan University of Medical Sciences, Szpitalna Street 27/33, 60-572 Poznan, Poland; 17 School of Medicine, University of Belgrade & Clinic of Endocrinology, Diabetes and Metabolic Diseases, University Clinical Center Belgrade, dr Subotic 13, 11000 Belgrade, Serbia; 18 Department of Clinical Sciences & Community Health, University of Milan, Milan, Italy; 19 Division of Endocrine and Metabolic Diseases & Laboratory of Endocrine and Metabolic Research, Milan, Italy; 20 Department of Endocrinology, Hospital S João, Alameda Hernani Monteiro, 4200 Porto, Portugal; 21 University of Lausanne and the Endocrinology, Diabetes & Metabolism Service, Lausanne University Hospital, Rue du Bugnon 46, Lausanne, 1011 Switzerland; 22 School of Medicine, University of Belgrade, dr Subotic 8, 11000 Belgrade, Serbia; 23 University of Newcastle-upon-Tyne, Institute of Genetic Medicine and the Royal Victoria Infirmary, Newcastle-upon-Tyne, NE1 3BZ UK; 24 Division of Pediatric Endocrinology, Paedi Center for specialized Pediatrics, Nicosia, Cyprus; 25 St George’s University of London Medical School at the University of Nicosia, Nicosia, Cyprus; 26 Patient Advocacy Working Group, London, UK; 27 University Medical Centre Ljubljana, University Children’s Hospital, Dept. of pediatric endocrinology, diabetes and metabolism, Bohoriceva ul. 20, 1000 Ljubljana, Slovenia; 28 Service d’Endocrinologie et des Maladies de la Reproduction, Hôpital Bicêtre, Le Kremlin-Bicêtre, 94275 France; 29 University of Lausanne, Institute of Higher Education & Research in Healthcare and the Endocrinology, Diabetes & Metabolism Service of the Lausanne University Hospital, Route de la Corniche 10, Lausanne, 1010 Switzerland

**Keywords:** Congenital hypogonadotropic hypogonadism, Kallmann syndrome, Rare diseases, E-health, Community based participatory research, Patient education, Patient participation, Patient-centered care, Nursing

## Abstract

**Background:**

Patients with rare diseases face health disparities and are often challenged to find accurate information about their condition. We aimed to use the best available evidence and community partnerships to produce patient education materials for congenital hypogonadotropic hypogonadism (CHH) and the olfacto-genital (Kallmann) syndrome (i.e., CHH and defective sense of smell), and to evaluate end-user acceptability. Expert clinicians, researchers and patients co-created the materials in a multi-step process. Six validated algorithms were used to assess reading level of the final product. Comprehensibility and actionability were measured using the Patient Education Materials Assessment Tool via web-based data collection. Descriptive statistics were employed to summarize data and thematic analysis for analyzing open-ended responses. Subsequently, translation and cultural adaption were conducted by clinicians and patients who are native speakers.

**Results:**

Co-created patient education materials reached the target 6^th^ grade reading level according to 2/6 (33%) algorithms (range: grade 5.9–9.7). The online survey received 164 hits in 2 months and 63/159 (40%) of eligible patients completed the evaluation. Patients ranged in age from 18 to 66 years (median 36, mean 39 ± 11) and 52/63 (83%), had adequate health literacy. Patients scored understandability at 94.2% and actionability at 90.5%. The patient education materials were culturally adapted and translated into 20 languages (available in Additional file 1).

**Conclusions:**

Partnering with patients enabled us to create patient education materials that met patient- identified needs as evidenced by high end-user acceptability, understandability and actionability. The web-based evaluation was effective for reaching dispersed rare disease patients. Combining dissemination via traditional healthcare professional platforms as well as patient-centric sites can facilitate broad uptake of culturally adapted translations. This process may serve as a roadmap for creating patient education materials for other rare diseases.

**Electronic supplementary material:**

The online version of this article (doi:10.1186/s13023-017-0608-2) contains supplementary material, which is available to authorized users.

## Background

The landmark 2009 report from the European Organization for Rare Diseases (EURORDIS) brought to light the many challenges faced by patients with rare diseases [1]. Delays in diagnosis, difficulty finding information about their condition and inadequate access to expert care are frequent patient experiences. Indeed, some have posited that living with a rare disease places one in the realm of health disparities [2]. Physical and psychological morbidity can be significant and feelings of isolation and powerlessness can further undermine quality of life [3]. Importantly, potential means to overcome these challenges include using the internet to connect dispersed patients with expert care and community engagement to help empower patients who feel marginalized by the healthcare system [4–6].

One such rare disorder is congenital hypogonadotropic hypogonadism (CHH, ORPHA174590). Based on a study of French conscripts, CHH occurs in approximately one in 4,000-10,000 [7]. It is clinically characterized by incomplete (or absent) puberty and infertility resulting from insufficient secretion or action of gonadotropin releasing hormone (GnRH) - the master hormone of the reproductive axis [8]. Genetic defects that affect olfactory axon and olfactory bulb development, thus leading to absent or defective sense of smell (anosmia or hyposmia), usually also affect the migration of neuroendocrine GnRH cells from the nose to the brain during embryonic life, thus leading to CHH. This condition is known as the olfacto-genital syndrome or Kallmann syndrome (ORPHA478). Some genetic and phenotypic overlap may exist between isolated CHH and Kallmann syndrome, yet patients with this syndrome are much more likely to exhibit additional non-reproductive phenotypes (i.e., skeletal defects, renal agenesis, cleft lip/palate, deafness, etc) than normosmic CHH patients. In the context of an international network of leading clinicians/geneticists/researchers focused on CHH [9], we have previously developed patient partnerships and conducted a needs assessment that leveraged engagement with patient support groups, social media and online data collection [10]. In parallel, we developed a web-based platform [9] with resources for patients to find expert clinicians and peer-to-peer support. Additionally, consensus guidelines for the diagnosis and treatment of CHH were created using an evidence-based approach [8].

Engagement and co-creation have been effectively used in diverse fields including business, design and computer science (i.e. user-centered design) as a means to spur innovation, adoption and foster sustainability [11, 12]. Therefore, the aim of the present study was to partner clinical experts and patients to co-create high-quality patient education materials (PEM) that respond to the issues and questions most important and relevant to patients. Secondary aims included evaluating the readability of the PEM and end-user acceptability (i.e. understandability and actionability) as well as to disseminate these materials widely across different countries and cultures.

## Methods

### PEM development

**Fig. 1:**
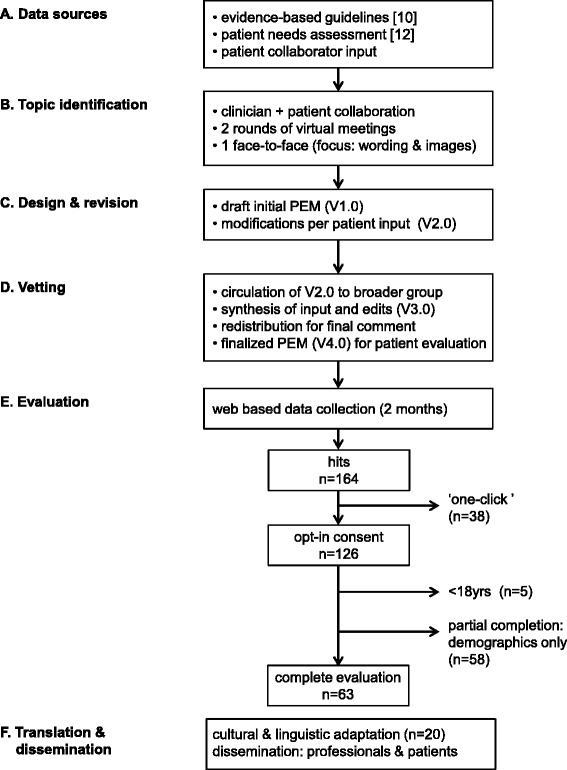
Study Schema. PEM were co-created in a multi-step process. (**a**) Three main sources were used for PEM development. (**b**) Members of the Patient Advocacy Working Group and patient collaborators identified topics for the PEM in an iterative process. (**c**) The initial draft was created and revised based on patient input. (**d**) PEM (V2.0) was circulated to the Clinical Working Group and Genetics Working Group members for comment and revised accordingly with patient validation in two rounds. (**e**) PEM (V4.0) were evaluated by patients recruited via social media (private/closed Facebook groups), patient support meetings and via RareConnect [12]. (**f**) Following evaluation materials were culturally adapted and translated to 20 languages and distributed in avenues targeting healthcare professionals and patients. PEM: patient education materials, V: version

A community based participatory research framework was selected to guide the development of the patient education material (PEM) for its relevance to patient empowerment and health disparities [13]. The Patient advocacy Working group of the European network focused on CHH (COST Action BM1105, “GnRH Deficiency: Elucidation of the neuroendocrine control of human reproduction”) [9] worked closely with online patient community leaders (i.e. moderators of online patient support sites) to identify key PEM content areas and topics based on the most frequently asked questions on social media sites (Additional file 1) as well as from a previously conducted patient needs assessment [10]. Clinical information was drawn from the evidence-based consensus statement on the approach to diagnosis and treatment of CHH [8]. The PEM development was an iterative process (Fig. 1) involving multiple stakeholders including patients, patient support groups, clinicians and researchers spanning the fields of endocrinology, andrology, nursing and genetics. At each step, input and feedback were used to refine and modify the PEM.

### Readability assessment

To assess reading level of the produced PEM, we subjected the final version to several validated measures evaluating readability: Flesch Reading Ease Formula (evaluates sentence length and number of syllables per word), Flesch Kincaid Grade Level (converts the Flesch reading ease formula to a grade level), Gunning Fox Index (calculates a weighted average of the number of words per sentence and long words to determine grade level), Coleman Liau Index (uses number of characters rather than syllables to determine grade level), Simple Measure of Gobbledygook (SMOG, a modification of the Gunning-Fog Index it calculates grade level based on the number of words with 3 or more syllables) and the Automated Readability Index (ratio of difficult words and sentences to provide an estimated age range and grade level) [14].

### End-user acceptability

To evaluate end-user perspectives of adults with CHH (18 years and older), we used an online data collection (SurveyGizmo™) and recruited a convenience sample of patients via postings on closed/private CHH social media group (Facebook™), as well as notifications in patient support group meetings and RareConnect [15]. This social media approach has been previously shown to be an effective means of recruitment for this rare disease patient population [10]. The survey included questions on patient demographics, past healthcare interactions and a brief assessment of healthcare literacy that has been validated against longer gold-standards metrics [16, 17].

After reviewing a pdf of the PEM, participants were asked to complete the Patient Education Materials Assessment Tool (PEMAT). This instrument was developed and validated by the U.S. Department of Health & Human Services Agency for Health Research & Quality to evaluate print and audiovisual educational materials [18]. The unique aspect of the PEMAT is that it incorporates other additional elements that are not assessed in traditional readability formulas. Patients select agree, disagree or not-applicable for 17 items relating to understandability (the ability to process key messages) and 7 items on actionabilty (the ability to identify what one can do to manage their condition). Items rated as agree are given a score of 1, disagree 0 and cumulative scores are expressed as a percentage (total score/possible total X 100). Initial psychometric evaluation of the PEMAT has demonstrated strong internal consistency, good reliability, and initial evidence of construct validity [19]. Survey respondents were also given an opportunity to provide free text comments (i.e. critiques and suggestions) after completing the PEMAT questions.

### Statistical analyses

The survey was alpha tested by patients in two rounds to identify and correct any bugs prior to online launch. Descriptive statistics were used to report summary findings. To assess for potential response bias, Student’s *t* test and Chi square test were used to compare demographic characteristics of patients who completed the evaluation with those who did not (partial completion). Thematic analysis [20] was employed to codify and analyze open-text responses NVivo11 (QSR International PSY Ltd., Melbourne Australia). The study was reviewed and approved by the ethics committee of the University of Lausanne and participants provided opt-in online consent.

### Dissemination

The final step of this process was to disseminate the PEM to reach the broadest possible audience. This included using native speakers (i.e. expert clinicians, medical translators) from across the European network to provide versions in multiple languages. Particular attention was given to finding appropriate terms and examples for the translated PEM to make them culturally sensitive and not simply verbatim translations. The final materials will be distributed via traditional means to reach healthcare professionals (i.e. peer-review publication, professional meetings, individual providers’ websites, and via the COST Action website [9]). In parallel PEMs will be distributed via patient support groups including online social media (Facebook™, Twitter™, patient blogs) and publicized on internet platforms targeting the rare disease community including the EURORDIS initiative RareConnect [15].

## Results

### PEM development

Patient partnerships were used to identify key topics and to target issues most important to patients as well as to contribute content. A working group of the network (Patient Advocacy Working Group) created a topic list based on the consensus statement guidelines [8]. Additional items were drawn from focus group discussions with patients held in the context of patient support meetings (organized with patient leaders) as part of the prior needs assessment [10] (Fig. 1A). Patient collaborators also contributed lists of “frequently asked questions” as well as topics that were recurrent in social media threads and chat room discussions. Common questions include: what causes CHH? Why didn’t I go through puberty? Why can’t I smell? Is it curable? Can I have children? Will my children have CHH? (see Additional file 1). The drafted material version 1.0 (V1.0) went through two subsequent revisions to refine language, wording and selection of images via email and the PEM was finalized in a face-to-face meeting prior to vetting with the broader network (Fig. 1D). During this development process it was sometimes challenging to balance the input and feedback from clinicians and patients to find the right balance and depth of information provided. Indeed, the face-to-face meeting was valuable for arriving at consensus as opinions were conflicting at times during the process and this was not easy to reconcile via email.

### Readability levels

Readability was evaluated using 6 different validated algorithms that are widely used to assess reading level (Table 1). These employ different formulas that use word length and complexity (i.e. the number of characters or syllables in words, sentence length) to calculate an average grade level needed to understand the material. Most patients read at an 8-9^th^ grade reading level [21]. However, expert recommendation has identified the target reading level at 6^th^ grade (i.e. an 11 year-old child) [22]. Two of the six algorithms scored the PEM at the target grade level (Flesch Kincaid Grade Level: 5.9, Automated Readability Index: 6.1). The mean grade level across instruments was 8.0 (Table 1), indicating that more work could be done to enhance readability. However, one challenge in doing this is the number of complex words (i.e. hypogonadism, cryptorchidism, infertility, etc) that were deemed important by clinicians and patients alike to include and define in lay terms.

**Table 1:** Summary of readability of co-created patient education material

Summary statistics (5 pages, 203 sentences + images)		
Word count = 1,654		
Character count = 8,251		
Complex words (≥3 syllables) = 268		
Average characters per word = 5		
Average words per sentence = 8.1		
Instrument	Score	Grade level
Flesch Reading Ease	67.6	8-9^th^
Flesch-Kincaid Reading Level	5.9	6^th^
Gunning Fog Index	9.7	9-10^th^
Coleman Liau Index	8.8	8-9^th^
SMOG	9.7	9^th^
Automated Readability Index	6.1	6^th^
Mean	8.0	8^th^ grade

### Participants

Following patient alpha testing to identify and correct bugs in the online evaluation, the survey was launched and remained open for 8-weeks. During this period, 164 hits were registered. In total, 38 (23%) were “one-click” entries who passed the opt-in consent but did not enter demographic information. Responses of five participants were excluded (age <18 years). More than a third of respondents (58/164, 35%) partially completed the evaluation (i.e. demographics up to viewing the PEM) and 63 (38%) completed the entire PEM evaluation (Fig. 1E). Characteristics of survey respondents are depicted in Table 2. Notably, the predominance of male responders (2:1) is keeping with the striking sexual discordance in CHH [8]. Overall, patients were well-educated (46/63, 73% achieving university or higher) and by-and-large exhibited adequate health literacy (52/63, 82%). Notably, the mean age of diagnosis was 20.9 ± 6.4 years (range: 10–40, median 19) suggesting that many patients are diagnosed quite late. In terms of prior healthcare interactions, more than half (39/63, 62%) had either a consultation or had received care at a specialized academic center. In total, 36/63 (56%) had undergone genetic testing yet only 12/63 (19%) reported having had genetic counseling. We found no significant differences between those who completed the evaluation and the partial completers in terms of age (*p* = 0.30), sex (*p* = 0.37), education (*p* = 0.94), health literacy (*p* = 0.15), or being seen at an academic center (*p* = 0.09).

**Table 2:** Patient characteristics (*n* = 63)

Sex	n (%)
Male	42 (67%)
Female	21 (33%)
Age	
18–29	13 (21%)
30–39	24 (38%)
40–49	17 (27%)
50–59	5 (8%)
60+	4 (6%)
Children	
None	42 (66%)
Biologic children	14 (22%)
Adopted children	7 (11%)
Education level	
High school/vocational	17 (27%)
University	25 (40%)
Post-Graduate	21 (33%)
Health literacy^a^	
Adequate	52 (83%)
Inadequate	11 (17%)
Health experiences	
Seen at academic center	39 (62%)
Had genetic testing	36 (57%)
Received genetic counseling	12 (19%)

^a^health literacy as assessed by [16, 17]

### End-user acceptability

Patients gave the co-created PEM high scores on understandability (range: 88.9–97.5%, total mean: 94.2%) which includes content, word choice/style, use of numbers, organization, layout/design and visual aids (Table 3). The lowest rating (88.9%) was linked with being uncluttered which was commented on in the free text field by three patients (i.e. having more white space). Similarly, patients gave high scores on actionability (overall mean: 90.5%). The lowest score was assigned to explaining how to use charts, graphs, or diagrams to take action and manage the condition. Together the high scores on both understandability and actionability indicate high end-user acceptability.

**Table 3:** PEMAT Understandability and actionability of co-created materials (*n* = 63)

PEMAT topic/category	% agree
Content	92.1%
The material makes its purpose completely evident.	
The material does not include information or content that distracts from its purpose.	90.5%
Word choice & style	93.7%
The material uses common, everyday language.	
Medical terms are used only to familiarize the audience with the terms. When used, medical terms are defined.	93.7%
The material uses the active voice (e.g. action verbs).	95.2%
Use of numbers	96.8%
Numbers appearing in the material are clear and easy to understand.	
The material does not expect the user to perform calculations.	96.8%
Organization	93.7%
The material breaks or “chunks” information into short sections.	
The material’s sections have informative headers.	95.2%
The material presents information in a logical sequence.	93.7%
The material provides a summary.	96.8%
Layout & design	95.2%
The material uses visual cues to draw attention to key points.	
Visual aids	95.2%
The material uses visual aids whenever they could make content more easily understood.	
The material’s visual aids reinforce rather than distract from the content.	92.1%
The material’s visual aids have clear titles or captions.	95.2%
The material uses illustrations and photographs that are clear and uncluttered.	88.9%
The material uses simple tables with short and clear row and column headings.	97.5%
Total understandability	94.2%
Actionability	95.2%
The material clearly identifies at least one action the user can take.	
The material addresses the user directly when describing actions.	92.1%
The material breaks down any action into manageable, explicit steps.	92.5%
The material provides a tangible tool (e.g. checklists) whenever it could help the user take action.	94.5%
The material explains how to use the charts, graphs, tables, or diagrams to take actions	70.7%
The material uses visual aids whenever they could make it easier to act on the instructions.	92.0%
Total actionability	90.5%

Overall we received comments from 45/63 (71.4%) patients. Comments were coded according to themes and sorted into categories. In total, 52 concepts were identified from the 45 comments clustering into five categories (Table 4). The most frequent sentiments were expressions of thanks/approval (*n* = 19, 37%) followed by content (i.e. treatment, infertility, and psychological aspects) *n* = 11 (21%), format (i.e. use of simple language, spacing) *n* = 10 (19%), personal concerns (*n* = 9, 17%) and three comments underscored the importance of translating the PEM to make it available to more patients.

**Table 4:** Patient comments (*n* = 52) regarding the co-created materials

Category	Representative quote(s)
Thanks/approval *n* = 19	• “I was very impressed and I think my friends and family will find it easy to understand” • “I am glad that there is a clear male/female explanation. Often materials I find focus predominantly on the males”
Content *n* = 11	• “Elaborate on infertility and treatment” • “I think you may be under discussing the life-long emotional and psychological impact”
Formatting *n* = 10	• “It felt like the pages were a bit full but I can appreciate it must have been hard to provide all the information necessary in only 5 pages” • “I think if there was a way to click on each section for more detailed info that might help”
Personal concerns *n* = 9	• “Other rare conditions that can also be evident in patients with KS/CHH… …Explain that patients can have additional illnesses besides KS/CHH” • “It might be helpful to state that anosmia is permanent. Of course it is most important to focus on the hormonal component, but there are definitely considerations in dealing with anosmia as well”
Dissemination *n* = 3	• “It would be great if you could translate into several languages to reach more people”

### Broad dissemination

Native speakers from across the European network made culturally adapted translations. In some instances local patients contributed to this translation and adaption process. The translated PEMs required cultural adaptation in some instances to help make them more relevant for the target audience. For instance, a small cherry was used to describe the size of the pituitary gland in the Hungarian version, the Chinese version was altered as “what you should know” was not culturally appropriate, and terms describing depression were adapted in the Polish version to enhance comprehension by the lay public. Every effort was made to keep the entire content of the PEM in the translated versions. When text length expanded the images were adjusted accordingly to maintain a 5-page document. PEM are now available in 20 languages: English, Bulgarian, Chinese, Danish, Dutch, French, German, Greek, Hebrew, Hungarian, Italian, Korean, Polish, Portuguese, Romanian, Russian, Serbian, Slovenian, Spanish, and Turkish (Additional file 2). Dissemination plans will target healthcare professionals and patient-centric avenues such as social media and patient support sites.

## Discussion

The aim of this study was to engage patients and co-create PEM that respond to what matters most to patients. Subsequently, we evaluated the readability and end-user acceptability of the PEM and sought to widely disseminate the translated PEM across different countries and cultures. Patients living with a rare disease face health disparities [2] and patient engagement has been identified as potential means to empower this patient population [4–6]. Interestingly, patient engagement has recently been gaining attention in the context of orphan drug development [23]. However, the extent of patient engagement varies widely. A 2014 systematic review of patient engagement for research on rare diseases found engagement is typically unidirectional - involving patients in consultative roles and rarely in creative aspects or in terms of dissemination [24]. The present study is unique in that we used a participatory process to co-create PEM with patients; we then evaluated the PEM produced by this collaboration, and worked with patient groups to facilitate dissemination to the largest possible audience.

We previously partnered with online patient community leaders to identify the unmet health and informational needs of patients with congenital hypogonadotropic hypogonadism (CHH) and Kallmann syndrome [10]. In the present study, the partnership was more clearly bi-directional as patients were not simply providers of opinions; rather they contributed directly in co-creating the PEM in an iterative process. Notably, patient knowledge and expertise emerges from the day-to-day experiences of living and coping with a rare condition and therefore is inherently different from the expertise of healthcare professionals [25]. Recently, a study examining online exchanges among patients with rare adrenal disorders found that information and support were central elements in peer-to-peer exchanges [26]. Moreover, the authors noted that patient-centered care could be enhanced by better integrating patient knowledge with the care provided by professionals. In the present study, developing the PEM was a true partnership that recognized patient expertise as unique and complementary to expert clinician knowledge. We believe that this co-creation contributed to the high acceptability ratings by patients.

This evaluation process of the co-created PEM has limitations. The evaluation was only conducted on the English version. As such, the findings are not completely transferable to the other translated versions despite the inclusion of patients in developing some of the translations. Moreover, the additional validation step of back translating the other versions was not conducted and this could be viewed as a limitation. We only assessed readability once the materials had been finalized, not during the development process. In future studies, this testing could be incorporated earlier in the development process to improve the reading level of developed PEM. While the evaluation was overwhelmingly positive and a fairly sizeable sample was reached (for a rare disease population), the patients completing the evaluation were quite well-educated and exhibited high levels of health literacy. Accordingly, our ability to draw inferences to a broader population of lower literacy patients is limited. This may reflect a bias of using a web-based survey - as perhaps those using the web may have higher literacy levels. However, recruiting sufficient numbers of patients for rare disease studies has been a long-standing challenge [27, 28]. Therefore, we used a web-based approach to overcome this barrier but note that such an approach entails a potential risk of bias.

The Pew Foundation’s published report on health and the internet indicates that patients living with a rare disease are internet power users who are most likely to seek information about their condition online and find support from other patients using social media [29]. Based on our previous success combining patient partnerships and social media for the online needs assessment [10], we employed a similar approach in the present study to reach a relatively large sample (*n* = 63) over 8-weeks. These experiences suggest that web-based platforms are an effective means to reach and connect rare disease patients. Thus, the opportunities afforded by the internet and social media may provide novel avenues for crowdsourcing solutions as well as offering a shared venue for either clinician- or patient-led collaborations to improve quality and add value to the healthcare system [5, 30]. The European Union Committee of Experts on Rare Diseases (EUCERD) recommendations for Centers of Expertise underscore the importance of collaboration with patient organizations to provide information that is at once accessible and adapted to patient needs [31]. For many rare diseases, such as CHH/Kallmann syndrome, formal organized patient support organizations do not exist. As such, web-based approaches and social media provide a critical means to broadly reach patients, identify priorities and incorporate their perspectives and knowledge into care. This may be particularly advantageous in light of the movement to form European Reference Networks for rare diseases [32, 33].

The final step in this co-creation process was to engage in bi-directional dissemination. This has been identified as a shortcoming in much of the patient engagement research conducted in the context of rare diseases [24]. Through the work of members of the Network and patients alike, materials were adapted and translated into 20 languages by native speakers. This collaborative process is essential for ensuring that information provided to patients is culturally adapted and sensitive – a key element for Centers of Expertise [31]. In parallel to traditional healthcare professional outlets (e.g. scientific meetings, peer-review publication) patient participants are distributing materials directly to other patients via social media and postings on centralized patients sites [15]. The co-created PEM (in multiple languages) is a critical component of the list of patient resources available on the website of the European network comprising a virtual empowerment toolkit for patients and families [9]. Available information includes listings of international specialized referral centers, genetic testing labs, clinical trials, and peer-to-peer support as well as a portal for a patient registry. We are utilizing both professional-oriented avenues and more patient-oriented social media outlets to hopefully reach unprecedented numbers of patients and clinicians and overcome traditional roadblocks of implementation into practice [34–36].

## Conclusions

Partnering with patients enabled co-creation of high-quality PEM while social media and web-based data collection facilitated timely evaluation by a dispersed patient population. We believe that partnering with expert patients was an empowering experience and provides valuable contributions for developing patient-centered approaches to care. We envision this work will serve as a roadmap for those wishing to engage in a co-creation process and will help inform projects aimed at improving care for patients living with a rare disease.

## Additional files

Additional file 1: Questions frequently asked by patients on the social media patient support site. (PDF 51 kb)
Additional file 2: Patient Education Materials. (PDF 3856 kb)

## Abbreviations

CHH: Congenital hypogonadotropic hypogonadism
EUCERD: European Union Committee of Experts on Rare Diseases
EURORDIS: European Organization for Rare Diseases
COST: European Cooperation in Science and Technology
PEM: Patient education materials
PEMAT: Patient education materials assessment tool
SMOG: Simple measure of Gobbledygook
