# Supplementary material for: Developing and evaluating rare disease educational materials co-created by expert clinicians and patients: the paradigm of congenital hypogonadotropic hypogonadism
Source: Orphanet J Rare Dis. 2017 Mar 20;12:57. doi: 10.1186/s13023-017-0608-2 (PMC5359990; doi:10.1186/s13023-017-0608-2)
Supplement: Additional file 1: — Questions frequently asked by patients on the social media patient support site. (PDF 51 kb) [file 13023_2017_608_MOESM1_ESM.pdf]

Supplemental Material 2.
Questions frequently asked by patients on the social media patient support site

What is Kallmann Syndrome?
What is congenital hypogonadotrophic hypogonadism?
What does the term CHH mean?
How are Kallmann syndrome and CHH different?
What does congenital mean?
What does hypogonadotrophic mean?
What is hypogonadism?
What is the normal function of the gonadal organs?
What goes wrong in CHH and Kallmann syndrome?
Is there anything wrong with the testes or ovaries?
What causes Kallmann syndrome or HH?
What is a hormone?
What is GnRH? Why is it important?
What does gonadotrophin mean?
What are GnRH conditions?
What happens in GnRH deficient conditions?
When does puberty normally start?
What normally happens in puberty?
Why did I not go through puberty?
Will I go through puberty?
Why do I appear to be so tall?
Why can’t I smell?
What is anosmia?
How would anosmia affect a person?
How did I get CHH or Kallmann syndrome?
Can I pass on CHH or Kallmann syndrome to another person?
Does CHH and Kallmann syndrome affect both men and women?
How many people are affected by CHH and Kallmann syndrome?
How would the absence of puberty affect somebody?
Does CHH or Kallmann syndrome have any effect on expected lifespan?
How is CHH or Kallmann syndrome diagnosed?
Can CHH or Kallmann syndrome be cured?
What types of treatment are available?
Are there any risks if KS or HH is left untreated?
Will I be able to have children?
Will I pass on CHH or Kallmann syndrome to any children I might have?
What is an endocrinologist?
