# Supplementary material for: Developing and evaluating rare disease educational materials co-created by expert clinicians and patients: the paradigm of congenital hypogonadotropic hypogonadism
Source: Orphanet J Rare Dis. 2017 Mar 20;12:57. doi: 10.1186/s13023-017-0608-2 (PMC5359990; doi:10.1186/s13023-017-0608-2)
Supplement: Additional file 2: — Patient Education Materials. (PDF 3856 kb) [file 13023_2017_608_MOESM2_ESM.pdf]

What you should know about congenital hypogonadotropic hypogonadism (CHH) & Kallmann syndrome (KS)

What is CHH?

CHH is caused by a **deficiency of GnRH (gonadotropin releasing hormone)**. When combined with a lack of sense of smell (anosmia), it is known as olfacto-genital syndrome or Kallmann syndrome (KS). All these are diagnosed and treated the same way.

What does GnRH do?

GnRH is an essential hormone for sexual development, puberty and fertility.

1. GnRH is released from the the hypothalamus, an area of the brain that controls several body functions.
2. GnRH acts directly on the pituitary gland (a nearby pea-sized gland in the brain) to release two hormones: luteinising hormone (LH) & follicle stimulating hormone (FSH).
3. LH & FSH are important for puberty and fertility
   - in men: LH & FSH stimulate the testes to make testosterone and sperm
   - in women: LH & FSH stimulate the ovaries to make oestrogen and progesterone that are important for fertility

What happens if there is no GnRH?

Complete or partial GnRH deficiency results in absent or very low LH and FSH. This results in **failure of puberty and infertility** (see figure below). This means that:

- in men: 1) the testes do not grow, 2) testosterone is not produced in normal amounts and 3) sperm does not develop.
- in women: 1) although eggs are present, they do not develop or grow and are not released (ovulation), 2) oestrogen and progesterone are not produced, and 3) no menstrual periods (bleeding) occur.

Signs of failed puberty in adolescents and young adults

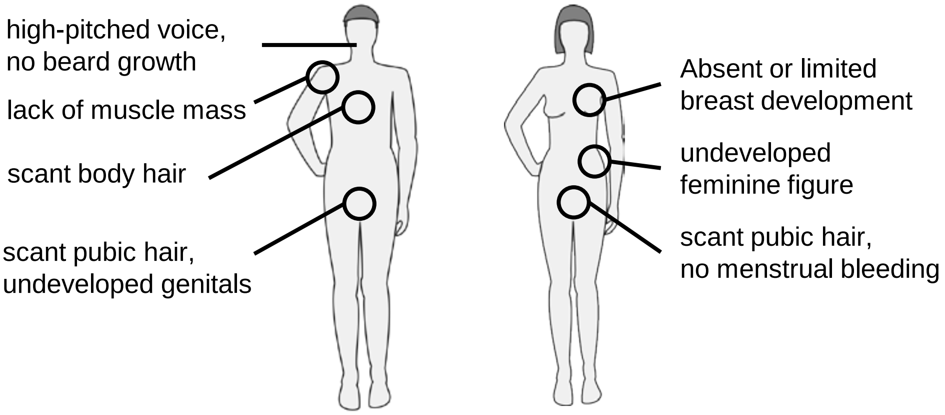

These problems are almost always present at birth (called congenital). However, the diagnosis is often made in teenage years or early adulthood when puberty does not start. **Importantly, this condition is treatable. In most cases it requires life-long hormone replacement & ongoing care.**

2016: www.gnrhnetwork.eu / andrew.dwyer@chuv.ch

Who is affected by CHH and how is it diagnosed?

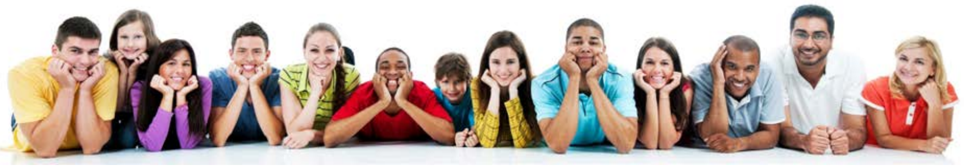

Who is affected by CHH?

Both men and women are affected. CHH is more often diagnosed in men. The reason for this is not clear. It may be that many affected women see a gynaecologist and given a prescription for the contraceptive « pill » to have regular periods, yet they may not get a clear diagnosis. Some patients never get an exact diagnosis and may never see a specialist for GnRH deficiency.

How many people are affected?

CHH is rare so an accurate estimate is difficult. We believe CHH occurs in about 1 in 4,000 to 10,000 people. This means that the entire number of patients with CHH in Europe (around 74,000) would fill the Olympic stadium in Berlin Germany.

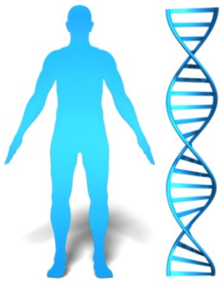

Is CHH inherited? CHH can be hereditary. This means it **can be passed down** through the generations and within families. In many cases, there is no apparent family history. A genetic cause can be identified about half (50%) of the time. Research is ongoing and there is more to discover about the genetic causes of CHH. In most cases, it is difficult for healthcare providers to reliably and accurately predict if a patient will pass CHH on to their children.

Half of patients have a known genetic cause, half have no known genetic cause

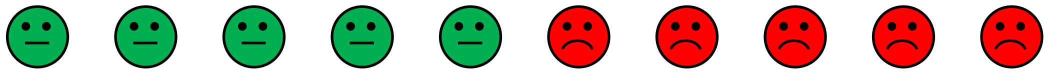

CHH is complicated because in some cases CHH can be caused by the combination of two or more changes (mutations) in different genes. This makes it **challenging to predict if the CHH will be passed on to children**. For one gene, *ANOS1* (*KAL1*) , it is simple to predict. More research is needed to better understand this and improve diagnosis.

How is CHH diagnosed?

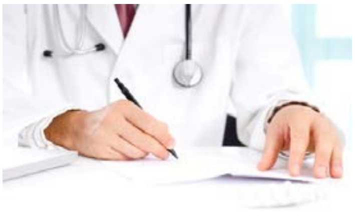

CHH is difficult to diagnose. There is a wide range in the normal timing of puberty. This makes it very difficult to decide if puberty is delayed (but normal), or if it is CHH and needs medical treatment. Regular appointments are needed to follow the progress of puberty. Patients should always be asked about a defective sense of smell. Signs pointing to CHH are:

• **boys -** no signs of puberty by age 16 with low testosterone, LH & FSH.

• **girls -** no breast development by age 14-15 and no menstrual period (bleeding) by age 16-17 with low oestrogen, LH & FSH.

• **Other tests must be done** to make sure there are no other problems causing the low hormone levels. Tests include blood tests and imaging studies (X-rays, ultrasound & MRI)

Are there early signs to look for?
For many people there are no signs of CHH before the failure of puberty.

Some male infants may show signs suggesting CHH, **micropenis or undescended testes** (cryptorchidism).

Micropenis can be treated in infancy / childhood with hormone treatment. Undescended testes should be corrected by surgery early in the first year of life to help future fertility.

There are additional signs, not due to CHH, that happen in some but not all patients.

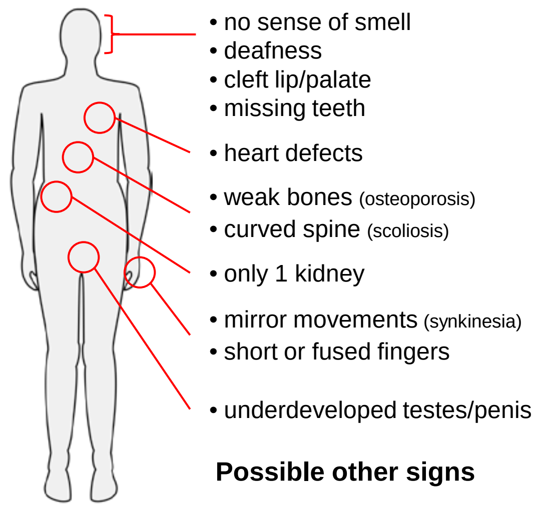

Possible other signs

How would the absence of puberty affect somebody?
Not going through puberty at the same time as peers **can be very stressful and sometimes traumatic**. This can be a major problem for some patients with CHH. The impact can be severe in some cases though it will vary from person to person. The teenage years can be difficult for teenagers who enter puberty at the normal time, but not going through puberty and being left behind by the peer group may result in potential issues including:

- low self-esteem, little confidence
- shyness, difficulty interacting with peers
- anxiety, depression
- poor body image

An abnormal sense of smell can effect your sense of taste and enjoying food. People may not be able to detect gas (or when foods spoil). Some may be concerned about body odor.

What can you do?
On the whole, these can be overcome. Psychological counselling and therapy can be useful.

1. **talk with your healthcare provider** - they may not realise that you are having a hard time. They can help you find counselling and support.
2. **connect with other patients -** using online groups (Facebook, RareConnect.org) and face-to-face support groups can be very helpful. These are places where patients can talk about issues that are important to them. Other patients understand what it is like to live with CHH day-to-day and can provide practical advice and support.

What should you do to be healthy?
Having CHH should not shorten your life. There are things that you can do to stay healthy

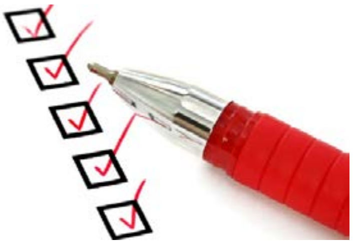

- have ongoing healthcare and visits
- take your treatment as prescribed
- eating a healthy diet
- get regular exercise
- don't smoke tobacco

2016: www.gnrhnetwork.eu / andrew.dwyer@chuv.ch

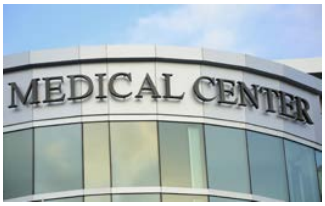

Is CHH curable?

Currently, **there is no cure for CHH**. It is very difficult to cure a congenital (genetic) disorder. Research is ongoing to see if we can restore GnRH production from the hypothalamus. This research is still in a very early stage but it is hoped that this will be possible in the future.

Are there treatments available for CHH?

Treatments are available to develop the outward signs of puberty - such as beard growth for men and breast development in women. Special hormone treatments also available to help develop fertility in most cases.

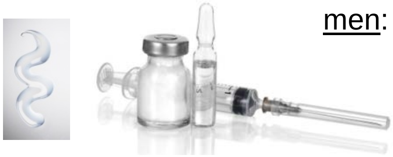

men: **Testosterone** (gel applied to the skin or injections) is the most common treatment to develop signs of puberty. Treatment induces growth, deepening of the voice, beard growth, penis growth, sex drive (libido) and sexual function – but not fertility.

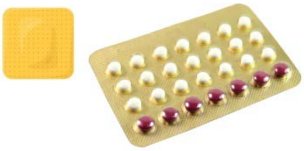

women: low dose oestrogen (patch or pills) induces growth and helps develop breasts, sex drive (libido) and a feminine figure, combined with progesterone this causes regular menstrual periods (bleeding) – but not fertility.

When treatment is first started, the **dose adjustment is gradual**. This can be frustrating for some people who might expect rapid results. However, it is important to maximise growth (and breast development in females)

A few people with CHH recover after treatment and can produce normal hormone levels. This is called a reversal. The reasons for this are not understood and we are unable to predict who will have such a reversal. **This recovery does not always last. So, it is important to be followed closely by a healthcare provider.**

About 1 in 10 (10%) patients with CHH have a reversal

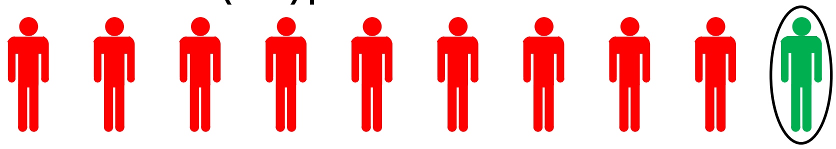

Can a person with CHH become fertile?

Yes, **CHH is a treatable form of infertility.** The majority - but not all - people with CHH can become fertile with **specialised hormone treatment.**

- specialised treatment can be either hormone injections several times a week or wearing a portable GnRH pump (like the pump used for diabetes).
- fertility treatment requires care from experienced specialists (**reproductive endocrinologists**)
- sometimes (but not always) assisted fertility is needed, such as *in vitro* fertilisation (IVF).

Fertility: Overall, 3 out of 4 patients (75%) are successful

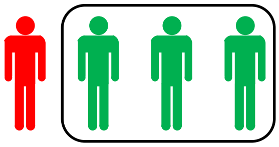

Men: it can take up to 2 years to develop sperm

Women: in some cases, fertility is achieved in a few months

2016: www.gnrhnetwork.eu / andrew.dwyer@chuv.ch

Are there any risks if CHH is not treated?
While testosterone and oestrogen are not vital hormones for life, their absence (or deficiency) can seriously impact **health, sexual function and quality of life.**

In men & women
- increased risk for **low bone density (osteoporosis)** – this means that the bones are weak and there is a higher than normal risk for fractures. If CHH is not treated, osteopeorosis can affect a person at any age. Some people with CHH need extra treatment for osteoporosis.
- **decreased sexual function and desire** is a result of low hormone levels
- **fatigue, feeling down, and depression** are common among patients off treatment

Men without treatment are also at higher risk of having metabolic problems such as getting pre-diabetes or diabetes. **These risks can be decreased by staying on treatment**.

Key Points:

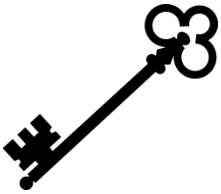

- CHH is rare condition caused by GnRH deficiency
- CHH results in absent puberty and infertility
- CHH should not shorten your life
- CHH is difficult to diagnose
- many people are not diagnosed until late in teenage years or early adulthood
- unlike many rare diseases, there are treatments available
- you need life long treatment and regular visits with your doctor
- talk with your doctor about treatment options to decide what is best for you
- stopping treatment has negative consequences on your health and wellbeing
- fertility is possible in most cases with special hormone treatment
- you can pass CHH to your children, so genetic counselling is recommended
- CHH is psychologically difficult for some people
- you can find support from your healthcare providers and patient groups

Helpful reference
http://www.nature.com/nrendo/journal/v11/n9/full/nrendo.2015.112.html

Helpful websites
http://www.gnrhnetwork.eu/hhn_home/hhn-patients_familles_gnrh_deficiency_kallmann_syndrome/hhn-onlinesupportgroups.htm

https://www.rareconnect.org/en/community/kallmann-syndrome

https://www.facebook.com/KallmannSyndrome/

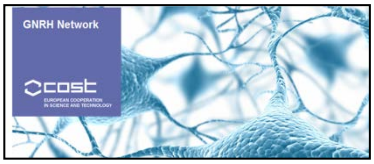

Какво трябва да знаете за вродения хипогонадотропен хипогонадизъм (ВрХХ) и синдрома на Kallmann (СК)

Какво е ВрХХ?

ВрХХ се предизвиква от **дефицит на ГнРХ (гонадотропин рилизинг хормон) .** Съчетанието му с липса на обоняние (аносмия) е известно като олфакто-генитален синдром или синдром на Kallmann (СК). Всички те се диагностицират и лекуват по един и същи начин.

Какви са функциите на ГнРХ?

ГнРХ е хормон от основно значение за половото развитие, пубертета и фертилитета.

1. ГнРХ се отделя от хипоталамуса, област в мозъка, която контролира различни телесни функции.

2. ГнРХ действа пряко на хипофизната жлеза (жлеза в мозъка, голяма приблизително колкото грахово зърно) за отделяне на два хормона: лутеинизиращ хормон (ЛХ) и фоликулостимулиращ хормон (ФСХ).

3. ЛХ и ФСХ имат значение за пубертета и правят фертилитета възможен

• при мъжете: ЛХ и ФСХ стимулират тестисите да произвеждат тестостерон и сперматозоиди

• при жените: ЛХ и ФСХ стимулират яйчниците да образуват естрогени и прогестерон, които са важни за фертилитета.

Какво се получава, ако няма ГнРХ?

Пълният или частичен недостиг на ГнРХ води до липса или значително намаление на ЛХ и ФСХ . Така пубертетът не може да настъпи и се стига до безплодие (вж фигурата по-долу). Това означава, че:

•при мъжете: 1) тестисите не растат, 2) тестостерон не се произвежда в нормални количества, и 3) сперматозоиди не се развиват.

•при жените: 1) въпреки че има яйцеклетки, те не се развиват или не нарастват и не се отделят (овулация), 2) естрогени и прогестерон не се произвеждат и 3) не настъпва менструално кървене.

Белези на неотключен пубертет при подрастващи и млади хора

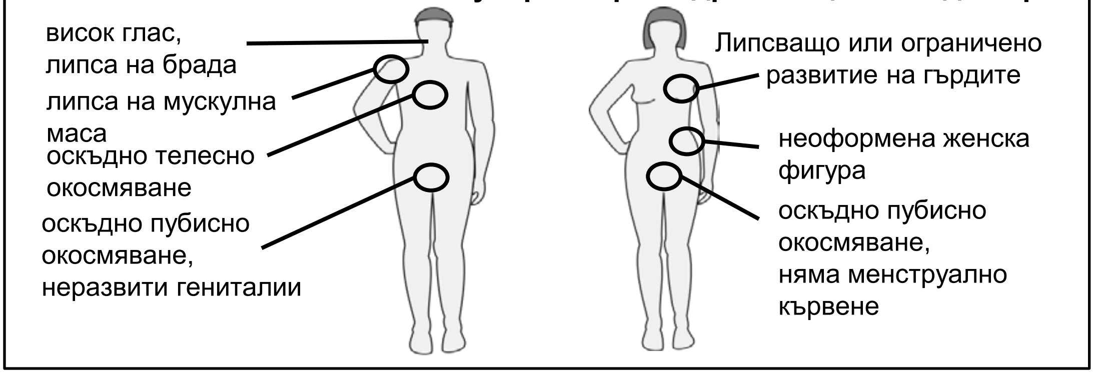

Тези проблеми почти винаги се установяват при раждане (наречени вродени). Но диагнозата често се поставя в тийнейджърските години или в началото на зрялата възраст, когато пубертетът не започва. **Важно е да се знае, че това състояние е лечимо. В повечето случаи е необходимо пожизнено заместване с хормони и постоянни грижи.**

Кой е засегнат от ВрХХ и как това нарушение се диагностицира?

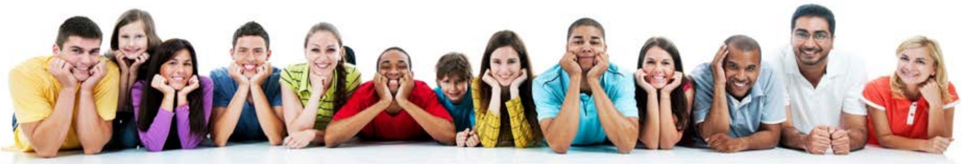

Кой е засегнат от ВрХХ?
Засягат се както мъже, така и жени. ВрХХ по-често бива диагностициран при мъже. Причината за това не е ясна. Може би много от засегнатите жени се преглеждат при гинеколог и им се изписва противозачатъчно « хапче», за да имат редовен цикъл, без да е поставена ясна диагноза. На някои от болните никога не се поставя точна диагноза и те може и да не попаднат при специалист по недостатъчност на ГнРХ.

Колко хора са засегнати?
ВрХХ се среща рядко, затова точната преценка е трудна. Ние предполагаме, че ВрХХ настъпва при около 1 на 4000 до 10000. Това означава, че всички пациенти с ВрХХ от Европа (около 74000) ще се поберат на Олимпийския стадион в Берлин, Германия.

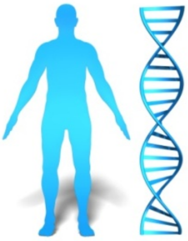

Унаследява ли се ВрХХ? ВрХХ може да бъде наследствен. Това означава, че той може **да бъде предаден** на поколенията и в семейства. В много случаи няма явна фамилна анамнеза. Генетична причина може да бъде открита в около половината (50%) от случаите. Проучванията продължават и ще се открият още факти за генетичните причини за ВрХХ. В повечето случаи е трудно със сигурност и точно да се предвиди дали даден пациент ще предаде ВрХХ на своите деца.

Генетична причина се открива само при половината от болните

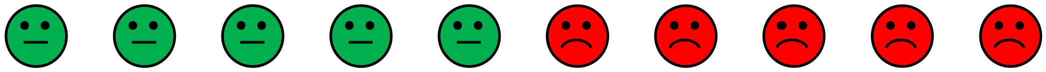

ВрХХ е усложнен, защото в някои случаи ВрХХ може да е предизвикан от комбинацията на две или повече промени (мутации) в различни гени. По тази причина е **затруднено предвиждането дали ВрХХ ще бъде предаден на децата**. За един ген, ANOS1 (KAL1) не е трудно да се предвиди.Още проучвания са потребни, за да се изясни това и да се подобри диагнозата.

Как се диагностицира ВрХХ?
ВрХХ е труден за диагностициране. Границите за нормалното време за пубертета са широки. Поради това е трудно да се реши дали пубертетът закъснява (но е нормален), <u>или</u> се отнася за ВрХХ и се налага лечение. Необходими са редовни прегледи за проследяване на развитието на пубертета. Пациентите трябва винаги да бъдат разпитвани за нарушение на обонянието.Признаци, говорещи за ВрХХ са:

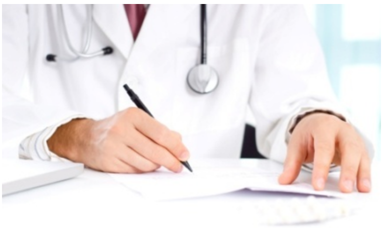

•**момчета –** никакви признаци на пубертет на възраст 16 г. с ниски тестостерон, ЛХ и ФСХ.
•**момичета –** никакво развитие на гърдите на възраст 14-15 г. и липса на менструация (кървене) на възраст 16-17 г. с ниски естрогени, ЛХ и ФСХ.

• **Други изследвания трябва да се извършат,** за да е сигурно, че няма други проблеми, които да са причина за ниските хормонални нива. Те включват кръвни тестове и образни изследвания (рентген, ултразвук и ЯМР)

Има ли ранни признаци, които да се търсят?
За много хора няма признаци на ВрХХ преди отпадането на пубертета.

При някои момченца може да има признаци, предполагащи ВрХХ, **микропенис или недесцендирали тестиси** (крипторхизъм). Микропенис може да бъде третиран в детството (и в ранното) с хормонални средства. Недесцендирали тестиси трябва рано да се коригират хирургично в първата година от живота, за да се подпомогне бъдещият фертилитет.
Има допълнителни признаци,които не се дължат на ВрХХ и се появяват при някои, но не при всички пациенти.

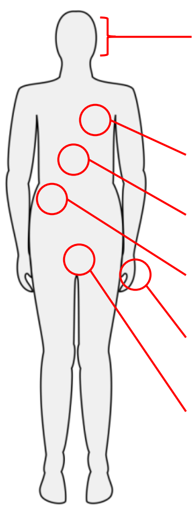

Как липсата на пубертет се отразява върху някои?
Да не се прекара пубертет по същото време като връстниците, **може да бъде много стресиращо и понякога да причини травма**. Това е най-важната последица за болните с ВрХХ. Въздействието може да бъде тежко в някои случаи, макар че от човек до човек може да има разлики. Тийнейджърските години може да бъдат труден период за младежите, при които пубертетът настъпва своевременно, но да не се прекара пубертета и да се изостане от връстниците може да има потенциални последици:

- понижено самочувствие, слаба увереност
- стеснителност, трудно общуване с връстниците
- страх, депресия
- лош образ на тялото

Нарушеното обоняние може да засегне вашия вкус и удоволствие от храната. Хората може да не са в състояние да открият газ (или да не разберат, че храната е развалена). Някои може да са обезпокоени за телесната миризма.

Какво може да направите?
Като цяло, това може да се преодолее. Психологични консултации и терапия могат да са от полза.

1.говорете със здравните специалисти – те могат и да не си дават сметка, че сте в затруднено положение.Те могат да ви помогнат да получите съвет и подкрепа.

2.свържете се с други пациенти –он-лайн групи (Facebook,RareConnect.org) и групи за подкрепа face-to-face могат много да помогнат. Това са места, където пациентите могат да поговорят за проблеми, важни за тях. Други болни разбират какво е да се живее с ВрХХ всеки ден и могат да дадат практически съвет и да окажат подкрепа.

Какво би трябвало да правите, за да сте здрав?
ВрХХ не скъсява вашия живот. Има неща, които може да правите, за да бъдете здрав

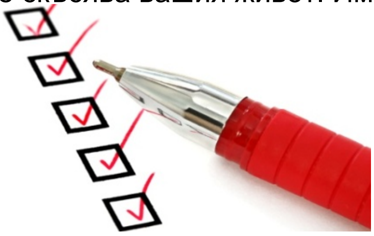

- редовни медицински консултации
- лекувайте се, както ви е предписано
- съблюдавайте здравословна диета
- извършвайте редовно упражнения
- не пушете тютюн

2017: www.gnrhnetwork.eu / andrew.dwyer@chuv.ch

Какви лечения има за ВрХХ?

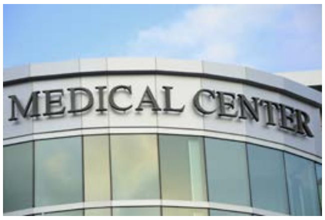

Лечим ли е ВрХХ?

Понастоящем **излекуването на ВрХХ е невъзможно**. Много е трудно да се излекува вродено (генетично) нарушение. Извършват се изследвания да се види дали е възможно възстановяването на продукцията на ГнРХ от хипоталамуса. Тези проучвания са все още в началото, но има надежда, че това ще стане възможно в бъдеще.

Разполагаме ли със средства и начини за третиране на ВрХХ?

Има средства за развитието на външни признаци на пубертета - като брада при мъжете и развитие на гърдите при жените. Има и специални хормонални средства за постигане на фертилитет в повечето случаи.

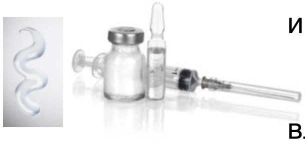

• при мъжете: **тестостерон** (гел, прилаган по кожата или инжекции) е най-обичайното третиране за предизвикване на пубертетни признаци. Лечението предизвиква растеж, промени в гласа, растеж на брада, растеж на пениса, полово влечение (либидо) и сексуална функция – но не и фертилитет.

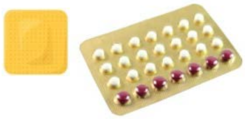

•При жените: ниско дозиран естроген (лепенки или таблетки) предизвиква растеж и спомага за развитието на млечните жлези, полово желание (либидо) и женствена фигура, в комбинация с прогестерон той предизвиква редовните месечни цикли (кървене) – но не фертилитет.

След като първоначално се започне с лечението, **дозите се нагласяват постепенно**. Това е смущаващо за някои, които очакват бързи резултати. Но е важно да се постигне максималният възможен ръст (и развитие на гърдите при жените).

Някои хора с ВрХХ се възстановяват след лечение и могат да поддържат нормални хормонални нива. Това се нарича преобръщане. Причините за това не са изяснени и не е възможно да се предположи кой ще има такова преобръщане. **Това възстановяване не винаги е трайно. Ето защо е важно пациентът да бъде наблюдаван редовно от специалист.**

Около 1 на 10 (10%) пациенти с ВрХХ имат преобръщане

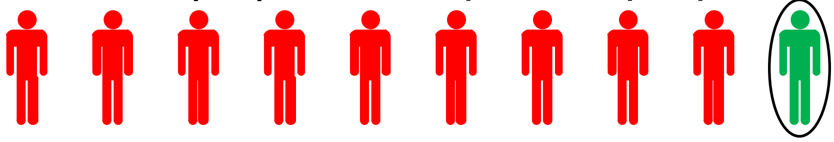

Може ли човек с ВрХХ да стане фертилен?

Да, **ВрХХ е лечима форма на безплодие.** Повечето – но не всички - с ВрХХ могат да станат фертилни **със специализирано хормонално лечение.**

- специализираното лечение се състои в прилагане на хормони в инжекции няколко пъти седмично или в преносима помпичка за ГнРХ (като използваните при диабет).
- лечението за фертилитет се извършва от опитни специалисти (**репродуктивни ендокринолози**)
- понякога (но не винаги) се налага асистирана репропродукция, напр. оплождане ин витро

Фертилитет: Общо взето, при 3 от 4 пациенти (75%) има успех

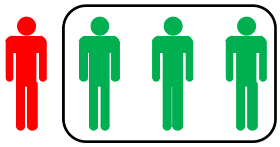

мъже: може да са необходими до 2 години за развитие на сперматозоиди

жени: в някои случаи, фертилитет се постига за няколко месеца

Има ли някакъв риск ако ВрХХ не се лекува?
Макар от тестостерона и естрогените да не зависи животът, липсата им (или недостигът им) могат да дадат сериозни отражения на **здравето, сексуалната функция и качеството на живот.**

При мъжете и жените

•повишен риск за **ниска костна плътност (остеопороза)** – това означава, че костите са крехки и рискът от фрактури е по-висок от нормалния. Ако ВрХХ не се лекува, остеопорозата може да се изяви във всяка възраст. Някои хора с ВрХХ имат нужда от допълнително лечение за остеопороза.

•**намалена сексуална функция и желание** са резултат от ниските хормонални нива

•**умора, чувство за отпадналост и депресия** се срещат често при пациенти без лечение**.**

Мъже без лечение също са с повишен риск да развият метаболитни проблеми като пре-диабет и диабет. **Тези рискове могат да се намалят, ако болният бъде на лечение.**

Ключови изводи:

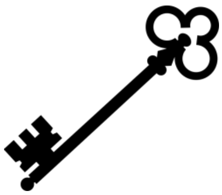

• ВрХХ е рядко състояние, причинява се от дефицит на ГнРХ

• ВрХХ води до липса на пубертет и до безплодие

• ВрХХ не би трябвало да скъсява вашия живот

• ВрХХ е труден за диагностициране

• много остават недиагностицирани до края на второто и началото на третото десетилетие от живота

• за разлика от много редки заболявания, за ВрХХ разполагаме с лечение

• трябва да се лекувате пожизнено и редовно да посещавате вашия лекар

• обсъдете с вашия лекар лечебните възможности и решете коя е най-добрата за вас

• спирането на лечението има негативни последствия върху здравето и благополучието ви

• фертилитетът е постижим в повечето случаи със специално хормонално лечение

• можете да предадете ВрХХ на вашите деца, затова се препоръчва генетична консултация

• ВрХХ създава психологични проблеми при някои болни

• можете да намерите подкрепа от здравни специалисти и пациентски групи

Полезен източник
http://www.nature.com/nrendo/journal/v11/n9/full/nrendo.2015.112.html

Полезни уеб страници
http://www.gnrhnetwork.eu/hhn_home/hhn-patients_familles_gnrh_deficiency_kallmann_syndrome/hhn-onlinesupportgroups.htm
https://www.rareconnect.org/en/community/kallmann-syndrome
https://www.facebook.com/KallmannSyndrome/

简体中文

低促性腺激素性腺功能减退症 (CHH) 和卡尔曼综合征 (KS)

什么是低促性腺激素性腺功能减退症（CHH）?

CHH是由于**促性腺激素释放激素（GnRH） 缺乏**导致的先天性疾病。如果同时出现嗅觉减退，则称为嗅觉-性腺综合征（olfacto-genital syndrome）或者卡尔曼综合征（KS）。CHH和KS的诊断和治疗是相同的。

GnRH的功能什么?

GnRH是调控性腺发育、青春期启动和生育的非常重要的激素。

1. GnRH是由下丘脑分泌的。下丘脑是大脑中的一个部分，掌控着机体的许多功能 。
2. 下丘脑分泌的GnRH直接作用于垂体（大脑中像豌豆大小的组织），促进垂体分泌黄体生成素（LH）和卵泡刺激素（FSH）。
3. LH 和 FSH是对青春期启动和生育非常重要的激素
   - 男性: LH和FSH 促进睾丸分泌睾酮和生成精子
   - 女性: LH和FSH 促进卵巢分泌雌激素和孕激素，雌、孕激素对女性生育起着重要作用。

GnRH激素缺乏会有什么结果?

GnRH部分或全部缺乏会造成LH和FSH的缺乏或降低，从而导致:

青春期不发育和不孕不育 (请看下图)。 这就表现为:

- 男性: 1) 睾丸不发育， 2) 不能分泌正常量的睾酮， 3)无法生成精子。
- 女性: 1) 虽然卵巢中存有卵子，但是卵子不能发育成熟 、不能排卵，2) 不能分泌雌、孕激素，3) 闭经。

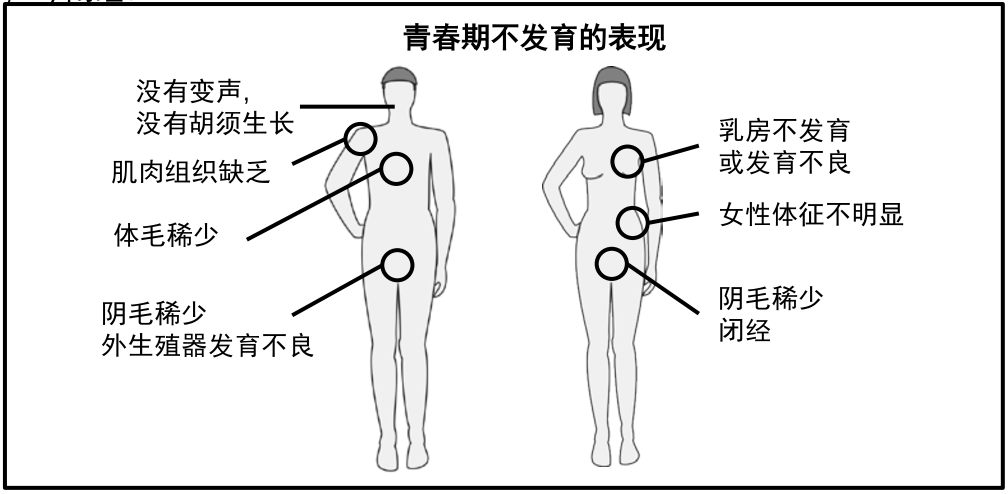

这些问题是从出生就存在了，所以称为先天性疾病，但诊断往往要到十几岁或成年后在患者没有青春期发育时才得到诊断。**重要的是，这是一个可以医治的疾病。大部分病人需要终身治疗和规律随访。**

2017: www.gnrhnetwork.eu / andrew.dwyer@chuv.ch
Translated by Dr. Cheng Xu; Revised by Prof. Linan Cheng

谁会得CHH?

男性和女性都有可能患CHH。但是CHH更多见于男性患者。具体的原因尚不明确。女性患者比较少见的原因可能是由于许多妇女因为闭经到妇科就诊，妇科医生往往给予避孕药治疗诱导月经来潮，但没有进一步排查导致闭经的原因，因此有些妇女从来没有去看过专科医生，未能明确诊断是否患有GnRH缺乏。

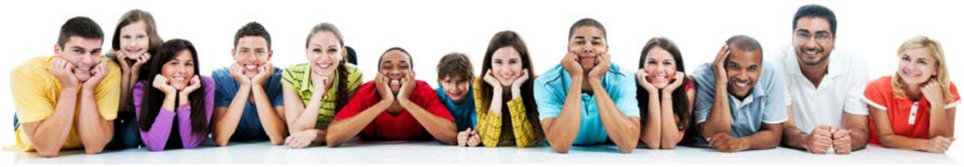

世界上有多少CHH病人?

CHH是个罕见病，所以具体病人人数很难统计。 据报道CHH的发病率在4,000分之一至10,000分之一之间。 如此计算，全欧洲CHH患者人数应该有 74,000，可以坐满柏林奥林匹克

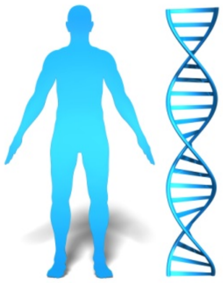

CHH是遗传病么?

CHH是遗传性疾病，这意味着CHH**可能传到下一代**，也有家庭聚集性。大多数CHH患者没有明确的家族史。50%的患者能找到致病基因的突变。科学家正在积极寻找新的CHH的致病基因。在大多数情况下，医生很难准确或精确地预测CHH患者遗传给下一代的风险。

目前为止，半数CHH患者能找到致病基因突变，半数不能

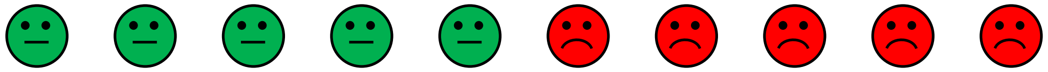

CHH是个复杂的疾病，因为有些病人可以找到不同基因的多个突变。**这点增加了对疾病传到下一代的预测难度**。只有一个致病基因ANOS1（KAL1）是可以准确预测遗传风险。因此需要更多的研究来更好地理解CHH，并且改善其基因诊断的预测性。

CHH如何诊断?

CHH的诊断有一定难度。由于青春期发育时间各不相同，所以有时很难判断是否是青春期迟缓但可以自己发育，<u>还</u>是青春期迟缓不能自己发育。因此对于青春期迟缓的青少年应该进行常规随访，密切观察青春期进展情况。医生需<u>始终</u>询问患者是否存在嗅觉缺失。

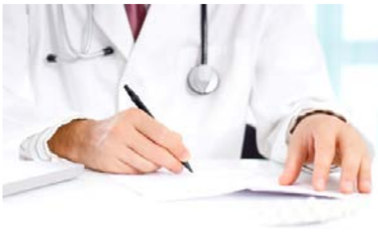

- **男孩 –** 16岁没有青春期体征，睾酮和LH、FSH低下强烈提示CHH。
- **女孩 –** 14-15岁没有乳房发育，16-17岁没有月经，同时伴有雌激素、LH、FSH低下提示CHH。
-
- **其它检查：**包括抽血化验、影像学（X光射片、B超和磁共振）和嗅觉测试，可用于进一步明确诊断

有没有早期的症状?
很多病人在青春期发育前没有其它症状。

一部分男性患者在出生时有**小阴茎或隐睾**，提示存在CHH

小阴茎在婴儿期或儿童期可以用激素治疗。隐睾需要在一岁内进行手术纠正以保证未来的生育功能

部分CHH患者还会有其它相关临床表现，这些临床表现可能与CHH无关。

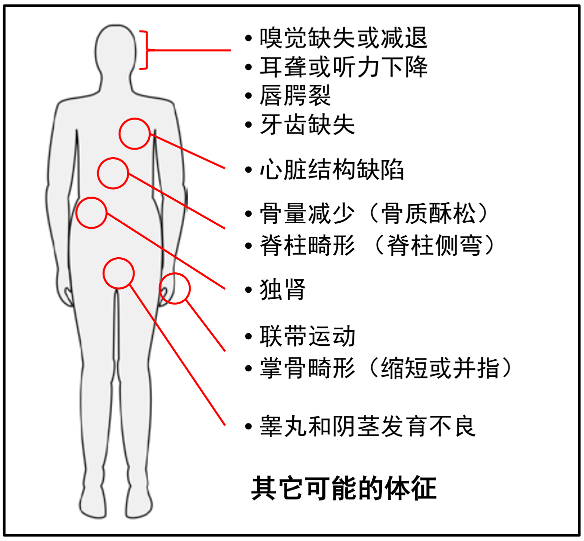

青春期不发育会带来什么影响?
不能和同龄人同时进行青春发育是一件很有压力的事情，也会对心理产生创伤。这个可能是一些CHH患者的最主要问题。对于某些患者，这个影响比较严重，但患者中也存在个体差异。同时，对于青春期正常启动但中途停滞的患者，心理压力可能很大。可能产生的影响包括：

- 自尊心受到影响，自信心不足
- 羞怯、和同伴交流困难
- 焦虑、抑郁
- 对自己体型不满

我该怎么做?
总体来说，这些负面影响是可以疏导和治疗的。及时的内分泌治疗和心理辅导会非常有帮助。

1. **告诉你的主诊医生** – 他们可能并不知道你所经历的心理上的困扰。他们可以给你提供支持和帮助。

2. **与其他患者交流** – 充分利用网上社交组群 （例如，Facebook, RareConnect.org）以及面对面的患者交流活动会有帮助。在这样的场合，患者能够互相交流CHH对他们日常生活的影响，并提供建议和协助。

我该怎么做才能活得健康?
CHH不会缩短你的寿命。但有一些事情可能帮助你变得更健康。

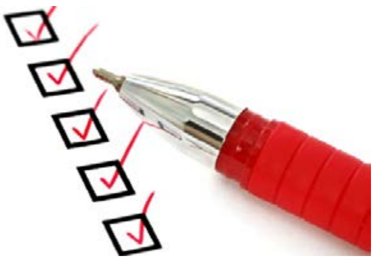

- 有长期的医疗随访
- 按医嘱进行治疗
- 均衡饮食
- 规律运动
- 不吸烟

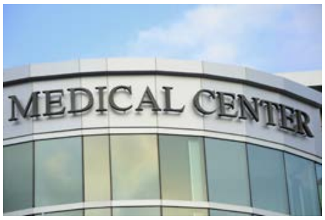

CHH可以治愈么?

目前为止，**CHH还不能治愈**。治愈先天性遗传病是非常困难的。目前进行的医学研究旨在恢复下丘脑分泌GnRH的功能，此类研究尚在早期阶段，尚没有应用于临床。

CHH有哪些治疗?

目前存在的治疗方案能够促使第二性征的发育 – 例如男性胡须生长或女性乳房发育。特殊的激素治疗也能够使大多数患者恢复生育能力。

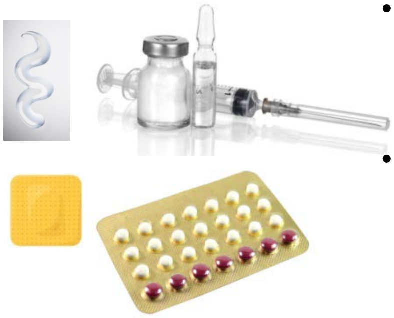

- <u>男性</u>: **睾酮 治疗**（直接应用于皮肤的凝胶剂或肌肉注射针剂）是最常用　于促进第二性征发育的治疗手段。治疗能够促进生长、变声、胡须生长、阴茎生长、性欲和性功能 – 但是不能治疗不孕不育。
- <u>女性</u>: 低剂量雌激素治疗（口服药或皮肤贴剂）能够促进生长、乳房发育、性欲和女性特征。雌孕激素联合治疗能够诱导规律月经 – 但是不能治疗不孕不育。

初始治疗时，需要**逐渐调整剂量**。某些患者希望快速的疗效，觉得这样治疗不够立竿见影。但是逐渐增加剂量的原因是要最大程度地促进身高的生长，以及女性乳房发育。

少数CHH患者经过一段时间治疗后能够恢复性腺轴功能，自己产生足够的性激素。这个情况称为“恢复”（reversal）。“恢复”的原因和机制目前不明，因此无法预测哪类病人能够“恢复”。另外，这种“恢复”可能持续不长的时间。因此即使是“恢复”的患者，还需要医生的密切随访。

每10个CHH患者中有1个能够“恢复”（10%）

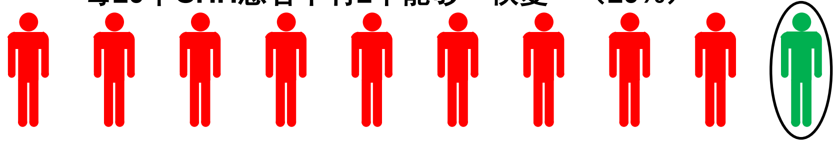

CHH患者能通过治疗恢复生育能力吗?

能。 **CHH患者的不孕不育是可以治疗的。**大部分患者能够通过**特殊的激素治疗**达到生育目的。

- 特殊的激素治疗可以是每周多次的皮下注射药物，或者携带GnRH注射泵（类似于糖尿病患者的胰岛素注射泵）。
- 不孕不育的治疗需要有经验的专科医生（**生殖内分泌科医生**）
- 有时需要辅助生育技术，例如试管婴儿（IVF）。

生育: 总体来说，每4个CHH患者中3个能通过治疗恢复生育能力（75%）

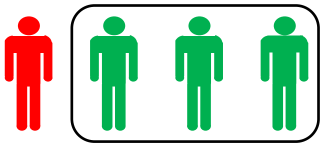

<u>男性</u>: 需要2年的疗程产生精子

<u>女性</u>: 一些患者，几个月治疗就能恢复生育

如果不治疗CHH会有什么后果?

虽然睾酮和雌激素不是生存不可缺少的激素，但是缺乏性激素会严重影响**健康、性功能和生活质量**。

<u>男性和女性：</u>

- 增加**骨质疏松**的风险 – 意味着骨骼不强健且有骨折风险。如果CHH不进行治疗，骨质疏松可能在任何年龄发生。一些患者需要接受其它骨质疏松的治疗。
- 性激素缺乏导致的**性功能下降和性欲低下**
- 未治疗的患者常常有**疲劳、情绪低落和抑郁**的情况

未治疗的CHH男性患者有更高的代谢性疾病（例如胰岛素抵抗或糖尿病）的风险。**激素替代治疗可以减少此类疾病的发生风险。**

要点:

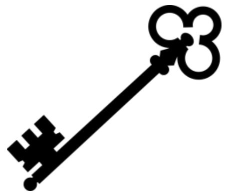

- CHH是一个罕见的疾病，病因是GnRH缺乏
- CHH导致青春期不发育和不孕不育
- CHH<u>并不</u>缩短你的预期寿命
- 诊断CHH并不容易
- 许多患者要等到成年后才得到诊断
- 跟很多罕见疾病不同，CHH有多种治疗方案
- 你需要终身治疗和定期医疗随访
- 你需要积极地和你的主诊医生沟通，找到最合适你的治疗方案
- 停止治疗对你的健康和生活质量会有负面的影响
- 对大多数患者，应用特殊激素治疗后生育是有可能的
- 你有可能将CHH传给你的下一代，所以需要遗传学咨询
- CHH对一些患者来说是一个心理负担
- 你可以寻求主诊医生的帮助或CHH患者群的帮助

参考文献（英文）

http://www.nature.com/nrendo/journal/v11/n9/full/nrendo.2015.112.html

有用的网址（英文）

http://www.gnrhnetwork.eu/hhn_home/hhn-patients_familles_gnrh_deficiency_kallmann_syndrome/hhn-onlinesupportgroups.htm

https://www.rareconnect.org/en/community/kallmann-syndrome

https://www.facebook.com/KallmannSyndrome/

低促性腺激素性腺功能減退症(CHH) 和卡爾曼綜合徵(KS)

什麼是低促性腺激素性腺功能減退症 （CHH）？

CHH 是由於**促性腺激素釋放激素 （GnRH） 缺乏**導致的先天性疾病。 如果同時出現嗅覺減退，則稱為嗅覺-性腺綜合徵（olfacto-genital syndrome）或者卡爾曼綜合徵（KS）。CHH和KS的診斷和治療是相同的。

GnRH的功能是什麼？

GnRH是調控性腺發育、 青春期啟動和生育的非常重要的激素。

1. GnRH是由下丘腦分泌的。下丘腦是大腦中的一個部分，掌控著機體的許多功能。
2. 下丘腦分泌的GnRH 直接作用於垂體 （ 大腦中像豌豆大小的組織 ）， 促進垂體分泌黃體生成素 （LH）和卵泡刺激素（FSH）。
3. LH 和 FSH是對青春期啟動和生育非常重要的激素
   • 男性: LH和FSH 促進睾丸分泌睾酮和生成精子
   • 女性: LH和FSH 促進卵巢分泌雌激素和孕激素，雌、孕激素對女性生育起著重要作用 。

GnRH 激素缺乏會有什麼結果?

GnRH 部分或全部缺乏會造成 LH和FSH 的缺乏或降低，從而導致 ：
青春期不發育和不孕不育 (請看下圖) 。這就表現為:

- 男性: 1) 睾丸不發育， 2) 不能分泌正常量的睾酮， 3) 無法生成精子。
- 女性: 1) 雖然卵巢中存有卵子， 但是卵子不能發育成熟、不能排卵，2) 不能分泌雌、孕激素，3) 閉經。

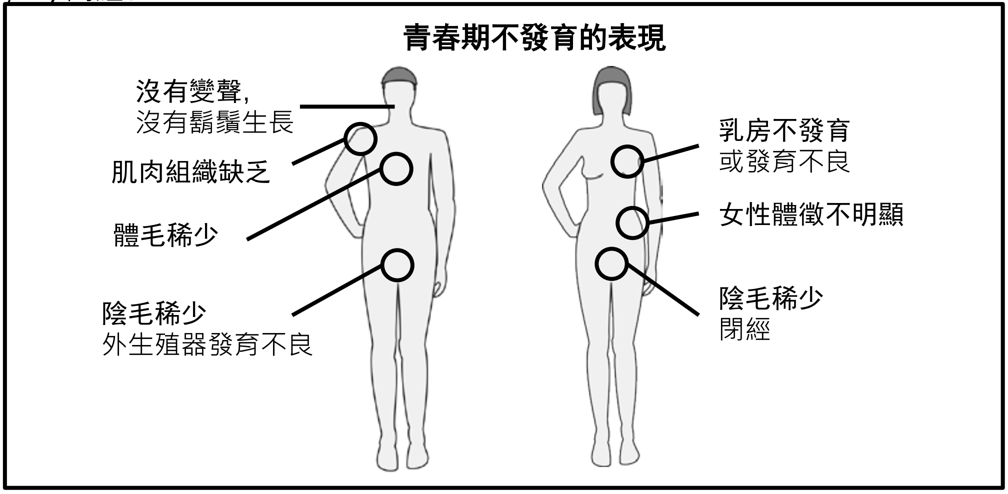

這些問題是從出生就存在了，所以稱為先天性疾病，但診斷往往要到十幾歲或成年後在患者沒有青春期發育時才得到診斷。 **重要的是，這是一個可以醫治的疾病。大部分病人需要終身治療和規律隨訪。**

2017: www.gnrhnetwork.eu / andrew.dwyer@chuv.ch
Translated by Dr. Cheng Xu; Revised by Prof. Linan Cheng

誰會得CHH?

男性和女性都有可能患CHH。但是CHH更多見於男性患者。具體的原因尚不明確。女性患者比較少見的原因可能是由於許多婦女因為閉經到婦科就診，婦科醫生往往給予避孕藥治療誘導月經來潮，但沒有進一步排查導致閉經的原因，因此有些婦女從來沒有去看過專科醫生，未能明確診斷是否患有GnRH缺乏。

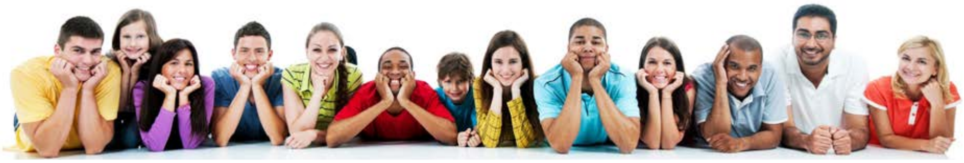

世界上有多少CHH病人?

CHH是個罕見病，所以具體病人人數很難統計。據報導CHH的發病率在4,000分之一至10,000分之一之間。如此計算，全歐洲CHH患者人數應該有 74,000，可以坐滿柏林奧林匹克

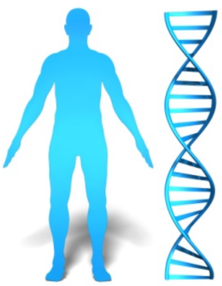

CHH是遺傳病麼?

CHH是遺傳性疾病，這意味著CHH**可能傳到下一代**，也有家庭聚集性。大多數CHH患者沒有明確的家族史。50%的患者能找到致病基因的突變。科學家正在積極尋找新的CHH的致病基因。在大多數情況下，醫生很難準確或精確地預測CHH患者遺傳給下一代的風險。

目前為止，半數CHH患者能找到致病基因突變，半數不能

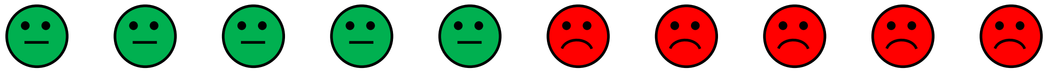

CHH是個複雜的疾病，因為有些病人可以找到不同基因的多個突變。這點增加了對疾病**傳到下一代的預測難度**。需要更多的研究來更好地理解CHH，並且改善其基因診斷的預測性。

CHH如何診斷?

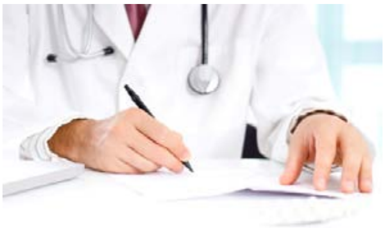

CHH的診斷有一定難度。由於青春期發育時間各不相同，所以有時很難判斷是否是青春期遲緩但可以自己發育，<u>還是</u>青春期遲緩不能自己發育。因此對於青春期遲緩的青少年應該進行常規隨訪，密切觀察青春期進展情況。 **醫生需<u>始終</u>詢問患者是否存在嗅覺缺失。**

- **男孩 –** 16歲沒有青春期體徵，睾酮和LH、FSH低下強烈提示CHH。
- **女孩 –** 14-15歲沒有乳房發育，16-17歲沒有月經，同時伴有雌激素、LH、FSH低下提示CHH。
- **其他檢查：** 包括抽血化驗、影像學(X光攝片、B超和磁共振)和嗅覺測試,可用於進一步明確診斷

有没有早期的症状?
很多病人在青春期發育前沒有其他症狀。

一部分男性患者在出生時有**小陰莖或隱睾**,提示存在CHH

小陰莖在嬰儿期或兒童期可以用激素治療。隱睾需要在一歲內進行手術糾正以保證未來的生育功能

部分CHH患者還會有其他相關臨床表現，這些臨床表現可能與CHH無關。

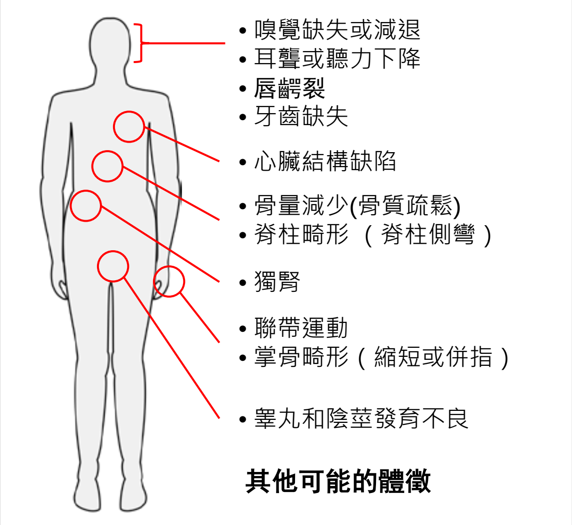

青春期不發育會帶來什麼影響?
不能和同齡人同時進行青春發育是一件很有壓力的事情，也會對心理產生創傷。這個可能是一些CHH患者的最主要問題。對於某些患者，這個影響比較嚴重，但患者中也存在個體差異。同時，對於青春期正常啟動但中途停滯的患者，心理壓力可能很大。可能產生的影響包括：

- 自尊心受到影響，自信心不足
- 羞怯、和同伴交流困難
- 焦慮、抑鬱
- 對自己體型不滿

嗅覺異常可能會影響你的味覺或對食物的感官。患者可能不能察覺到有味道的氣體（或腐敗的食物）。有些患者會受到無法察覺體味的困擾。

我該怎麼做?
總體來說，這些負面影響是可以疏導和治療的。及時的內分泌治療和心理輔導會非常有幫助。

1. **告訴你的主診醫生 –** 他們可能並不知道你所經歷的心理上的困擾。他們可以給你提供支持和幫助。

2. **與其他患者交流 –** 充分利用網上社交組群 （ 例如，Facebook, RareConnect.org ）以及面對面的患者交流活動會有幫助。在這樣的場合，患者能夠互相交流CHH對他們日常生活的影響，並提供建議和協助。

我該怎麼做才能活得健康?
CHH不會縮短你的壽命。但有一些事情可能幫助你變得更健康 。

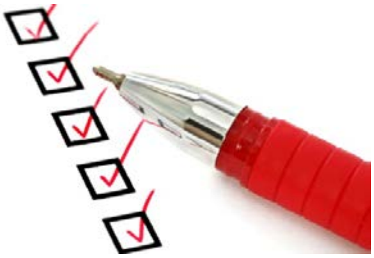

- 有長期的醫療隨訪
- 按醫囑進行治療
- 均衡飲食
- 規律運動
- 不吸煙

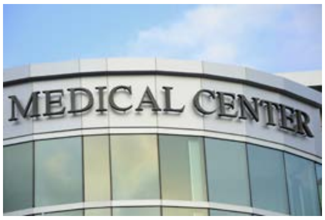

CHH可以治愈麼?

目前為止，CHH還不能治愈。治愈先天性遺傳病是非常困難的。目前進行的醫學研究旨在恢復下丘腦分泌GnRH的功能，此類研究尚在早期階段，尚沒有應用於臨床。

CHH有哪些治療?

目前存在的治療方案能夠促使第二性徵的發育 – 例如男性鬍鬚生長或女性乳房發育。特殊的激素治療也能夠使大多數患者恢復生育能力。

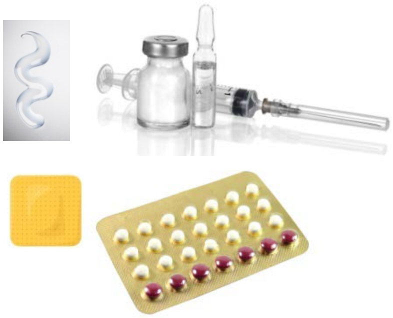

<u>男性</u>: **睾酮治療**（直接應用於皮膚的凝膠劑或肌肉注射針劑）是最常用於促進第二性徵發育的治療手段。治療能夠促進生長、變聲、鬍鬚生長、陰莖生長、性慾和性功能 – 但是不能治療不孕不育。

<u>女性</u>: **低劑量雌激素治療**（口服藥或皮膚貼劑）能夠促進生長、乳房發育、性慾和女性特徵。雌孕激素聯合治療能夠誘導規律月經 – 但是不能治療不孕不育。

初始治療時，需要**逐漸調整劑量**。某些患者希望快速的療效，覺得這樣治療不夠立竿見影。但是逐漸增加劑量的原因是要最大程度地促進身高的生長，以及女性乳房發育。

少數CHH患者經過一段時間治療後能夠恢復性腺軸功能，自己產生足夠的性激素。這個情況稱為"恢復"（reversal）。 "恢復"的原因和機制目前不明，因此無法預測哪類病人能夠"恢復"。另外，這種"恢復"可能持續不長的時間。因此即使是"恢復"的患者，還需要醫生的密切隨訪。

每10個CHH患者中有1個能夠"恢復"（10%）

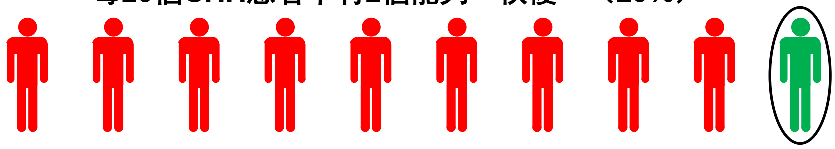

CHH患者能通過治療恢復生育能力嗎?

能。 **CHH患者的不孕不育是可以治療的**。大部分患者能夠通過**特殊的激素治療達到生育目的。**

- 特殊的激素治療可以是每週多次的皮下注射藥物，或者攜帶GnRH注射泵（類似於糖尿病患者的胰島素注射泵）。
- 不孕不育的治療需要有經驗的專科醫生（**生殖內分泌科醫生**）有時需要輔助生育技術，例如試管嬰兒（IVF）。

生育: 總體來說，每4個CHH患者中3個能通過治療恢復生育能力（75%）

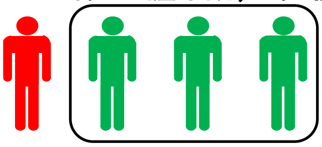

<u>男性</u>: 需要2年的療程產生精子

<u>女性</u>: 一些患者，幾個月治療就能恢復生育

如果不治療CHH會有什麼後果?

雖然睾酮和雌激素不是生存不可缺少的激素，但是缺乏性激素會嚴重影響**健康、性功能和生活質量**。

男性和女性：

- 增加**骨質疏鬆**的風險 – 意味著骨骼不強健且有骨折風險。如果CHH不進行治療，骨質疏鬆可能在任何年齡發生。一些患者需要接受其它骨質疏鬆的治療。
- 性激素缺乏導致的**性功能下降和性慾低下**
- 未治療的患者常常有**疲勞、情緒低落和抑鬱**的情況

未治療的CHH男性患者有更高的代謝性疾病（例如胰島素抵抗或糖尿病）的風險。激素替代治療可以**減少此類疾病的發生風險**。

要點:

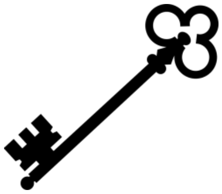

- CHH是一個罕見的疾病，病因是GnRH缺乏
- CHH導致青春期不發育和不孕不育
- CHH並不縮短你的預期壽命
- 診斷CHH並不容易
- 許多患者要等到成年後才得到診斷
- 跟很多罕見疾病不同，CHH有多種治療方案
- 你需要終身治療和定期醫療隨訪
- 你需要積極地和你的主診醫生溝通，找到最合適你的治療方案
- 停止治療對你的健康和生活質量會有負面的影響
- 對大多數患者，應用特殊激素治療後生育是有可能的
- 你有可能將CHH傳給你的下一代，所以需要遺傳學諮詢
- CHH對一些患者來說是一個心理負擔
- 你可以尋求主診醫生的幫助或CHH患者群的幫助

參考文獻（英文）

http://www.nature.com/nrendo/journal/v11/n9/full/nrendo.2015.112.html

有用的網址（英文）

http://www.gnrhnetwork.eu/hhn_home/hhn-patients_familles_gnrh_deficiency_kallmann_syndrome/hhn-onlinesupportgroups.htm

https://www.rareconnect.org/en/community/kallmann-syndrome

https://www.facebook.com/KallmannSyndrome/

Vigtig viden om medfødt hypogonadotrop hypogonadisme (MHH) & Kallmanns syndrom (KS)

Hvad er MHH?

MHH skyldes mangel på hormonet **GnRH** (gonadotropin-frigivende hormon). Kombineret med manglende lugtesans (anosmi) kaldes det for olfacto-genitalt syndrom eller Kallmanns syndrom (KS). MHH og KS. Alle disse former diagnosticeres og behandles på samme måde.

Hvad gør GnRH?

GnRH er et essentielt hormon af betydning for kønslig udvikling, pubertet og fertilitet.

1. GnRH frigives fra hypotalamus, som er et lille område i hjernen, der styrer en række kropsfunktioner.
2. GnRH virker direkte på hypofysen (kirtel lige under hjernen på størrelse med en ært) og frigiver to hormoner: luteiniserende hormon (LH) og follikelstimulerende hormon (FSH).
3. LH & FSH er vigtige for puberteten og er en forudsætning for fertilitet
   • hos mænd: LH & FSH stimulerer testiklernes produktion af testosteron og sæd
   • hos kvinder: LH & FSH stimulerer æggestokkenes ægmodning og produktion af østrogen & progesteron, som er vigtige for fertilitet.

Hvad sker der, hvis der mangler GnRH?

Hel eller delvis GnRH-mangel resulterer i manglende eller meget lave niveauer af LH og FSH I blodbanen. Det resulterer i **i udebleven pubertet og i barnløshed** (se figuren nedenfor). Det betyder følgende:

- hos mænd: 1) testiklerne vokser ikke, 2) der produceres ikke testosteron, 3) der dannes ingen sæd.
- hos kvinder: 1) der dannes ingen æg - selvom der er æg, vokser de ikke og der er ingen ægløsning (ovulation), 2) østrogen og progesteron dannes ikke og 3) der er ingen menstruationsperiode (blødning).

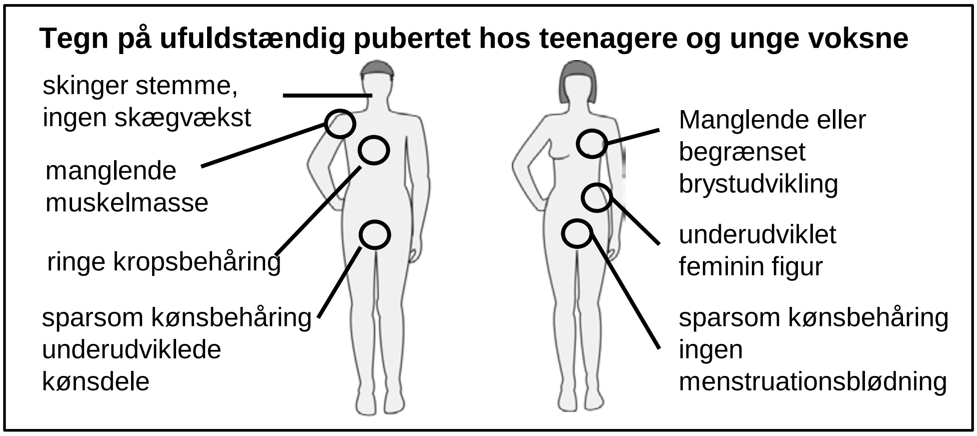

Disse problemer er næsten altid medfødte, men diagnosen stilles oftest først i teenageårene eller de tidlige voksenår når puberteten ikke er startet. **Men tilstanden kan behandles. I de fleste tilfælde kræver den årelang hormon erstatning og løbende behandling.**

2017: www.gnrhnetwork.eu / andrew.dwyer@chuv.ch

Hvem kan få MHH, og hvordan stilles diagnosen?

Hvem kan få MHH?

Både mænd og kvinder kan få MHH. MHH ses oftere hos mænd. Der er ingen entydig forklaring herpå. Det kan måske skyldes, at mange kvinder, som får stillet diagnosen, har fået recept på p-piller for at få regelmæssig menstruation uden nødvendigvis at få stillet en præcis diagnose først. Nogle patienter får aldrig stillet en præcis diagnose og undersøges aldrig af en specialist i GnRH-mangel.

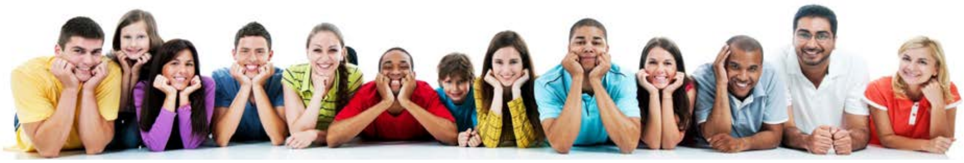

Hvor mange får stillet diagnosen?

MHH er sjælden og et nøjagtigt antal er svært at angive. Efter vores opfattelse forekommer MHH hos ca. 1 ud af 4.000 til 10.000 mennesker. Det vil sige, at det samlede antal patienter med MHH i Europa (ca. 74.000) kan fylde hele det olympiske stadion i Berlin.

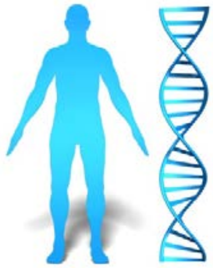

Er MHH arvelig?

MHH kan være arvelig. Det betyder, at **det kan nedarves** i generationer og i samme familie. I visse tilfælde er der ingen direkte familiehistorik. Der kan i cirka halvdelen (50%) af tilfældene fastlægges en genetisk årsag. Der forskes løbende, og de genetiske årsager til MHH er endnu ikke fuldt afdækket. I de fleste tilfælde er det svært for lægen sikkert og præcist at forudsige, om en patient vil overføre MHH til sine børn.

For halvdelen af patienterne er den genetiske årsag kendt, for den anden halvdel er den ukendt

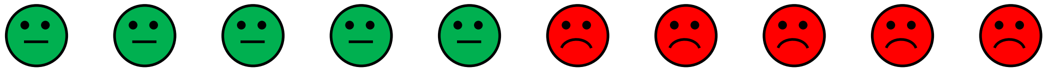

MHH er kompliceret, fordi i nogle tilfælde kan MHH forårsages af en kombination af to eller flere forandringer (mutationer) i forskellige gener. Det gør det **vanskeligt at forudsige, om MHH overføres til børn**. For et enkelt gens vedkommende, *ANOS1 (KAL1*) er det imidlertid let at forudsige. Der er behov for yderligere forskning for bedre at kunne forstå og forbedre diagnosticeringen.

Hvordan stilles MHH-diagnosen?

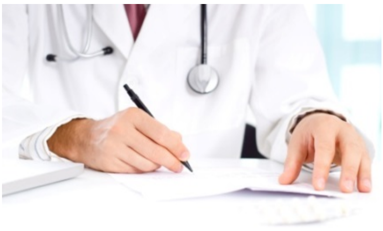

Det er svært at diagnosticere MHH. Timingen af normal pubertet varierer en del. Det gør det svært at afgøre, om puberteten er forsinket (men normal), eller om der er tale om MHH og derfor kræves medicinsk behandling. Der kræves løbende undersøgelser for at følge pubertetsudviklingen. Patienter bør altid udspørges om de har nedsat eller defekt lugtesans. Tegn der peger på MHH:

• **drenge -** ingen tegn på pubertet ved 16 års alderen med lavt testosteron, LH & FSH er tegn på MHH.
piger - ingen brystudvikling ved 14-15 års alderen og ingen menstruationer (blødning) ved 16-17 års alderen med lavt østrogen, LH & FSH er tegn på MHH.

• **Der skal udføres yderligere tests** for at sikre, at der ikke er andre årsager til lavt hormonniveau. Disse tests omfatter blodprøver og billeddiagnostisk undersøgelse (røntgen, ultralyd og MR-skanning)

Er der tidlige tegn, man skal holde øje med?
Hos mange mennesker er der ingen tegn på MHH, før puberteten udebliver.

Nogle drengebørn kan vise tegn på MHH, **mikropenis og ikke-nedsunkne testikler** (kryptorkisme).

Mikropenis kan behandles i spædbarnsalderen / barndommen med hormoner. Ikke-nedsunkne testikler skal behandles kirurgisk tidligt i de første leveår for at afhjælpe fremtidig fertilitet.

Der er andre tegn, der ikke skyldes CHH, som ses hos nogle men ikke alle patienter.

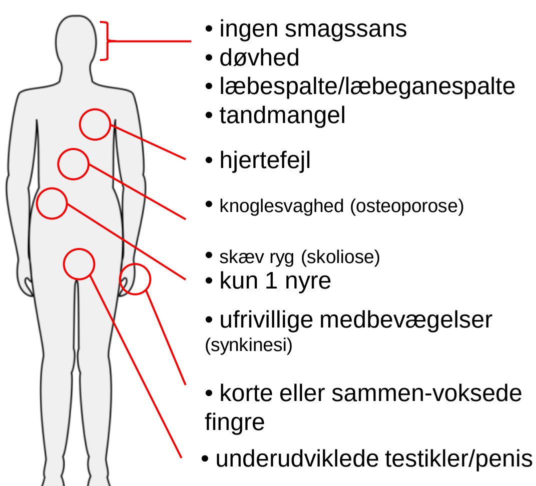

Andre mulige tegn

Hvordan påvirkes man af udebleven pubertet?
Er man ikke gået i puberteten på samme tid som sine jævnaldrende, **kan det være meget stressende og til tider traumatisk**. Dette kan være et meget stort problem for nogle MHH-patienter. Påvirkningen kan i visse tilfælde være kraftig, dog kan det variere fra person til person. Teenageårene kan være svære for alle teenagere, selv hvis man kommer i puberteten til normal tid. Men kommer man ikke i puberteten og bliver man overhalet af sine jævnaldrende, kan det føre til potentielle problemer såsom:

- lavt selvværd, lav selvtillid
- generthed, problemer med interaktion med jævnaldrende
- angst, depression
- ringe kropsbevidsthed

An abnorm lugesans kan påvirke din evne til at smage og nyde mad. Folk kan ikke lugte gas, eller når mad er fordærvet. Nogle kan være bekymret hvis de ikke bemærker egen kropslugt.

Hvad kan du gøre?
Samlet set kan disse problemer løses. Psykologisk vejledning og behandling kan være nyttig. **tal med din læge** - vedkommende ved måske ikke, at du går og har det svært. Lægen kan hjælpe med at finde frem til hjælp og støtte.

1. **kom i kontakt med andre patienter -** online grupper (Facebook, RareConnect.org) og direkte støttegruppemøder kan være nyttige. Dette er steder, hvor patienter kan tale om de problemer, der er vigtige for dem. De andre patienter ved godt, hvad det vil sige at leve dagligt med MHH og kan give praktisk råd og støtte.

Hvad skal du gøre for at holde dig sund og rask?
MHH medfører ikke kortere levetid. Der er ting, du kan gøre for at holde dig rask

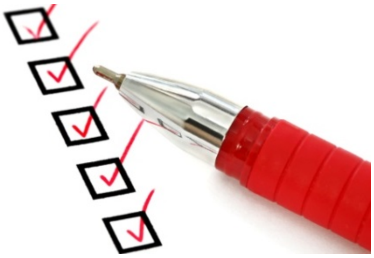

- Regelmæssige besøg hos lægen
- følg den behandling, lægen har ordineret
- spis sundt
- dyrk regelmæssig motion
- undgå rygning

2017: www.gnrhnetwork.eu / andrew.dwyer@chuv.ch

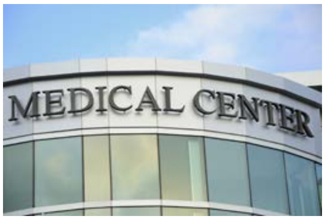

Kan MHH helbredes?

I øjeblikket **kan MHH ikke helbredes**. Det er meget svært at behandle en medfødt (genetisk) sygdom. Der forskes løbende i at prøve at gendanne GnRH-produktionen fra hypotalamus. Forskningen er foreløbig i et tidligt stadie, men man håber, at det i fremtiden bliver muligt.

Findes der nogen behandling for MHH?

Der findes behandling af de ydre pubertetstegn såsom skægvækst hos mænd og brystudvikling hos kvinder. Der findes også særlige hormonbehandlinger, som kan hjælpe med at udvikle fertilitet i de fleste tilfælde.

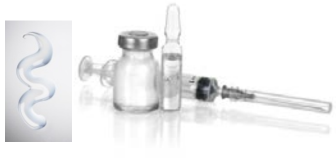

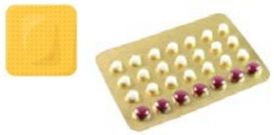

- <u>hos mænd</u>: **testosteron** (gel, som påføres huden, piller eller indsprøjtninger) er den mest almindelige behandling for at udvikle pubertetstegn. Behandlingen fremmer vækst, en dybere stemme, skægvækst, penisvækst og seksualfunktion, men ikke fertilitet.
- <u>hos kvinder</u>: lavdosis østrogen (piller eller plastre) fremmer vækst og understøtter brystudvikling og en kvindelig figur. Kombineret med progesteron giver det regelmæssig menstruation (blødning), men påvirker ikke fertiliteten

Når behandlingen først er startet, **reguleres dosis gradvist**. Det kan være frustrerende for nogle, men det er vigtigt at maksimere væksten (og brystudviklingen hos kvinder)

Nogle få personer med MHH kommer sig efter behandlingen og kan producere hormon på et normalt niveau. Hormonproduktion vender dermed tilbage. Årsagerne kendes ikke, og vi kan ikke forudsige hos hvem, at hormonproduktionen vender tilbage. **Det er ikke altid, at det holder. Derfor skal patienterne følges tæt af en læge.**

Hos omkring 1 ud 10 (10%) patienter med MHH vender hormonproduktionen tilbage

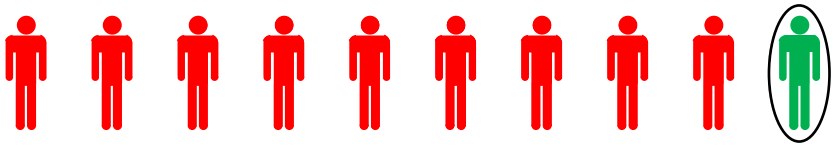

Kan en person med MHH blive fertil?

Ja, **barnløshed som følge af MHH kan behandles.** De fleste, men ikke alle, med MHH kan blive fertile med en **særlig hormonbehandling.**

Fertilitet: Samlet set lykkes det for 3 ud af 4 patienter (75%)

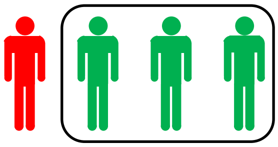

<u>mænd</u>: det kan tage op til 2 år at opnå sædproduktion

<u>kvinder</u>: i visse tilfælde opnås fertilitet allerede efter få måneder

- den særlige behandling kan enten være hormonindsprøjtninger flere gange om ugen eller at bære en GnRH-pumpe (tilsvarende den som diabetespatienter bruger).
- fertilitetsbehandling forudsætter behandling af en erfaren specialist (**endokrinolog med speciale i forplantning**)
- til tider (men ikke altid) er assisteret befrugtning nødvendig, såsom *in vitro* fertilisering (IVF).

Er der risiko ved ikke at behandle MHH?
Testosteron og østrogen er ikke vitale hormoner for dit liv, men ved hel eller delvis mangel kan det have alvorlige indvirkning på **helbredet, seksualfunktionen og livskvaliteten.**

Hos mænd og kvinder

- større risiko for **knogleskørhed (osteoporose)** – det betyder, at knoglerne er svage og at der er en større risiko for brud end normalt. Behandles MHH ikke, kan osteoporose påvirke alle uanset alder. Nogle mennesker med MHH skal have ekstra behandling for osteoporose
- **reduceret seksualfunktion og -lyst** skyldes lavt hormonniveau
- **træthed, tristhed og depression** er normalt hos behandlede patienter

Mænd, der ikke behandles, har også større risiko for at få problemer med stofskiftet såsom diabetes eller forstadier til diabetes. **Disse risici kan reduceres ved at fortsætte behandlingen**.

Centrale punkter:

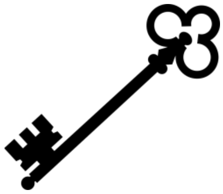

- MHH er en sjælden sygdom forårsaget af GnRH-mangel
- MHH fører til manglende pubertet og til barnløshed
- MHH medfører ikke kortere levetid.
- Det er svært at diagnosticere MHH.
- mange mennesker får ikke nogen diagnose før i de sene teenageår eller tidlig voksenalder
- modsat mange sjældne sygdomme er der forskellige behandlingsmuligheder
- du skal behandles hele livet og regelmæssigt gå til lægen
- tal med din læge om hvilke behandlingsmuligheder, der passer bedst til dig
- stopper du behandlingen, kan det have negative konsekvenser for dit helbred og velvære
- fertilitet er mulig i de fleste tilfælde ved hjælp af hormonbehandling
- du kan overføre MHH til dine børn, så genetisk vejledning anbefales
- MHH er en psykologisk udfordring for nogle mennesker
- du kan få hjælp hos lægen og patientgrupper

Nyttige referencer
http://www.nature.com/nrendo/journal/v11/n9/full/nrendo.2015.112.html

Nyttige websteder
http://www.gnrhnetwork.eu/hhn_home/hhn-patients_familles_gnrh_deficiency_kallmann_syndrome/hhn-onlinesupportgroups.htm

https://www.rareconnect.org/en/community/kallmann-syndrome

https://www.facebook.com/KallmannSyndrome/

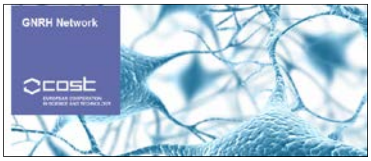

(Nederlands)

Informatie over Congenitaal Hypogonadotroop Hypogonadisme (CHH) & Kallmann Syndroom (KS)

Wat betekent de term CHH?
CHH wordt veroorzaakt door **een tekort aan GnRH (Gonadotroop Releasing Hormoon**). Indien het gepaard gaat met een verminderde mogelijkheid om te ruiken (anosmie),wordt het dikwijls olfacto-genitaal syndroom of Kallmann syndroom (KS) genoemd. De diagnose en behandeling is hetzelfde voor al deze aandoeningen.

Wat is de functie van GnRH?
GnRH is een hormoon dat noodzakelijk is voor de sexuele ontwikkeling,puberteit en vruchtbaarheid.

1. GnRH wordt aangemaakt en afgescheiden door de hypothalamus; de hypothalamus bevindt zich in de hersenen en is belangrijk voor verschillende lichaamsfuncties.
2. GnRH heeft een directe werking op de hypofyse (dit is een erwt-groot kliertje midden in de hersenen) waardoor er Luteinizerend Hormoon (LH) en Follikelstimulerend Hormoon (FSH) aangemaakt wordt.
3. LH & FSH zijn belangrijk voor de puberteitsontwikkeling en de vruchtbaarheid.
   • bij jongens: LH & FSH stimuleren de aanmaak van testosterone en sperma ter hoogte van de teelballen

   • bij meisjes : LH & FSH stimuleren de aanmaak van oestrogenen en progesterone ter hoogte van de eierstokken; deze hormonen zijn noodzakelijk voor de vruchtbaarheid.

Wat gebeurt er als er geen GnRH aangemaakt wordt?
Geheel of gedeeltelijke afwezigheid van GnRH leidt tot afwezige of te lage waarden van LH en FSH. Hierdoor komen **de pubertaire ontwikkeling en de vruchtbaarheid** in het gedrang (zie afbeelding hieronder) :

- <u>Bij jongens:1) de testes worden niet groter 2) er wordt onvoldoende testosterone aangemaakt en 3) er wordt geen sperma aangemaakt</u>
- <u>Bij meisjes:</u> 1) alhoewel er eicellen aanwezig zijn,kunnen deze niet verder ontwikkelen en groeien en treedt er geen eisprong (ovulatie) op, 2) er wordt geen oestrogeen en progesterone aangemaakt en 3) er kunnen geen maandstonden optreden.

Kenmerken bij jongeren en jong volwassenen bij wie de puberteit nog niet opgetreden is

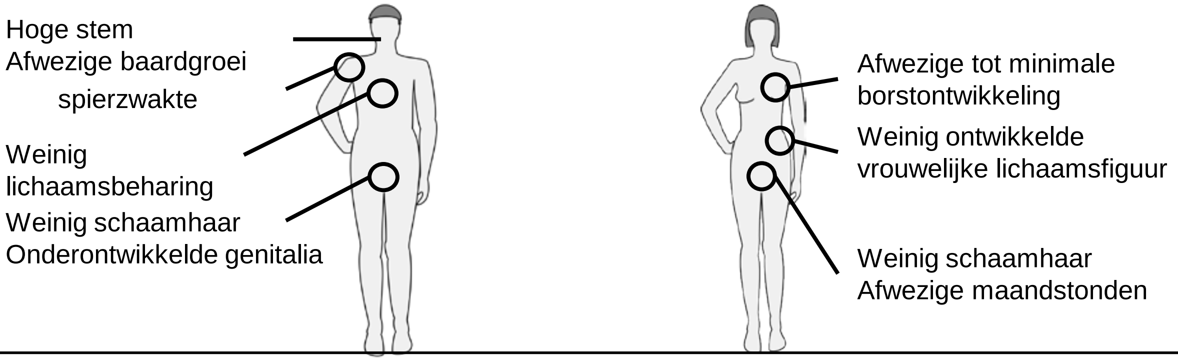

2017: www.gnrhnetwork.eu / andrew.dwyer@chuv.ch

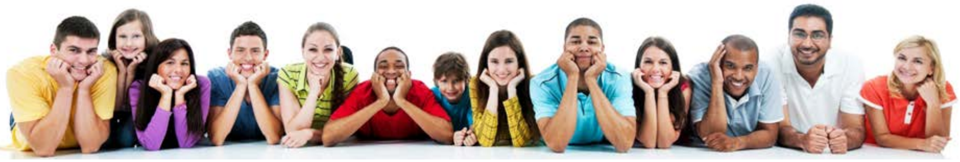

Deze problemen zijn meestal vanaf geboorte aanwezig (congenitaal).Nochtans wordt de diegnose meestal pas gesteld bij tieners of op jongvolwassen leeftijd wanneeer de puberteit niet spontaan gestart is..**Belangrijk om weten is dat deze aandoening kan behandeld worden. In de meeste gevallen moet men levenslang behandeling nemen en** medisch opgevolgd worden.

Wie kan CHH hebben?

CHH kan voorkomen bij zowel jongens als meisjes. Nochtans wordt de diagnose vaker bij jongens gesteld. Het is niet duidelijk waarom dit zo is. Het zou kunnen zijn dat veel aangetaste vrouwen naar de gyecoloog en krijgen daar de contraceptieve « pil » voorgeschreven om regelmatige maandstonden te krijgen, alhoewel er geen duidelijke diagnose werd gesteld en ze nooit een specialist terzake gezien hebben.

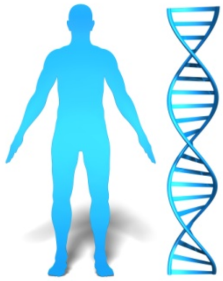

Is CHH erfelijk? CHH kan erfelijk zijn.:het kan over verschillende generaties en binnen bepaalde families **doorgegeven worden**. In vele gevallen komt het niet in de familie voor.Bij ongeveer 50% van de gevallen kan een genetische oorzaak gevonden worden.Er wordt nog veel wetenschappelijk onderzoek verricht om deze genetische oorzaken van CHH te kennen. In de meeste gevallen is het voor de artsen moeilijk om een eerlijk en juist antwoord te geven op de vraag naar erfelijkheid van CHH.

De helft van de patienten heeft een gekende genetische oorzaak,de andere helft heeft een niet gekende genetische oorzaak

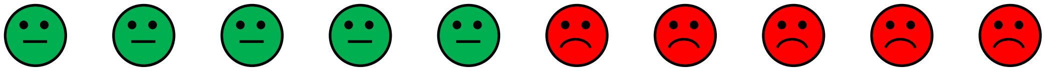

CHH is ingewikkeld omdat de oorzaak in sommige gevallen van CHH in een combinatie van twee of meer veranderingen (mutaties) in verschillende genen kan liggen. Dit maakt het **moeilijk om te voorspellen of CHH kan overgedragen worden op de kinderen**. Voor 1 gen, *ANOS1 (KAL1)* kan dit wel makkelijk voorspeld worden. Meer onderzoek is echter nodig om dit voor andere genen beter te begrijpen en de diagnose beter te kunnen stellen.

Op welke manier wordt de diagnose CHH gesteld ?

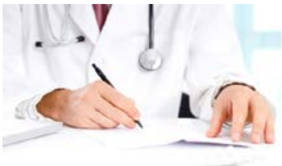

Het is moeilijk om de diagnose CHH te stellen. Normalerwijze bestaat er een brede variatie in het tijdstip waarop de puberteit kan beginnen. Dit maakt het moeilijk om een onderscheid te maken tussen een late (maar nog normale) puberteit, dan wel CHH waarvoor een behandeling nodig is. Het is daarom belangrijk dat er een regelmatige opvolging van de puberteitsontwikkeling gebeurt. Er moet aan patiënten altijd gevraagd worden of de reukzin verstoord is.

Kenmerken die CHH verdacht maken:

jongens – geen tekens van puberteit op 16 jaar met lage testosterone, LH en FSH waarden in het bloed.

meisjes – nog geen borstontwikkeling op 14-15 jaar en nog geen maandstonden op 16-17 jaar met lage warden van oestrogeen, LH en FSH in het bloed.

Bijkomende testen moeten uitgevoerd worden om andere problemen uit te sluiten. Deze onderzoeken bestaan uit uitgebreidere bloedtesten en beeldvorming (RX foto,echo,MRI-scan).

2017: www.gnrhnetwork.eu / andrew.dwyer@chuv.ch

Bestaan er reeds vroege kenmerken?
Bij veel patienten zijn er geen tekens of symptomen in de periode voor de puberteit uitgebleven is.

Sommige jongens kunnen tekens vertonen die aan CHH doen denken, zoals **micropenis of niet-ingedaalde testes (cryptorchidie).**

Micropenis kan met hormonen behandeld worden bij jonge kinderen. Niet ingedaalde testes moeten in het eerste levensjaar chirugisch hersteld worden om de latere vruchtbaarheid te garanderen.

Er kunnen andere kenmerken voorkomen die niet het gevolg zijn van CHH, deze komen niet bij iedereen voor.

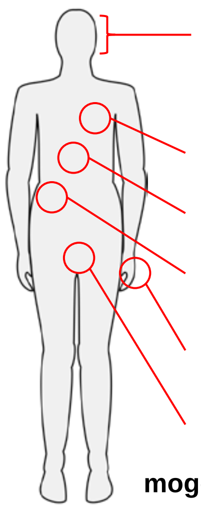

mogelijke andere kenmerken

Op welke manier kan het uitblijven van de puberteit iemands leven beinvloeden ?
Als je nog niet in puberteit bent op de leeftijd dat jou leeftijdsgenoten dat al wel zijn kan jou dit **veel stress bezorgen en erg kwetsend zijn**. Voor sommige patienten is dit een zwaar probleem, maar dit is wisselend. De tienerjaren zijn sowieso moeilijke jaren voor pubers, maar het niet spontaan in puberteit kunnen gaan zoals de leeftijdsgenoten maakt deze periode nog extra zwaar en kan volgende moeilijkheden geven:

- laag zelflvertrouwen
- schaamte,moeilijke omgang met leeftijdsgenoten
- angst, depressie
- laag zelfbeeld

Een abnormale reukzin kan je smaak of het proeven van voedsel beïnvloeden. Mogelijks ben je niet in staat om een gaslek te detecteren. Sommige mensen zijn bezorgd om hun lichaamsgeur.

Wat kan je er aan doen?
De problemen kunnen meestal verholpen worden. Psychologische hulp en behandeling kunnen belangrijk zijn.

1. **Spreek erover met je behandelende arts: Niemand vermoedt misschien hoe moeilijk je het hebt. Samen kunnen jullie zoeken naar een hulpverlener om je te ondersteunen.**
2. **Zoek contact met andere patienten – gebruik de sociale media** (Facebook, RareConnect.org) of zoek gespreksproepen op waar je over de problemen kan spreken die echt belangrijk voor jou zijn. Andere lotgenoten weten wat het betekent dagelijks te moeten leven met CHH en zullen je practische tips en ondersteuning kunnen bieden.

Wat moet je doen om gezond te leven?
CHH zal je levensduur niet verkorten. Je kan er zelf aan werken om zo gezond mogelijk te blijven :

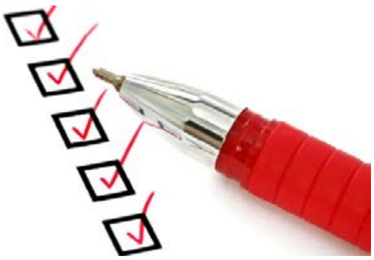

- Laat je regelmatig medisch opvolgen
- Volg je behandelingsschema zoals het werd voorgschreven
- Eet gezonde voeding
- Beweeg en sport regelmatig
- Rook niet

2017: www.gnrhnetwork.eu / andrew.dwyer@chuv.ch

Welke behandeling is mogelijk bij CHH ?

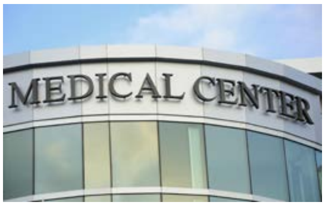

Kan CHH genezen?

Vandaag kunnen we **CHH nog niet genezen.** Het is erg moeilijk om een aangeboren (genetische) aandoening te genezen. Er wordt onderzocht of het mogelijk zou zijn om de hypothalamus opnieuw GnRH te laten aanmaken. Dit onderzoek is nog in een vroeg stadium maar we hopen dat het in de toekomst mogelijk zal worden.

Welke behandeling is mogelijk bij CHH ?

Een behandeling is mogelijk om de uitwendige puberteitskenmerken zoals baardgroei bij jongens en borstontwikkeling bij meisjes zichtbaar te maken. Vaak kan men ook vruchtbaar worden dankzij speciale hormonale middelen.

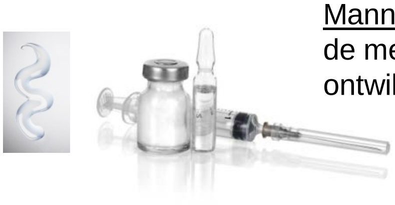

Mannen: **Testosterone** (via gel op de huid ofwel via inspuitingen) is de meeste gebruikte behandeling om de puberteitskenmerken te ontwikkelen. Deze behandeling bevordert de lichaamsgroei, het verdiepen van de stem, de baardgroei, de penis groei, de geslachtsdrift (libido) en het sexueel functioneren maar niet de vruchtbaarheid.

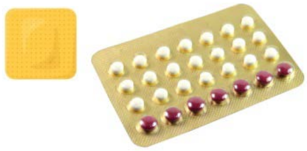

Vrouwen: lage dosis **oestrogenen** (via patch of pillen) kan de lichaamsgroei bevorderen en helpt de borstontwikkeling, de geslachtsdrift (libido) en het verkrijgen van een vrouwelijk figuur; door er progesterone aan toe te voegen zullen er maandstonden kunnen optreden maar geen vruchtbaarheid.

In het begin worden de dosissen geleidelijk verhoogd. Dit kan soms frustrerend zijn als je snel resultaten verwacht. Maar het is belangrijk om op deze manier de groei maximaal te bevorderen (en bij meisjes de borstontwikkeling).

Een klein aantal patienten met CHH kan herstellen en kan achteraf voldoende hormonen zelf aanmaken. We weten nog niet hoe dit komt en kunnen niet voorspellen welke patienten zullen herstellen. Deze zogenaamde « genezing » blijft niet altijd duren. **Vandaar dat het noodzakelijk blijft om door een arts opgevolgd te blijven.**

Ongeveer 1 in 10 (10%) patiënten met CHH-een omkering

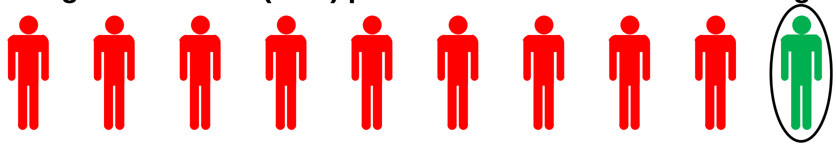

Kan een patient met CHH vruchtbaar worden?

Ja, **CHH is een behandelbare vorm van onvruchtbaarheid.** De meerderheid - niet iedereen - van de CHH patienten kan vruchtbaar worden met een **speciale hormonale behandeling**.

• dit kan ofwel onder vorm van hormonale inspuitingen, verschillende keren per week, ofwel door het dragen van een GnRH pomp (lijkt op de pomp bij diabetes patiënten).

• de vruchtbaarheidsbehandeling wordt best opgevolgd door een endocrinloog gespecialiseerd in voortplanting

Soms (maar niet altijd) kan de vruchtbaarheid pas bekomen worden via bijkomende technieken zoals IVF(in vitro fertilisatie)

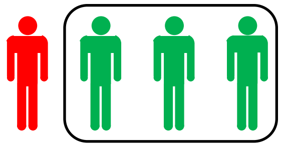

Vruchtbaarheid: zal bij 3 op de 4 patienten (75%) mogelijk zijn

Mannen**:** het kan soms 2 jaar duren alvorens de spermaproductie op gang komt

Vrouwen: sommige vrouwen kunnen al na enkele maanden vruchtbaar zijn

Heeft het niet behandelen van CHH nadelige gevolgen?

Testosterone en oestrogenen zijn geen levensnoodzakelijke hormonen,maar hun afwezgheid of tekort kan ernstige gevolgen hebben voor de **gezondheid, het sexueel functioneren en voor het algemeen welzijnsgevoel.**

<u>Bij mannen en vrouwen</u>

- Verhoogd risico op een **verlaagde botdensiteit (osteoporose):** dit betekent dat de botten brozer zijn en dat er een verhoogde kans op breuken bestaat. Als CHH niet behandeld wordt kan osteoporose op gelijk welke leeftijd optreden. Sommige patienten met CHH hebben zelfs een extra behandeling voor de osteoporose nodig.
- De lage hormoonspiegels geven een **verminderde sexuele functie en verlangen.**
- **Zonder behandeling kan de patient zich moe, lusteloos en depressief voelen.**

Zonder behandeling hebben mannen een verhoogd risico op metabole problemen zoals pre-diabetes of **diabetes. Dit risico kan verminderd worden door de behandeling niet te onderbreken.**

Wat je zeker moet weten:

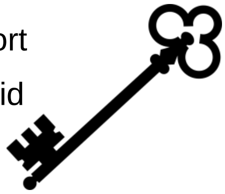

- CHH is een zeldzame aandoening veroorzaakt door een GnRH tekort
- CHH veroorzaakt een uitblijven van de puberteit en de vruchtbaarheid
- CHH verkort je levensduur niet
- De diagnose van CHH is moeilijk
- De diagnose wordt soms laat in de tienerjaren of pas bij jong volwassenen gesteld
- In tegenstelling tot andere zeldzame aandoeningen kan deze aandoening behandeld worden
- De behandeling is levenslang, met regelmatige opvolging bij de arts
- Bespreek met de arts de behandelingsmogelijkheden en zoek samen welke het beste bij jou past
- Stoppen van de behandeling kan je gezondheid schaden en je minder goed in je vel doen voelen
- Tijdens de aangepaste behandeling is vruchtbaarheid mogelijk
- Je kan CHH doorgeven aan je kinderen, daarom is genetisch advies aangeraden
- Voor sommige patienten is de CHH aandoening psychologisch moeilijk om dragen
- Je kan hulp en ondersteuning krijgen van je arts en van je lotgenoten

Handige referentie

http://www.nature.com/nrendo/journal/v11/n9/full/nrendo.2015.112.html

Handige websites

http://www.gnrhnetwork.eu/hhn_home/hhn-patients_familles_gnrh_deficiency_kallmann_syndrome/hhn-onlinesupportgroups.htm

https://www.rareconnect.org/en/community/kallmann-syndrome

https://www.facebook.com/KallmannSyndrome/

https://www.gezondheid.be

Ce que vous devez savoir sur les hypogonadismes hypogonadotropes congénitaux (HHC) et le syndrome de Kallmann (SK)

Qu'est ce que l'HHC ?

L'HHC est une maladie qui résulte souvent d'un déficit en GnRH (gonadotropin releasing hormone). Lorsque l'HHC est associé à une diminution de la perception des odeurs on parle de syndrome olfacto-génital ou syndrome syndrome de Kallmann (SK).

Quels sont les effets de la GnRH?

1. La GnRH est une neuro-hormone essentielle pour le développement sexuel, la puberté et la fertilité. La GnRH est libérée par l'hypothalamus, une petite glande située à la base du cerveau et qui contrôle toute une série de fonctions essentielles à l'organisme.
2. La GnRH agit directement sur l'hypophyse, une glande située juste en dessous de l'hypothalamus, de la taille d'un petit pois, en stimulant la sécrétion de deux hormones: la LH (hormone lutéinisante) et la FSH (hormone folliculo stimulante).

1. La LH et la FSH sont des hormones essentielles pour la puberté et la fertilité:

- Chez l'homme: La LH et la FSH stimulent la production de testostérone et de spermatozoïdes par les testicules.
- Chez la femme: la LH et la FSH stimulent la production ovarienne d'estradiol et de progesterone, deux hormones qui sont essentielles pour la puberté, les cycles menstruels et la fertilité.

Que se passe-t-il en l'absence de GnRH ?

Un déficit partiel ou complet en GnRH induit une diminution du taux de LH et de FSH. La résultante est une absence de puberté et une infertilité (voir figure ci-dessous). Ceci veut dire:

- Chez l'homme: 1) l'absence de croissance des testicules , 2) la non production de testostérone, et 3) l'incapacité à produire des spermatozoïdes.

- Chez la femme: 1) L'infertilité et l'absence d'ovulation - les follicules sont présents mais ne se développent pas et ne libérent pas d'ovocyte, 2) L'estradiol et la progestérone ne sont pas ou peu produites, et 3) Il n'y a pas de cycle menstruel (pas de règles).

Manifestations de l'absence de développement pubertaire chez les adolescents

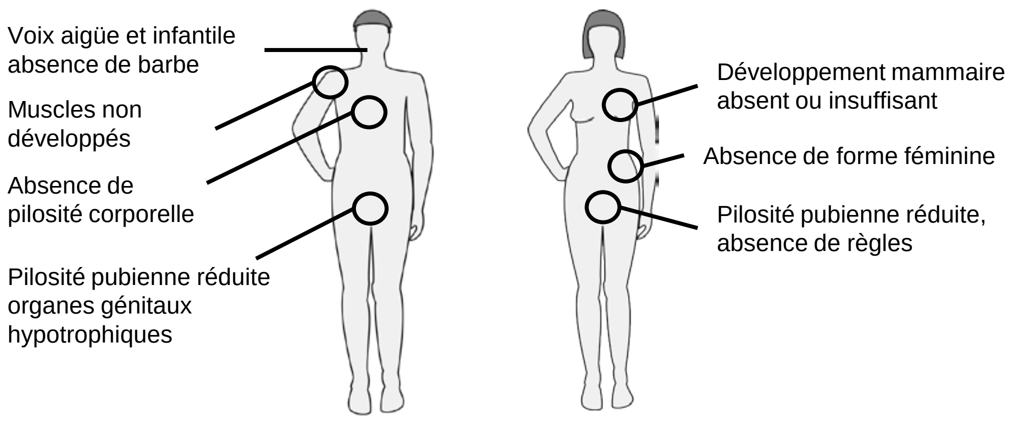

Des signes d'HHC peuvent être présent à la naissance (d'ou le terme congénital) mais le diagnostic est souvent fait chez l'adolescent ou chez le sujet jeune.

Qui est atteint ?

Aussi bien les hommes que les femmes. L'HHC est plus souvent diagnostiqué chez les hommes. La raison n'en est pas claire, mais il est possible que chez un nombre important de femmes atteintes d'HHC le diagnostic ne soit pas fait et qu'un simple traitement par pilule contraceptive soit proposé par le gynécologue « pour régulariser les règles ». Chez un certain nombre de patients le diagnostic ne sera pas fait, car ils n'auront jamais vu un spécialiste de cette maladie.

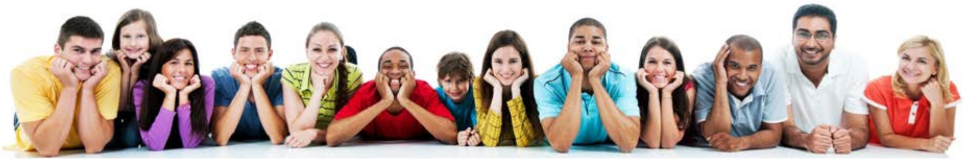

Combien de personnes en sont atteintes ?

L'HHC est une maladie rare, une estimation fiable est donc difficile. On pense que la fréquence de l'HHC est comprise entre 1/4'000 et 1/10'000 personnes. Ceci veut dire que le nombre total de patients HHC en Europe pourrait être de l'ordre de 74'000 et remplir un grand stade olympique.

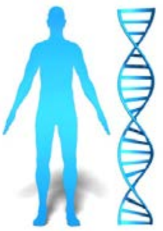

L'HHC est-il héréditaire ?

L'HHC est fréquemment héréditaire. Ceci veut dire qu'il peut être **transmis** à la descendance et que dans certaines familles plusieurs générations sont touchées. Dans certains cas il n'y a pas d'histoire familiale apparente. Une cause génétique peut actuellement être identifié dans près de la moitié des cas. Des recherches sont en cours pour découvrir les causes génétiques pour les autres patients. Dans de nombreux cas il est difficile de déterminer de façon fiable si un patient donné va transmettre la maladie à ses enfants.

Chez la moitié des patients HHC une cause génétique est connue, pas chez l'autre moitié

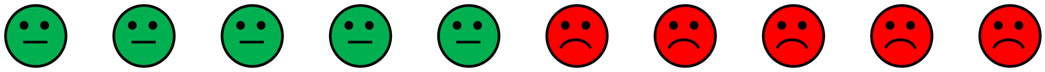

L'HHC et le SK sont génétiquement complexes car ils résultent parfois de la combinaison de mutations sur plusieurs gènes différents chez un patient donné. En dehors des cas provoqués par des mutations de *KAL1/ANOS1*, la prévision d'une transmission de la maladie aux enfants est difficile. Des recherches sont donc encore nécessaires pour mieux comprendre les bases génétiques de cette maladie et pour établir des tests diagnostiques encore plus fiables.

Comment le HHC est-il diagnostiqué ?

Le HHC est souvent difficile à diagnostiquer car la puberté normale s'étend sur une longue période. Il est donc parfois difficile de dire si une puberté est simplement retardée (mais sera normale), ou bien s'il s'agit d'un HHC qui nécessitera une prise en charge. Dans ces cas douteux un suivi est nécéssaire pour voir si la puberté s'achève normalement ou non. Il faut toujours rechercher une perte de l'odorat pour dépister un SK.

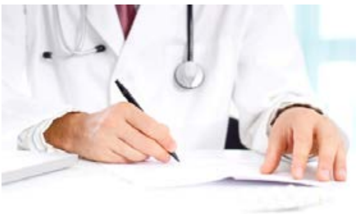

• **Garçons –** L'absence de signes de puberté vers 16 ans associé à des taux de testostérone et de LH et FSH bas indiquent un HHC.

• **Filles –** L'absence de développement des seins et de règles vers l'âge de 16-17 ans, accompagnés de taux sanguins bas d'estradiol et de LH et FSH indiquent un HHC.

• En cas de suspicion d'HHC d'autres examens doivent être pratiqués pour être sûrs qu'il n'y a pas d'autres maladies pouvant expliquer le tableau du patient. Il s'agit de tests hormonaux plus complets et d'examens d'imagerie (radios, scanner, IRM, échographies)

Existe-t-il des signes précoces ?
Chez de nombreux patients, l’HHC ne se révèle qu’à l’âge de la puberté.

En fait, chez certains garçons nouveau-nés, on peut observer des signes évoquant un HHC, comme un **micropénis ou des testicules non descendus (cryptorchidie)**.

Le micropenis peut être traité avec des hormones aussi bien chez le nourisson que pendant l’enfance.
Si les testicules restent mal positionnés ils doivent être descendus chirurgicalement de façon à préserver la fertilité.

Il ya des signes supplémentaires, non liés à l’HHC, qui apparaissent chez certains patients mais pas chez tous (voir figure à droite).

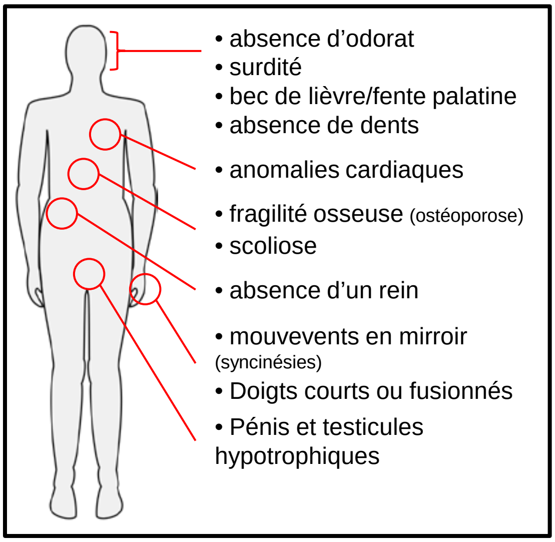

Une absence d’olfaction peut empêcher de détecter une odeur de gaz ou les mauvaises odeurs ainsi qu’altérer les capacités gustatives et diminuer la jouissance des repas.

Comment l’absence de puberté peut-elle affecter la qualité de vie d’un individu ?
Ne pas développer la puberté en même temps que les personnes d’âge comparable peut être très stressant et parfois même traumatisant. Il s’agit là du principal problème vécu par les patients atteints d’HHC. Cet impact peut être parfois sévère mais varie en fonction des sujets. La puberté est déjà une période difficile chez les adolescent ayant une puberté normale, ne pas développer sa puberté et être marginalisés par rapport aux groupes de même âge peut avoir des effets négatifs comme:

- perte de l’estime de soi , perte de confiance
- difficulté à interagir avec ses semblables
- anxiété, dépression
- altération de son image corporelle

Que peut on faire ?
Globalement, la majorité des symptômes de l’HHC peut être corrigée.
Un soutien psychologique peut parfois s’avérer utile.

1. **Parlez avec son médecin**- il est possible que le soignant ne se rende pas compte spontanément de votre souffrance. Mais, alerté, il peut vous conseiller et vous soutenir.
2. **Interagissez avec d’autres patients –** utilisez des groupes « on-line »(Facebook, RareConnect.org); des groupes de soutien entre patients peuvent être très utiles. Il s’agit de sites où les patients expriment librement et sans intermédiaire leur vrais problèmes et besoins. Ils peuvent ainsi mieux se rendre compte ce que veut dire vivre quotidiennement avec cette maladie et recevoir un soutien et des conseils pratiques d’autres patients.

Qu’est ce que vous devez faire pour être en bonne santé ?
Avoir un HHC ne diminue pas votre espérance de vie. Pour rester en bonne santé vous devez:

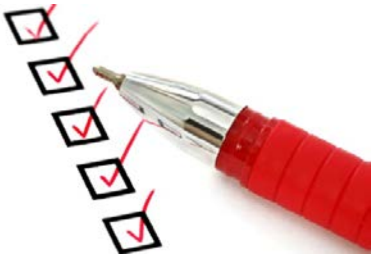

- Vous rendre régulièrement à vos consultations
- Prendre les traitements prescrits
- Avoir une alimentation saine
- Faire régulièrement de l’exercice physique
- Ne pas fumer

Quels sont les traitements disponibles pour les patients avec HHC ?

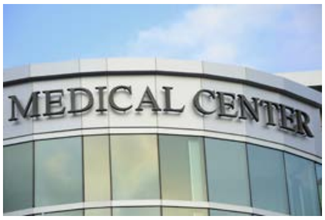

Peut-on guérir de l'HHC?

Actuellement **il n'y a pas de traitement curatif** pour l'HHC. Il est toujours très difficile de guérir d'une maladie congénitale. Des recherches sont en cours pour voir s'il est possible de restaurer la sécrétion de GnRH hypothalamique et/ou des gonadotrophines hypophysaires. Cette recherche est encore à un stade précoce mais on peut espérer qu'il sera possible d'aboutir dans un futur proche.

Y a-t-il des traitements disponibles pour l'HHC ?

Des médicaments sont disponibles pour développer des signes extérieurs de puberté comme le développement de la barbe chez les hommes ou des seins chez la femme. Il existe des thérapies plus complexes mais efficaces pour restaurer la fertilité chez la majorité des patients.

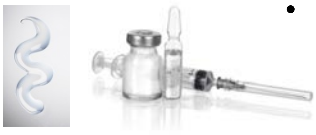

- Chez les hommes: **la testostérone** (injections, gélules ou gels cutanés) est le traitement le plus utilisé pour développer les signes pubertaires. La testostérone stimule la croissance staturale, rend la voix plus grave, fait pousser la barbe et le pénis, et augmente le désir et l'activité sexuels – mais elle n'a pas d'effet sur la fertilité.

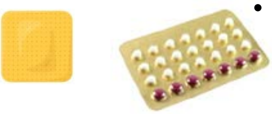

- Chez les femmes: Des pilules (mais aussi patchs ou gels) contenant des faibles doses d'œstrogènes permettent de développer les seins et la silhouette. En association avec la **progestérone** elles permettent de provoquer des règles (saignement menstruel).

Quand le traitement est débuté tôt dans l'adolescence, les doses sont augmentées progressivement. Ceci peut parfois être frustrant mais il s'agit d'optimiser le développpement des seins chez la femme et la croissance staturale dans les deux sexes.

Chez certains patients la maladie peut spontanément s'améliorer après le traitement, avec une restauration des sécrétions hormonales propres. On appelle ceci une forme réversible. Les mécanismes impliqués ne sont pas compris et on ne peut pas actuellement prédire quel patient va présenter une réversibilité. De plus, **la réversibilité n'est pas forcément durable .** Par conséquent même en cas de réversibilité **le suivi médical prolongé restera nécessaire.**

Près d'un patient atteint d'HHC sur 10 (10%) a une forme réversible

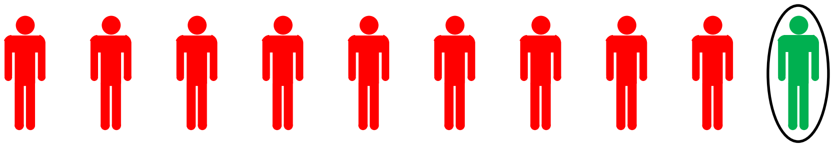

Est-ce qu'une personne atteint d'HHC peut devenir fertile ?

Oui, l'HHC est une forme curable d'infertilité**.** La majorité (mais pas la totalité) des patients HHC peut devenir fertile grâce à des **traitements hormonaux spécialisés.**

Fertilité: Globalement, 3 patients sur 4 (75%) deviennent fertiles

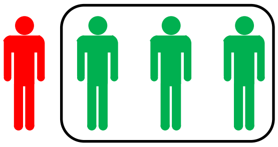

Hommes: il faut parfois être traité pendant 2 ans pour produire des spermatozoïdes

Femmes: très souvent on restaure la fertilité en quelques mois (ovulation)

- Les traitements spécialisés sont soit administrés sous forme d'injections sous-cutanées pluri-hebdomadaires (gonadotrophines) ou soit avec une pompe à GnRH portable.
- Le traitement de l'infertilité nécessite de consulter un spécialiste expert (**endocrinologue de la reproduction**)
- Parfois (moins fréquent) une procréation médicale, comme une fécondation in vitro (FIV) *est nécessaire*

Points-clé et résumé

Y a-t-il un risque si l'HHC n'est pas traité ?
Bien que les œstrogènes et la testostérone ne soient pas vitaux, leur absence ou leur déficit peuvent sérieusement impacter **la santé**, la **sexualité** et la **qualité de vie**.

<u>Chez les hommes et les femmes</u>

- Risque accru de fragilité osseuse avec **ostéoporose** – et risque de fractures. Si l'HHC n'est pas traité, l'ostéoporose peut apparaître à tout âge. Chez certains patients atteints d'HHC et d'ostéoporose, des traitements supplémentaires et spécifiques à visée osseuse sont parfois nécessaires.
- **Une diminution du désir et de l'activité** sexuelle résultent de l'insuffisance d'hormones sexuelles.
- **La fatigue, le mal être, et la dépression** sont fréquents chez les patients ayant arrêtés le traitement

Chez les hommes non traités il y a aussi un risque de troubles métaboliques, de pré-diabète ou de diabète.

Tous ces risques peuvent être diminués en suivant correctement le traitement.

Points-clé:

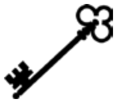

- l'HHC est une maladie rare causée par un déficit en GnRH et/ou en gonadotrophines
- L'HHC empêche le développement pubertaire normal et provoque l'infertilité
- L'HHC ne diminue pas l'espérance de vie
- L'HHC n'est pas toujours aisé à diagnostiquer
- La majorité des patients sont diagnostiqués tard dans l'adolescence ou comme jeunes adultes
- Contrairement à beaucoup de maladies rares, il y a des traitements efficaces
- Vous avez besoin d'un traitement médical à vie et un suivi médical au long cours
- Discutez avec votre médecin à des différentes options thérapeutiques pour choisir celle qui vous conviendra le mieux.
- Arrêter le traitement aura des conséquences négatives sur votre santé et votre bien-être
- La fertilité est possible chez la majorité des patients atteints d'HHC grâce à des traitements spécialisés
- Vous pouvez transmettre un HHC à votre descendance, un conseil génétique est donc recommandé
- L'HHC peut avoir des conséquences psychologiques importantes chez certains patients
- Vous pouvez trouver un soutien auprès de votre médecin et au sein de groupes de patients

Références utiles

http://www.nature.com/nrendo/journal/v11/n9/full/nrendo.2015.112.html

Sites web utiles

http://www.gnrhnetwork.eu/hhn_home/hhn-patients_familles_gnrh_deficiency_kallmann_syndrome/hhn-onlinesupportgroups.htm

https://www.rareconnect.org/en/community/kallmann-syndrome

https://www.facebook.com/KallmannSyndrome/

Deutsch

Was Sie über kongenitalen hypogonadotropen Hypogonadismus (CHH) & das Kallmann Syndrom (KS) wissen sollten

Was ist CHH?

CHH wird durch einen **GnRH (gonadotropin releasing hormone) Mangel** ausgelöst. Ist dieser mit einem gestörten Geruchsinn (Anosmie) kombiniert, nennt man es olfacto-genitales Syndrom oder auch Kallmann Syndrom (KS). CHH und KS werden ähnlich diagnostiziert und behandelt.

Wie wirkt GnRH?

GnRH ist ein essenzielles Hormon für die sexuelle Entwicklung, Pubertät und Fruchtbarkeit.

1. GnRH wird von spezifischen Zellen aus einem Bereich des Gehirns, dem Hypothalamus, ausgeschüttet.
2. GnRH stimuliert die Hypophyse (eine fast erbsengrosse Hirnanhangsdrüse) zur Freisetzung von zwei Hormonen: das luteinisierende Hormon (LH) und das follikelstimulierende Hormon (FSH).
3. LH & FSH sind wichtig für die Pubertät und die Funktion der Keimdrüsen (Fruchtbarkeit).

    • Bei Männern: LH & FSH stimulieren die Hoden zur Bildung von Testosteron und regen die Spermienbildung an.

    • Bei Frauen: LH & FSH stimulieren im Eierstock (Ovar) die Bildung der Hormone Oestrogen & Progesteron, sowie die Eireifung (Fruchtbarkeit).

Was passiert, wenn kein GnRH vorhanden ist?

Ist kein GnRH vorhanden, fehlen die Hormone LH und FSH. Dieser Mangel verursacht eine ausbleibende oder unvollständige Pubertätsentwicklung und kann zu Unfruchtbarkeit führen (siehe Abbildung unten). Dies bedeutet, dass:

- Bei Männern: 1) die Hoden nicht wachsen, 2) Testosteron nicht produziert wird, und 3) die Spermienbildung verhindert wird.
- Bei Frauen: 1) sich die Eibläschen nicht entwickeln, d.h. die Eier sind zwar präsent, wachsen aber nicht und werden nicht freigesetzt (Ovulation), 2) Östrogen und Progesteron nicht produziert werden, und 3) die Menstruation (Monatsblutung) ausbleibt

Zeichen für verzögerte Pubertät bei Jugendlichen und jungen Erwachsenen

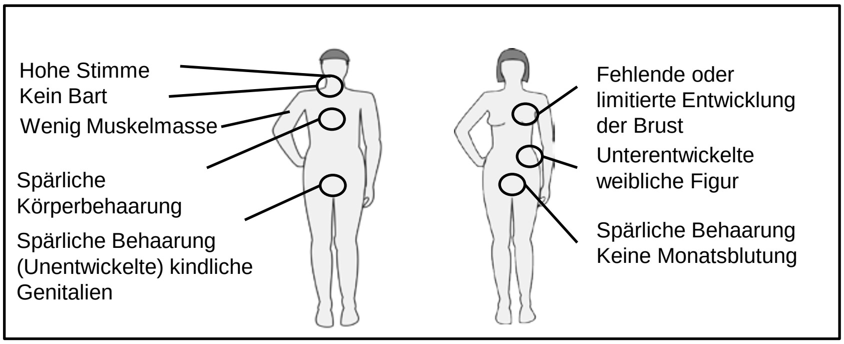

Der GnRH Mangel ist bereits bei Geburt vorhanden (daher auch angeboren (kongenital) genannt) aber die Diagnose wird oft erst bei Jungendlichen oder bei jungen Erwachsenen gestellt. **Wichtig zu wissen ist, dass diese Krankheit behandelbar ist. In den meisten Fällen ist eine lebenslange Hormonersatztherapie & Betreuung notwendig.**

2017: www.gnrhnetwork.eu / andrew.dwyer@chuv.ch

Wer ist von CHH betroffen und wie wird es diagnoziert?

Wer ist von einem CHH betroffen?

Männer und Frauen sind gleichermaßen betroffen. Allerdings wird CHH öfter bei Männern diagnostiziert. Der Grund dafür ist nicht klar. Es kann aber daran liegen, dass betroffene Frauen häufig die „Pille“ verschrieben bekommen und dadurch regelmässige Monatsblutungen aufweisen, ohne das der Verdacht auf eine zentrale Hormonstörung gestellt wird. Somit werden manche Patienten auch keinem Spezialisten für CHH / GnRH-Defizit vorgestellt.

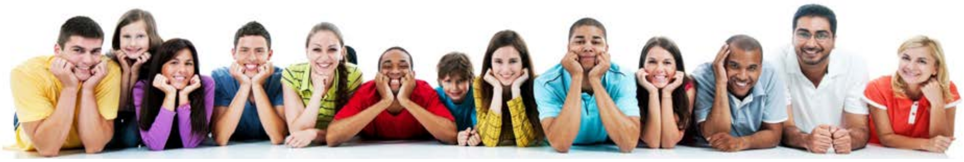

Wie viele Menschen sind betroffen?

CHH ist sehr selten, daher ist eine genaue Schätzung schwierig. Wir gehen davon aus dass CHH ungefähr bei einem von 4’000 bis 10’000 Menschen auftritt. Das bedeutet, dass die Gesamtheit der Patienten mit CHH in Europa (um die 74’000) das Olympische Stadium in Berlin füllen würde.

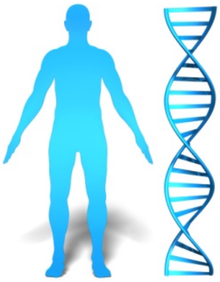

Ist CHH vererbbar ?

CHH kann vererbbar sein. Das bedeutet, dass CHH **von einer Generation an die andere weitergegeben werden kann**. In manchen Fällen gibt es keine offensichtliche Familiengeschichte bezüglich Erbleiden. Dennoch wird bei ca. 50% der Patienten eine genetische Ursache gefunden. In den meisten Fällen ist es schwierig, zuversichtlich und exakt vorherzusagen, ob ein einzelner Patient den CHH an seine Kinder weitervererben wird oder nicht. Über die genetischen Ursachen bei CHH wird weiterhin viel geforscht.

Die Hälfte der Patienten haben eine bekannte genetische Ursache, die anderen nicht.

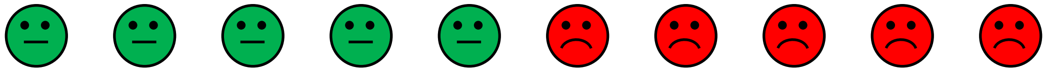

In manchen Fällen entsteht CHH erst durch die Kombination aus zwei oder mehreren Veränderungen (Mutationen) in verschiedenen Genen. Das macht es schwer herauszufinden, ob CHH an die Kinder weitervererbt werden kann. Für ein Gen, ANOS1 (KAL1), scheint eine Vorhersage einfach. Weitere Forschung ist notwendig um die Vorgänge besser zu verstehen und um die Diagnose zu erleichtern .

Wie wird CHH diagnostiziert?

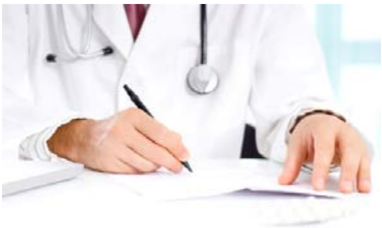

CHH ist schwer zu diagnostizieren. Die Pubertät verläuft über eine weite Zeitspanne. Dies macht es manchmal sehr schwer zu entscheiden, ob bei einem Menschen eine verzögerte aber normale Pubertät oder ein CHH vorliegt, und ob eine medizinische Behandlung nötig wird. Häufige Untersuchungen des Pubertätsverlaufes sind daher notwendig. Patienten sollten immer nach einem gestörten Geruchssinn gefragt werden.

Mögliche Zeichen für das Vorliegen eines CHH sind bei:

• **Jungs –** fehlende Pubertätsmerkmale im Alter von 16 Jahren, niedrige Blutwerte für Testosteron , LH & FSH .

• **Mädchen –** fehlende Entwicklung der Brust im Alter von 14-15 Jahren und ausbleibende Monatsblutungen im Alter von 16-17 Jahren, niedrigem Östrogen, LH & FSH im Blut.

• **Weitere Tests** sind manchmal notwendig, um sicher zu gehen dass keine anderen Gründe die erniedrigten Hormonspiegel verursachen. Diese Tests beinhalten Laboruntersuchungen und bildgebende Verfahren (Röntgen-, Kernspintomographieaufnahmen, Ultraschalluntersuchungen).

Gibt es frühe Zeichen?
Bei vielen Patienten fehlen Anzeichen auf das Vorliegen eines CHH vor dem Ausbleiben der Pubertät.

Manche Jungen weisen Auffälligkeiten auf wie einen **Mikropenis oder einen Hodenhochstand** (Kryptorchidismus).

Ein Mikropenis kann in der Kindheit mit einer Hormontherapie behandelt werden. Ein Hodenhochstand sollte im ersten Lebensjahr mittels Operation korrigiert werden, um die Hodenfunktion zu erhalten.

Es gibt weitere –auch nicht durch CHH bedingte- Anzeichen, die bei manchen aber nicht allen Patienten auftreten können.

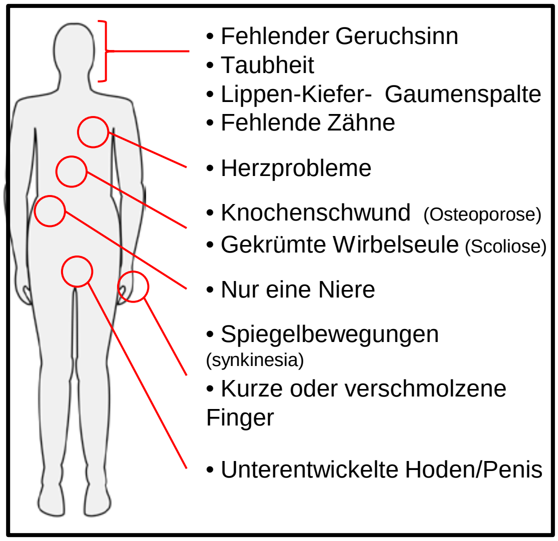

Welche Auswirkungen hat das Ausbleiben der Pubertät noch?
Die Unsicherheiten der Pubertät können für jeden Jugendlichen schwer zu bewältigen sein. Das Ausbleiben der Pubertät oder eine im Vergleich zu Altersgenossen andersartige Pubertätsabfolge kann aber auch als **psychischer Stress oder als traumatisierendes Ereignis** empfunden werden. Auch wenn das persönliche Empfinden individuell sehr unterschiedlich ist, kann dies das Hauptproblem für Patienten mit CHH sein und folgende Auswirken haben :

- Fehlende Selbstachtung, mangelndes Selbstvertrauen
- Besorgnis, Depression
- Schüchternheit, Schwierigkeit mit anderen zu kommunizieren
- Minderwertigkeitsgefühle
- Beziehungsprobleme

Die Störung des Geruchssinns kann das Geschmacksempfinden und den Nahrungsgenuss beeinträchtigen. Manche Gase, verdorbene Speisen oder auch Körpergerüche könnten schlecht wahrgenommen werden.

Was können sie machen?
Diese Probleme sind zu bewältigen. Psychologische Beratung und Therapie können hilfreich sein.

1. **Sprechen Sie über Ihre Sorgen mit Ihrem Ärzteteam** und erklären Sie diesem, was Sie empfinden oder ob Sie eine harte Zeit durchleben. Das Team wird Sie dabei unterstützen, die beste Lösung zu finden.
2. **Suchen Sie Kontakt zu anderen Patienten**– über Internet, in online-Gruppen (Facebook, www.RareConnect.org) oder in Selbsthilfegruppen. Dort können Patienten über die Probleme reden, die für sie wichtig sind. Andere Betroffene verstehen wie es ist, mit CHH zu leben und können nützlichen Rat und notwendige Unterstützung geben.

Was sollten sie machen, um gesund zu bleiben?
Ihre Lebenserwartung sollte durch CHH nicht verkürzt sein. Es gibt vieles was Sie tun können, um gesund zu bleiben :

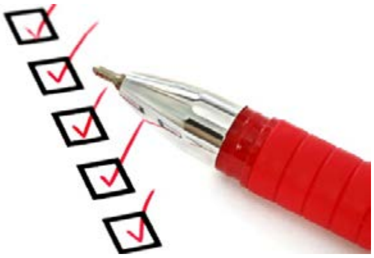

- Gehen Sie zu regelmäßigen Gesundheitsvorsorgen
- Nehmen Sie die verschriebenen Medikamente vorschriftsmäßig
- Ernähren Sie sich gesund
- Regelmäßige körperliche Bewegung ist wichtig
- Rauchen Sie nicht

2017: www.gnrhnetwork.eu / andrew.dwyer@chuv.ch

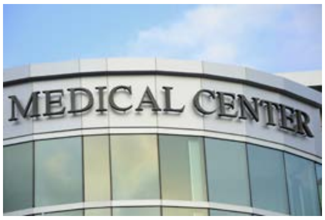

Kann man CHH heilen?
Im Moment gibt es **keine Heilung für CHH**. Es ist sehr schwer, eine genetisch bedingte Krankheit zu heilen. Laufende Forschungen versuchen herauszufinden, ob die Ausschüttung von GnRH durch den Hypothalamus wieder angeregt werden kann. Diese Forschung steht noch am Anfang, aber es gibt Hoffnung, dass dies in der Zukunft möglich sein wird.

Gibt es eine Behandlung für CHH?
Die Entwicklung der Pubertätsmerkmale, wie den Penis- und Bartwachstum bei Männern und die Brustentwicklung bei Frauen, **kann durch Hormonbehandlungen beeinflusst werden**

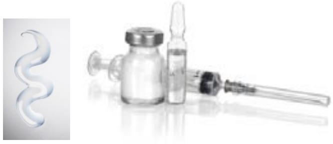

Bei Männern: **Testosteron** (in Form von Spritzen in den Muskel oder in Form von Gel oder Pflastern) ist die häufigste Behandlung. Die Therapie stimuliert das Körperlängenwachstum, eine tieferwerdende Stimme, Bartwachstum, Peniswachstum und Erektionen.

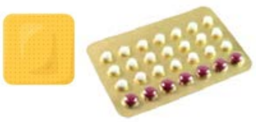

- Bei Frauen: schon eine geringe **Östrogendosis** (in Form von Pflastern oder Pillen) stimuliert das Längenwachstum und die weibliche Brust- und Figurentwicklung. Kombiniert mit Progesteron, induziert dieses Hormon die regelmäßigen Monatsblutungen.

Bei Beginn der Behandlung wird eine niedrige Dosis gewählt, die nur langsam erhöht wird. Dieses kann für diejenigen frustrierend sein, die auf eine schnelle Wirkung hoffen. Es ist aber wichtig, um ein maximales Körperwachstum (und Brustentwicklung bei Frauen) zu ermöglichen.

Für die **Fruchtbarkeitsbehandlung** gibt es weitere spezifische Methoden .

Bei manchen Leuten ist eine Behandlung des CHH nur vorübergehend notwendig, d.h. sie produzieren nach Beendigung der Hormontherapie einen ausreichenden GnRH Hormonspiegel. Das wird **„Reversal (Umkehr)"** genannt. Wir verstehen die Gründe dafür noch nicht und können es im Enzelfall nicht voraussehen. **Die Gonadenachse bleibt aber weiterhin störungsanfällig.** Daher ist es wichtig auch nach einem „Reversal" regelmäßige Untersuchungen durchführen zu lassen.

Ungefähr einer von 10 (10%) Patienten mit CHH haben eine Umkehr

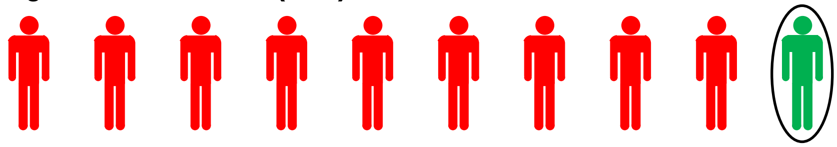

Kann eine Person mit CHH fruchtbar werden?
Ja, der **CHH ist eine behandelbare Form von Unfruchtbarkeit.** Für die Mehrheit - aber nicht für alle - Menschen mit CHH kann mit einer speziellen Hormontherapie der Kinderwusch erfüllt werden.

Fruchtbarkeit: Eine Behandlung ist bei etwa 3 von 4 Patienten (75%) erfolgreich

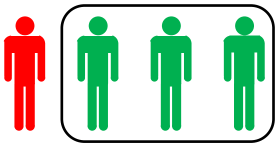

Männer: es kann bis zu zwei Jahre dauern um Sperma zu entwickeln
Frauen: in manchen Fällen ist ein Eisprung nach ein paar Monaten möglich.

- Die spezialisierte Fruchtbarkeitsbehandlung besteht entweder aus Hormon-Spritzen, die mehrere Male pro Woche notwendig sind, oder mittels einer tragbaren Pumpe (wie bei der Diabetesbehandlung) .
- Fruchtbarkeitsbehandlungen benötigen eine intensive Betreuung von erfahrenen Spezialisten (Reproduktionsendokrinologen)
- selten ist eine künstliche Befruchtung wie die **In-vitro-Fertilisation** (IVF) notwendig.

Welche Risiken bestehen bei Nichtbehandlung eines CHH ?
Während Testosteron und Östrogen keine lebenswichtigen Hormone sind, kann deren Fehlen jedoch einen ernsthaften Einfluss auf die **Gesundheit, sexuelle Funktion und Lebensqualität** haben**.**

Bei Männern & Frauen

- Grösseres Risiko für **Osteoporose („Knochenschwund“)** – die Knochendichte ist vermindert und es besteht somit ein erhöhtes Risiko, Knochenbrüche zu erleiden. Wenn CHH nicht behandelt wird, kann Osteoporose eine Person jeden beliebigen Alters treffen. Manche Menschen mit CHH brauchen daher eine zusätzliche Behandlung einer Osteoporose.
- **Verringerte sexuelle Funktion und Lust** sind das Ergebniss von erniedrigten Hormonspiegeln.
- **Müdigkeit, Hoffnungslosigkeit und Depression** können bei nichtbehandelten Patienten auftreten.

Männer ohne Behandlung haben ausserdem ein erhöhtes Risiko, Probleme wie das metabolische Syndrom oder einen Diabetes zu entwickeln. **Diese Risiken können durch die Behandlung vermindert werden.**

Schlüsselpunkte:

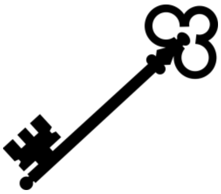

- CHH ist eine seltene Krankheit, verursacht durch einen Mangel an GnRH
- CHH verursacht eine ausbleibende oder unvollständige Pubertätsentwicklung und kann zu Unfruchtbarkeit führen
- CHH sollte Ihre Lebenserwartung nicht verkürzen
- CHH ist schwierig zu diagnostizieren
- Viele Patienten werden erst in den späten Pubertätsjahren oder als junge Erwachsene diagnostiziert.
- Anders als bei vielen seltenen Krankheiten ist eine Behandlung möglich
- Eine lebenslange Behandlung und regelmässige Arztbesuche sind notwendig
- Sprechen Sie mit Ihrem Arzt, um zu entscheiden, welche Behandlung für Sie persönlich am besten ist
- Eine Behandlung zu unterbrechen oder zu beenden kann negative Auswirkungen auf Ihre Gesundheit und Ihr Wohlbefinden haben.
- Fruchtbarkeitbehandlung ist in den meisten Fällen mit einer speziellen Hormontherapie möglich
- Sie könnten CHH an Ihre Kinder vererben, daher ist eine genetische Beratung empfohlen
- CHH ist für manche Menschen psychologisch schwer zu bewältigen
- Sie können Unterstützung bei Gesundheitsdienstleistern und Patientengruppen finden

Hilfreiche Referenzen
http://www.nature.com/nrendo/journal/v11/n9/full/nrendo.2015.112.html

Hilfreiche Websites
http://www.gnrhnetwork.eu/hhn_home/hhn-patients_familles_gnrh_deficiency_kallmann_syndrome/hhn-onlinesupportgroups.htm

https://www.rareconnect.org/en/community/kallmann-syndrome

https://www.facebook.com/KallmannSyndrome/

Ελληνικά

Τι χρειάζεται κάποιος να γνωρίζει για τον Συγγενή Υπογοναδοτροφικό Υπογοναδισμό (ΣΥΥ) & το Σύνδρομο Kallmann

Τι είναι ο ΣΥΥ?

Ο ΣΥΥ προκαλείται από αδυναμία έκκρισης της ορμόνης **GnRH (gonadotropin-releasing hormone**) από τον υποθάλαμο. Όταν συνδυάζεται με ανοσμία (έλλειψη οσφρητικής ικανότητας) περιγράφεται ως οσφρητικό – γεννητικό σύνδρομο η Σύνδρομο Kallmann. Η διάγνωση και θεραπεία για όλους τους ασθενείς είναι η ίδια.

Ποιος ο ρόλος της GnRH?

Η GnRH αποτελεί αναγκαία ορμόνη για την ομαλή σεξουαλική ανάπτυξη, ενήβωση και γονιμότητα.

1. Η GnRH εκκρίνεται από τον υποθάλαμο, ο οποίος είναι ένας μικρός αδένας στον εγκέφαλο που κατευθύνει ζωτικές λειτουργίες του ανθρώπινου σώματος.
2. Η GnRH δρα απευθείας στην υπόφυση (παραπλήσιος αδένας μικρού μεγέθους μέσα στον εγκέφαλο) και έτσι απευλεθερώνονται οι Γοναδοτροφίνες ορμόνες: Ωχρινοτρόπος Ορμόνη (LH) και Θυλακιοτρόπος Ορμόνη (FSH).
3. Τα επίπεδα των γοναδοτροφινών LH & FSH είναι σημαντικά για την έναρξη και ολοκλήρωση της εφηβείας καθώς και για την επίτευξη γονιμότητας
   - στους άνδρες: οι ορμόνες LH & FSH συμβάλλουν στην παραγωγή της Τεστοστερόνης και του σπέρματος από τους όρχεις
   - στις γυναίκες : οι ορμόνες LH & FSH δια της διέγερσης των ωοθηκών συμβάλλουν στην παραγωγή οιστρογόνων και προγεστερόνης που είναι σημαντικές ορμόνες για την γονιμότητα

Τι συμβαίνει σε περίπτωση ανεπάρκειας GnRH?

Η ολική η μερική ανεπάρκεια GnRH οδηγεί στην αδυναμία έκκρισης LH & FSH. Το τελικό αποτέλεσμα είναι η αποτυχία ενήβωσης και η υπογονιμότητα (Δες το σχεδιάγραμμα παρακάτω). Επομένως:

- <u>Στους άρρενες:</u> 1) οι όρχεις δεν μεγεθύνονται 2) η τεστοστερόνη δεν παράγεται και 3) δεν δημιουργείται σπέρμα.
- <u>Στις γυναίκες:</u> 1) τα ωάρια δεν αναπτύσσονται – παρά το γεγονός ότι είναι παράγονται, δεν μεγεθύνονται ούτε και απελευθερώνονται (ωορρηξία) 2) δεν παράγονται οιστρογόνα και προγεστερόνη 3) δεν εμφανίζεται έμμηνος ρύση.

Σημεία αποτυχούς ενήβωσης σε εφήβους και νεαρούς ενήλικες

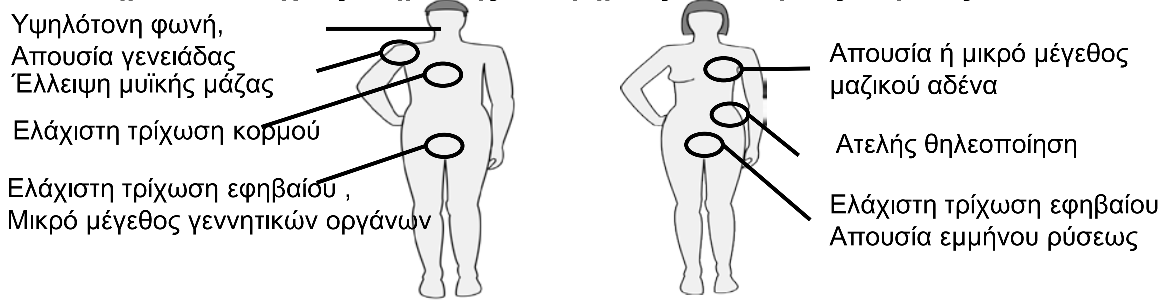

Τα συγκεκριμένα προβλήματα σχεδόν πάντα εμφανίζονται από τη γέννηση (ονομάζονται Συγγενή) και η διάγνωση τίθεται κατά την εφηβεία η στα πρώτα χρόνια της ενήλικου ζωής. **Η συγκεκριμένη πάθηση είναι θεραπεύσιμη και στις πλείστες των περιπτώσεων απαιτείται χρόνια ορμονική θεραπεία.**

Ποιοι πάσχουν από ΣΥΥ και πως τίθεται η διάγνωση ?

Ποιοι πάσχουν από ΣΥΥ?

Τόσο οι άνδρες όσο και οι γυναίκες πάσχουν με ΣΥΥ. Ο ΣΥΥ για αδιευκρίνιστους λόγους διαγιγνώσκεται πιο συχνά στους άνδρες. Ίσως επειδή οι γυναίκες που πάσχουν καταφεύγουν στο γυναικολόγο και παίρνουν συνταγή για αντισυλληπτικά δισκία έτσι ώστε να έχουν κανονική περίοδο, χωρίς να έχουν μια καθαρή διάγνωση. Μερικοί ασθενείς ποτέ δεν έχουν την ακριβή διάγνωση και ποτέ δεν έχουν αξιολογηθεί από ιατρό ενδοκρινολόγο, ειδικό για την έλλειψη GnRH.

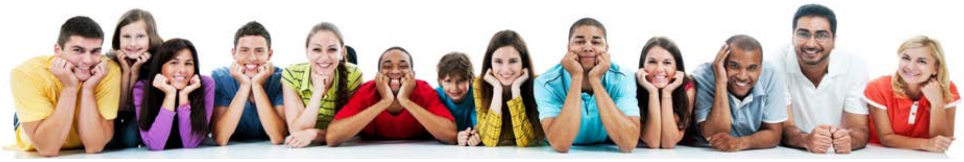

Πόσα άτομα πάσχουν?

Ο ΣΥΥ είναι σπάνια πάθηση έτσι ώστε η ακριβής συχνότητα να είναι δύσκολη. Πιστεύουμε ότι ο ΣΥΥ παρατηρείται σε 1: 4,000 – 10,000 πληθυσμού. Αυτό σημαίνει ότι ο συνολικός αριθμός ασθενών με ΣΥΥ στην Ευρώπη (περίπου 74,000) θα γέμιζε το Ολυμπιακό στάδιο του Βερολίνου.

Ο ΣΥΥ κληρονομείται?

Ο ΣΥΥ μπορεί να είναι κληρονομικός. Αυτό σημαίνει αυτό σημαίνει ότι μπορεί να μεταφέρεται από γενεά σε γενεά και μέσα στην ίδια οικογένεια. Σε μερικές περιπτώσεις δεν υπάρχει εμφανές οικογενειακό ιστορικό. Σε περίπου 50% των περιστατικών αναγνωρίζεται γενετική αιτία. Η έρευνα προχωρεί και αναμένεται να προσδιορίσει τις γενετικές αιτίες της ΣΥΥ. Σε πολλές περιπτώσεις είναι δύσκολο για τον επαγγελματία υγείας με ακρίβεια να προβλέψει αν ένας ασθενής με ΣΥΥ μπορεί να μεταφέρει την πάθηση στα παιδιά του.

Μισοί από τους ασθενείς έχουν γνωστή γενετική αιτία, όχι όμως οι άλλοι μισοί

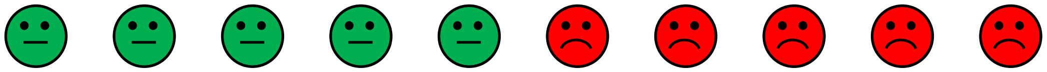

Ο ΣΥΥ επιπλέκεται επειδή σε μερικές περιπτώσεις μπορεί να οφείλεται σε συνδυασμό δύο η περισσοτέρων αλλαγών (μεταλλάξεις) σε διαφορετικά γονίδια. Αυτό δυσχεραίνει την πρόβλεψη αν θα μεταφερθεί στα παιδιά. Χρειάζεται περισσότερη μελέτη για καλλίτερη κατανόηση της διάγνωσης.

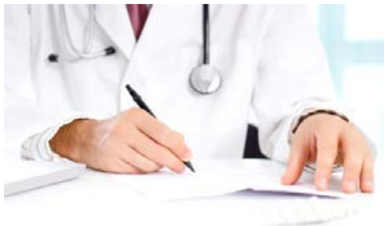

Πως γίνεται η διάγνωση του ΣΥΥ?

Ο ΣΥΥ δύσκολα διαγιγνώσκεται. Υπάρχει ένα εύρος στη χρονική εμφάνιση της εφηβείας που καθιστά δύσκολη την απόφαση αν είναι μια απλή φυσιολογική καθυστέρηση η ΣΥΥ που να απαιτεί θεραπεία. Η περιοδική παρακολούθηση της προόδου στην εφηβεία είναι αναγκαία.

• **Αγόρια** – απουσία σημείων ενήβωσης στην ηλικία των 16 χρόνων και χαμηλά επίπεδα τεστοστερόνης, LH & FSH συνηγορούν για ΣΥΥ.

• **Κορίτσια –** Απουσία μαζικού αδένα σε ηλικία 14-15 χρόνων και απουσία εμμήνου ρήσεως στα 16-17 χρόνια με χαμηλά επίπεδα οιστρογόνων, LH & FSH συνηγορούν για ΣΥΥ.

• Επιπρόσθετα τεστ (αιματολογικές εξετάσεις, απεικονιστικές μελέτες ,X-rays, ultrasound & MRI) πρέπει να γίνουν για αποκλεισμό άλλων καταστάσεων που προκαλούν παρόμοια εικόνα.

Πως μπορεί ο ΣΥΥ να επηρεάσει την υγεία και τον τρόπο ζωής ?

Υπάρχουν έγκαιρα σημεία να κοιτάξεις?
Στους περισσότερους δεν υπάρχουν σημεία του ΣΥΥ πριν από την αποτυχία στην ενήβωση.

Μερικά άρρενα βρέφη μπορεί να έχουν μκροπέος η κρυψορχία (μη κάθοδος των όρχεων ενδεικτικά ΣΥΥ.

Το μικροπέος μπορεί να θεραπευθεί στη βρεφική και παιδική ηλικία με ορμονική θεραπεία. Η κρυψορχία πρέπει να διορθωθεί χειρουργικά στον πρώτο χρόνο της ζωής για εξασφάλιση γονιμότητας.

Σε μερικούς ασθενείς παρατηρούνται και άλλα σημεία τα οποία δεν οφείλονται στον ΣΥΥ.

.

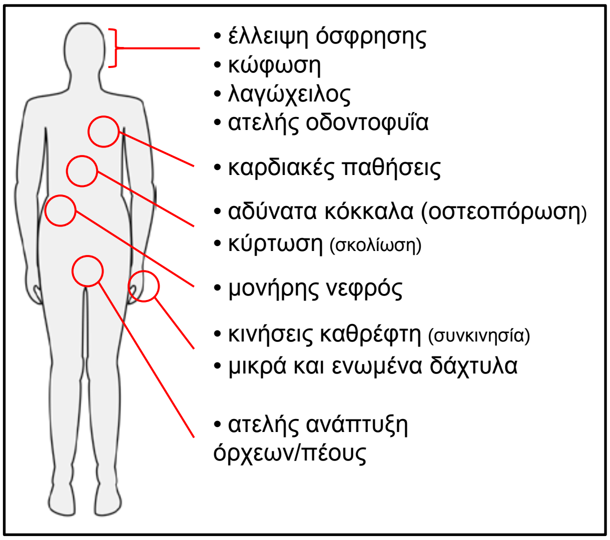

Πως η απουσία εφηβείας επηρεάζει κάποιο άτομο?
Η μη είσοδος στην εφηβεία μπορεί να είναι στρεσογόνος και μερικές φορές τραυματική. Αυτό αποτελεί ένα μείζον θέμα στους ασθενείς με ΣΥΥ. Η επίδραση μπορεί να είναι σοβαρή σε μερικές περιπτώσεις αν και διαφοροποιείται από άτομο σε άτομο. Τα χρόνια της εφηβείας είναι δύσκολα για τους εφήβους οι οποίοι εισέρχονται φυσιολογικά στην ήβη, αλλά η μη εμφάνιση εφηβείας και διαφοροποίηση από τους συνομήλικους μπορεί να οδηγήσει σε διάφορα θέματα όπως:

- χαμηλή αυτοεκτίμηση και αυτοπεποίθηση l
- ντροπαλότητα και χαμηλή κοινωνικότητα
- άγχος, κατάθλιψη
- χαμηλή αυτό εικόνα

Τι θα μπορούσε να γίνει?
Αυτά τα προβλήματα αντιμετωπίζονται. Η ψυχολογική στήριξη και θεραπεία είναι χρήσιμες.

1. **Μίλησε με το δικό σου επαγγελματία υγείας –** Μπορεί να μην αναγνωρίζει ότι περνάς μια δύσκολη φάση και μπορεί να σε βοηθήσει να βρεις τη κατάλληλη συμβουλευτική και στήριξη.
2. **Κάνε σύνδεση με άλλους ασθενείς –** Η χρήση ομάδων από το διαδίκτυο (π.χ Facebook) και η διαπροσωπική επαφή βοηθούν πολύ. Αυτά είναι μέρη όπου οι ασθενείς μπορούν να μιλήσουν για τα θέματα που τους απασχολούν. Άλλοι ασθενείς αντιλαμβάνονται πως είναι να ζεις καθημερινά με ΣΥΥ και είναι ικανοί να παρέχουν πρακτικές συμβουλές και στήριξη.

Τι πρέπει να κάνεις για να είσαι υγιής?
Ο ΣΥΥ δεν μικραίνει τη ζωή. Αυτά είναι τα πράγματα που πρέπει να κάνεις για να είσαι υγιής.

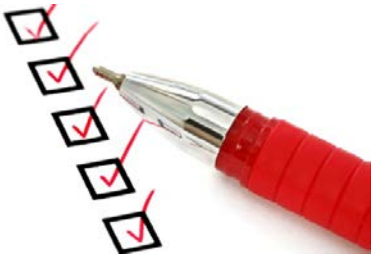

- Τακτική ιατρική παρακολούθηση
- Να παίρνεις τακτικά τη θεραπεία σου
- Υγιεινή διατροφή
- Κανονική φυσική άσκηση
- Αποφυγή καπνίσματος

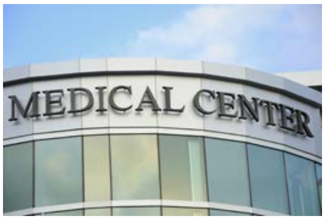

Είναι ο ΣΥΥ θεραπεύσιμος?

Για την ώρα **δεν υπάρχει θεραπεία για τον ΣΥΥ**. Είναι δύσκολο να θεραπεύσει μια γενετική πάθηση. Η έρευνα προχωρεί έτσι ώστε να είναι σε θέση να αποκαταστήσει την παραγωγή GnRH production από τον υποθάλαμο. Η έρευνα είναι σε πολύ αρχικά στάδια αλλά μας δίνει ελπίδες για το μέλλον.

Υπάρχουν διαθέσιμες θεραπείες για τον ΣΥΥ?

Υπάρχουν θεραπείες έτσι ώστε να αναπτύσσονται τα εμφανή σημεία της εφηβείας – όπως η τρίχωση του προσώπου στους άνδρες και η ανάπτυξη των μαστών στις γυναίκες. Υπάρχουν επίσης ειδικές ορμονικές θεραπείες για αντιμετώπιση της υπογονιμότητας.

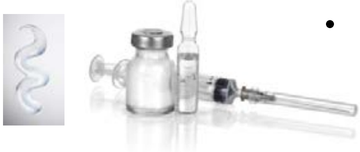

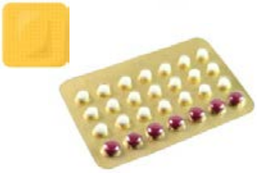

- Στους άνδρες: **Τεστοστερόνη** (ζελέ για επάλειψη στο δέρμα η ενέσεις) είναι η πιο συνήθης θεραπεία για την ανάπτυξη των σημείων της εφηβείας. Η θεραπεία οδηγεί σε αύξηση, αλλαγή της φωνής, γενειάδα, αύξηση του πέους και της σεξουαλικής λειτουργίας – αλλά όχι γονιμότητα.
- Στις γυναίκες: Χαμηλή δόση οιστρογόνων (ταμπλέτες) οδηγεί σε αύξηση, ανάπτυξη των μαστών και θηλεοποίηση του σώματος. Ο συνδυασμός με προγεστερόνη προκαλεί την εμφάνιση και την περιοδικότητα της εμμήνου ρήσεως.

Μετά την έναρξη της θεραπείας **η αύξηση της δόσης προσαρμόζεται σταδιακά** . Αυτό μπορεί να ενοχλεί μερικούς ασθενείς αλλά είναι σημαντικό για καλλίτερα αποτελέσματα.

Κάποιοι ασθενείς με ΣΥΥ θεραπεύονται μετά από την αγωγή και είναι σε θέση να παράγουν φυσιολογικά επίπεδα ορμονών. Το φαινόμενο αυτό λέγεται αναστροφή η οποία δεν είναι προβλέψιμη, δεν γνωρίζουμε γιατί συμβαίνει, **δεν διαρκεί για πάντα, έτσι ώστε να είναι σημαντική η τακτική ιατρική παρακολούθηση από επαγγελματία υγείας.**

Περίπου 1:10 (10%) από τους ασθενείς με ΣΥΥ έχει αναστροφή

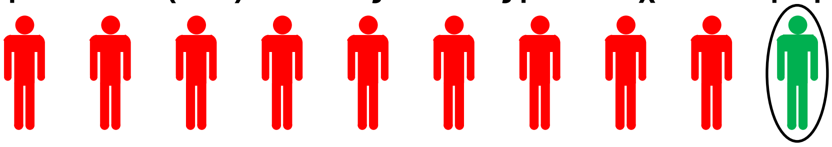

Μπορεί ένας ασθενής με ΣΥΥ να είναι γόνιμος?

Ναι, **η ΣΥΥ είναι θεραπεύσιμη μορφή υπογονιμότητας.** Η πλειονότητα – όχι όλη - ασθενών με ΣΥΥ μπορεί να γίνουν γόνιμοι **με ειδική ορμονοθεραπεία.**

Γονιμότητα: Γενικά, σε 3 από τους 4 ασθενείς (75%) επιτυγχάνεται

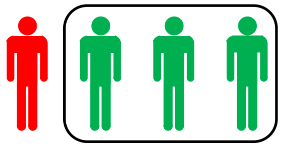

Άνδρες: Μπορεί να πάρει μέχρι και 2 χρόνια η ικανοποιητική παραγωγή σπέρματος
Γυναίκες: Σε μερικές περιπτώσεις η γονιμότητα επιτυγχάνεται σε μερικούς μήνες

- Εξειδικευμένες θεραπείες μπορεί να είναι ορμονικές ενέσεις μερικές φορές την εβδομάδα η εφαρμογή αντλίας GnRH (όπως αυτές που χρησιμοποιούνται στον Διαβήτη)..
- Η θεραπεία της γονιμότητας γίνεται σε εξειδικευμένα κέντρα (**ενδοκρινολόγοι αναπαραγωγής**)
- Κάποτε (όχι πάντα) απαιτείται υποβοηθούμενη αναπαραγωγή όπως εξωσωματική γονιμοποίηση (IVF).

Υπάρχουν κίνδυνοι αν δεν χορηγηθεί θεραπεία σε άτομα με ΣΥΥ?

Ενώ η Τεστοστερόνη και τα Οιστρογόνα είναι ζωτικής σημασίας ορμόνες, δεν είναι απαραίτητες για τη ζωή. Η απουσία τους όμως έχει σοβαρές επιπτώσεις στην **υγεία, σεξουαλική λειτουργία και ποιότητα ζωής.**

Σε άνδρες και γυναίκες

- Κίνδυνος για χαμηλή **οστική πυκνότητα (οστεοπόρωση)** – αδύνατα και εύθραυστα κόκκαλα, κίνδυνος για κατάγματα. Αν ο ΣΥΥ αφεθεί χωρίς θεραπεία, η οστεοπόρωση μπορεί να επηρεάσει τον ασθενή σε οποιαδήποτε ηλικία. Μερικοί ασθενείς με ΣΥΥ χρειάζονται επιπρόσθετη θεραπεία για την οστεοπόρωση
- **Μειωμένη σεξουαλική επιθυμία και λειτουργία** είναι αποτέλεσμα ορμονικής έλλειψης
- **Καταβολή, κατάπτωση και κατάθλιψη** είναι συχνά σε ασθενείς με ΣΥΥ που δεν παίρνουν θεραπεία

Οι άνδρες χωρίς θεραπεία κινδυνεύουν να αναπτύξουν μεταβολικά νοσήματα όπως Σακχαρώδη Διαβήτη. **Οι κίνδυνοι αυτοί μειώνονται με την τήρηση της θεραπευτικής αγωγής.**

Σημεία Κλειδιά:

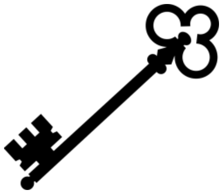

- Ο ΣΥΥ είναι μια σπάνια πάθηση που οφείλεται στην έλλειψη GnRH
- Ο ΣΥΥ οδηγεί σε απουσία ενήβωσης και στειρότητα
- Ο ΣΥΥ δεν επηρεάζει το προσδόκιμο επιβίωσης
- Ο ΣΥΥ είναι δύσκολο να διαγνωστεί
- Αρκετοί πάσχοντες δεν διαγιγνώσκονται πριν από την εφηβεία η τη νεαρά ενήλικο ζωή
- Σε αντίθεση με άλλες σπάνιες παθήσεις, υπάρχουν διαθέσιμες θεραπείες
- Χρειάζεσαι θεραπεία εφόρου ζωής και τακτικές ιατρικές επισκέψεις
- Μίλησε με το γιατρό σου για τις θεραπευτικές επιλογές για να αποφασίσετε την καλλίτερη για σένα θεραπεία
- Η διακοπή της θεραπείας έχει αρνητικές επιπτώσεις στην υγεία σου και στο αίσθημα ευεξίας
- Η γονιμότητα είναι δυνατή σε πολλές περιπτώσεις με ειδική ορμονοθεραπεία
- Μπορείς να μεταφέρεις την πάθηση στα παιδιά σου έτσι ώστε η γενετική συμβουλή να είναι απαραίτητη
- Ο ΣΥΥ είναι ψυχολογικά δύσκολος για κάποια άτομα
- Βρίσκεις στήριξη από τους επαγγελματίες υγείας και τιε ομάδες ασθενών

Βοηθητικές πηγές

http://www.nature.com/nrendo/journal/v11/n9/full/nrendo.2015.112.html

Βοηθητικές ιστοσελίδες

http://www.gnrhnetwork.eu/hhn_home/hhn-patients_familles_gnrh_deficiency_kallmann_syndrome/hhn-onlinesupportgroups.htm

https://www.rareconnect.org/en/community/kallmann-syndrome

https://www.facebook.com/KallmannSyndrome/

עברית

כל מה שעליך לדעת אודות היפוגונדיזם משני מולד (CHH) ועל תסמונת קלמן (KS)

מה זה CHH?
היפוגונדיזם משני מולד נגרם ע"י מחסור ב GnRH (הורמון משחרר גונדוטרופין). כאשר בנוסף יש פגיעה בחוש הריח (Anosmia), מדובר אז בתסמונת OLFACTO-GENITAL או סינדרום קלמן.
היפוגונדיזם משני מולד ותסמונת קלמן מאובחנים ומטופלים באותה הדרך.

מה עושה GnRH?
GnRH הוא הורמון החיוני עבור ההתפתחות המינית, גיל ההתבגרות והפוריות.
GnRHמשתחרר מההיפותלמוס, אזור קטן במוח השולט על מספר פונקציות של הגוף.
GnRH פועל באופן ישיר על בלוטת יותרת המוח (בלוטה קרובה להיפותלמוס שגודלה כגודל אפונה) וגורם לה לשחרר שני הורמונים: **LH**(הורמון "הצהבה") ו- **FSH** (הורמון "מגרה זקיק").
שני ההורמונים, LH-ו FSHחשובים מאוד לגיל ההתבגרות ולפוריות-
<u>בגברים</u>: ההורמונים מגרים את האשכים לייצר טסטוסטרון וזרע.
<u>בנשים</u>: ההורמונים מגרים את השחלות לייצר אסטרוגן ופרוגסטרון. חשובים לפוריות.

מה קורה כאשר אין GnRH?
חוסר חלקי או חוסר מלא ב- GnRH מתבטא בהעדר או ברמה נמוכה מאוד של LH ו- FSH. הנ"ל מתבטא בחוסר התבגרות מינית ופוריות (להלן תמונה). זה אומר ש:
<u>בגברים</u>:
האשכים אינם גדלים.
אין ייצור של טסטוסטרון בכמויות תקינות.
אין התפתחות של זרע.
<u>בנשים</u>:
אין התפתחות של הביציות, למרות שהן קיימות. הן אינן גדלות ולא משתחררות (ביוץ).
אין ייצור של אסטרוגן ושל פרוגסטרון.
אין תקופות וסת (לא מתרחש דימום).

סימנים להעדר התפתחות מינית בבני נוער ובבוגרים צעירים

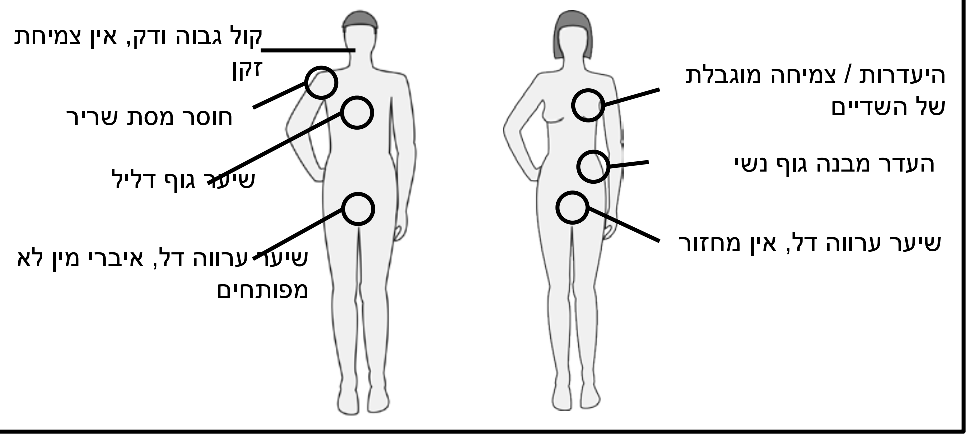

הבעיות האלה ברוב הזמן קיימות בלידה (לכן הסינדרום נקרא מולד)\ אולם האבחנה היא בד"כ בשנות הנעורים המוקדמות כאשר ההתבגרות המינית לא מתחילה.
חשוב לדעת – ניתן לטפל במצב זה.
ברוב המקרים ישנו צורך במתן תחליף הורמונלי לאורך החיים ובמעקב מתמשך.

2017: www.gnrhnetwork.eu / elih@bgu.ac.il

מי חולה ב CHH?
גם גברים וגם נשים יכולים להיות חולים במחלה. CHHמאובחן יותר בגברים. הסיבה לכך אינה ברורה.
ייתכן והסיבה לכך היא שנשים מבקרות אצל הגניקולוג ומקבלות מרשם לגלולות על מנת לקבל מחזורי וסת סדירים.
עם זאת, עדיין הן יכולות שלא לקבל את האבחנה הנכונה.
חלק מהחולים לעתים אינם מקבלים את הדיאגנוזה המדויקת וייתכן כי לעולם לא יפגשו מומחה לבעיית חוסר GnRH.

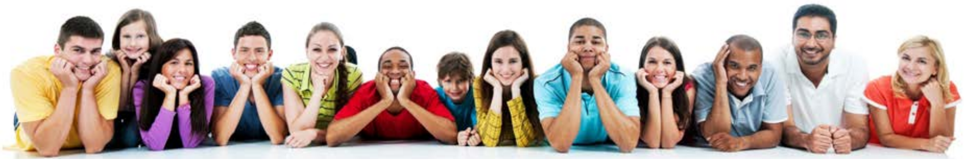

כמה חולים ישנם?
CHHהוא מצב נדיר כך שקשה להעריך באופן מדויק.
אנו מאמינים כי שכיחות התופעה היא 1 ל 4,000 – 10,000 אנשים. זה אומר שמספר האנשים עם CHH באירופה הוא בערך כ- 74000 (לדוגמא אצטדיון אולימפי מלא בברלין).

במחצית מהחולים ידועה הסיבה הגנטית, במחציתם הסיבה אינה ידועה

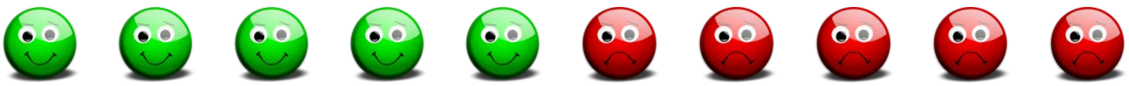

CHH עוברת בתורשה
זאת אומרת שאפשר להעביר במהלך הדורות בתוך המשפחות. ברוב המקרים, אין היסטוריה משפחתית.
סיבה גנטית יכולה להיות מזוהה בערך ב 50 אחוז מהזמן.
המחקרים הולכים ומתקדמים ואומנם עדיין יש מה לגלות אודות הסיבות הגנטיות הגורמות ל CHH.
CHH בחלק מהמקרים יכול להיגרם ע"י שילוב של שתיים או יותר מוטציות בגנים שונים. מצב כזה מקשה מאוד לנבא האם ה CHHיעבור לילדים. ברוב המקרים, לרופאים יהיה קשה לנבא האם חולה מסוים יעביר CHHלילדיו. בגן ANOS1(KAL1) ניתן לנבא בקלות.
יש צורך ביותר מחקר והבנה טובה יותר על מנת לשפר את הדיאגנוזה.

כיצד ניתן לאבחן CHH?
קשה לאבחן CHH. ישנו טווח רחב של התבגרות מינית נורמלית. לכן, מאוד קשה להחליט אם ההתבגרות המינית מתעכבת (אבל נורמלית) או שמדובר ב CHH ויש צורך בהתערבות רפואית.
יש צורך במעקב שגרתי על מנת לקבוע את התקדמות ההתבגרות המינית: חובה תמיד לשאול את החולים אודות ליקוי בחוש הריח.
<u>להלן סימנים המצביעים על היפוגונדיזם משני מולד:</u>
בנים: העדר סימני התבגרות מינית עד גיל 16 עם רמות נמוכות של טסטוסטרון, LH ו- FSH– מהווים אינדיקציה ל CHH.
בנות: חוסר התפתחות חזה עד גיל 14-15 וללא תקן' מחזור וסת (דימום) עד גיל 16-17, עם רמות נמוכות של אסטרוגן, LH ו – FSH, מצבעים על CHH.
בדיקות אחרות חייבות להתבצע על מנת לשלול מצבים אחרים שגורמים לרמות נמוכות של הורמונים.
בדיקות אלה כוללות בדיקות דם והדמיות (x-ray, אולטראסאונד ו- MRI).

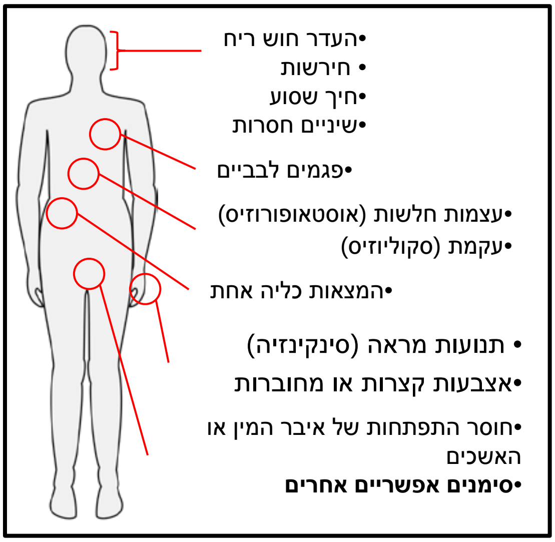

האם ישנם סימנים מקדימים שאפשר לחפש?

לרוב האנשים אין סימנים של CHH לפני חוסר ההתבגרות המינית.

חלק מהתינוקות הזכרים מראים סימנים שיכולים להצביע על CHH כגון – מיקרופניס (איבר קטן) או אשכים תמירים.

ניתן לטפל במיקרופניס בגיל הינקות / הילדות ע"י מתן טיפול הורמונלי.

אשכים תמירים ניתן לתקן ע"י ניתוח בשנה הראשונה לחייו של הילד על מנת למנוע בעיות של אי פוריות בעתיד.

ישנם סימנים אחרים של   CHHשמופיעים בחלק מהחולים, אבל לא בכולם.

כיצד העדר התבגרות מינית יכול לפגוע ?

חוסר התבגרות מינית יכול להיות מלחיץ מאוד לעתים ולפעמים אף טראומתי. זהו נושא משמעותי מאוד עבור חולים ב CHH.

התוצאה עשויה להיות מאוד חמורה מאוד בחלק מהמקרים, אם זאת ישנו שוני בין אדם לאדם.

שנות ההתבגרות יכולות להיות קשות מאוד עבור בני נוער שמתחילים את ההתבגרות המינית בקצב נורמלי, אולם חוסר התבגרות מינית, וההישארות מאחור מיתר בני הגיל יכולה ליצור מס' מצבים:

* הערכה עצמית נמוכה, בטחון עצמי נמוך.

* ביישנות, קושי באינטראקציה עם בני אותו הגיל

* חרדה, דכאון

• דימוי גופני נמוך

תחושת ריח לא תקינה עלולה לפגוע גם בתחושת הטעם וההנאה מהמזון. חולים עלולים שלא לחוש בריח גז או בריחן של מזון מקולקל. אחרים עלולים להיות מודאגים מריח גופם

מה אתה יכול לעשות?

ניתן להתגבר על כל הנ"ל. ייעוץ פסיכולוגי וטיפול יכולים להיות מאוד יעילים.

שוחח עם הרופא שלך – הוא עשוי להבין שאתה עובר תקופה קשה. הוא יכול לייעץ לך ולתמוך בך.

צור קשר עם חולים אחרים: שימוש בקבוצות אונליין כגון פייסבוק ותמיכה פנים מול פנים יכולים להיות מאוד יעילים. בקבוצות אלה החולים מדברים על נושאים שחשובים להם, על ההתמודדות היומיומית ויכולים לספק כלי ייעוץ ותמיכה מאוד יעיל.

מה עליך לעשות על מנת להיות בריא?

עצם היותך חולה ב CHHאינו מקצר את חייך.

<u>ישנם מספר דברים שאתה יכול לעשות על מנת להישאר בריא:</u>

להקפיד על מעקב רפואי

לקחת את הטיפול שנרשם לך

להקפיד על מזון בריא

להתאמן באופן שגרתי

לא לעשן.

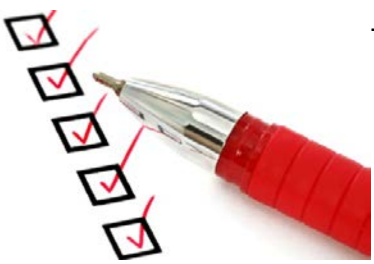

מהם הטיפולים הזמינים עבור חולי CHH?

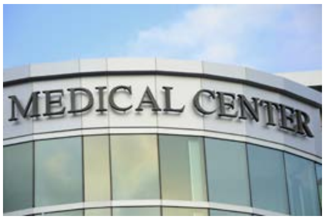

האם CHH היא מחלה שניתן לרפא?

נכון להיום, אין ריפוי ל CHH. קשה מאוד לרפא הפרעה מולדת.
ישנו מחקר המנסה לבדוק האם אפשר לשחזר ייצור של GnRH מההיפותלמוס. מחקר זה עדיין נמצא בשלביו הראשונים, אולם ישנה תקווה כי הדבר יהיה אפשרי בעתיד.

האם ישנם טיפולים זמינים עבור חולי CHH?

ישנם טיפולים זמינים שמטרתם להביא להתפתחות סימני התבגרות מינית, כגון: צמיחת זקן בגברים וצמיחת שדיים בנשים.
ישנם גם טיפולים הורמונליים מיוחדים שאמורים להביא להתפתחות הפוריות ברוב המקרים.
בגברים: טסטוסטרון (בג'ל למריחה על העור או בזריקות) הוא הטיפול היותר שכיח על מנת להביא לסימנים של התבגרות מינית.
הטיפול משרה גדילה, התעבות של הקול, צמיחת זקן, גדילה של איבר המין, דחף ותפקוד מיניים – אולם לא פוריות.
בנשים: מינון נמוך של אסטרוגן (גלולות או מדבקות) משרה גדילה ומביא לצמיחה של השדיים, להיווצרות מבנה גוף נשי, בשילוב עם פרוגסטרון יתרחשו גם מחזורי וסת (דימומים), אולם לא לפוריות.

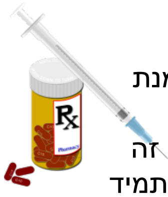

התאמת מינון הטיפול היא הדרגתית. מצב זה יכול להיות מייאש לחלק מהאנשים אבל הוא חשוב על מנת להביא למקסימום את הגדילה (ואת התפתחות השדיים בנשים).
חלק מהחולים ב CHH מתאוששים לאחר הטיפול ויכולים לייצר בעצמם רמות הורמונים תקינות. מצב זה נקרא "מהפך". הסיבות לכך אינן ברורות ולא ניתן לדעת למי מהחולים יהיה מהפך. התאוששות זו לא תמיד נמשכת, לכן חשוב מאוד להיות במעקב צמוד של הרופא המטפל.

1 מכל 10 חולים (10%) יחווה מהפך

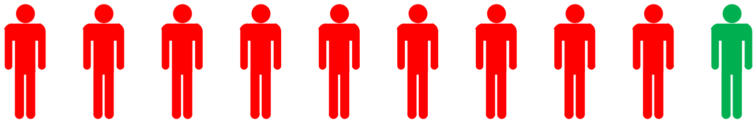

האם חולה ב CHHיכול להיות פורה?
כן. ברוב החולים (אך לא בכולם) ניתן להשרות פוריות– חולים יכולים להיות פוריים ע"י קבלת טיפול הורמונלי מתאים.
ישנן זריקות הורמונליות הניתנות מספר פעמים בשבוע או משאבת GnRH (כמו לחולי סוכרת).
טיפול בפוריות יש לקבל מרופא המומחה בבעיות פוריות (אנדוקרינולוג המומחה בבעיות פוריות).
לפעמים ישנו צורך באמצעים נוספים כגון הפרייה חוץ גופית (IVF).

פוריות: 3 מתוך 4 חולים (75%) הינם פוריים

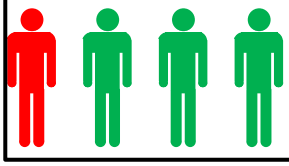

גברים: יכול לקחת כשנתיים להביא לידי התפתחות זרע.
נשים: בחלק מן המקרים הפוריות מושגת תוך מס' חודשים.

2017: www.gnrhnetwork.eu / elih@bgu.ac.il

מהם הסיכונים אם לא מטפלים ב CHH?

אומנם טסטוסטרון ואסטרוגן אינם הורמונים חיוניים לחיים, אולם חסרונם או העדרם יכול להשפיע באופן מהותי על הבריאות, התפקוד המיני ואיכות החיים.

<u>בגברים ובנשים:</u>

הסיכון לצפיפות עצם נמוכה (אוסטופורוזיס)עולה – המשמעות היא שהעצמות הינן חלשות וישנו סיכון מוגבר לשברים. אם לא מטפלים ב CHH, האוסטאופורוזיס יכולה לפגוע בכל גיל. לחלק מהאנשים עם CHH יש צורך בטיפול נוסף באוסטאופורוזיס.

חשק ותפקוד מיני נמוכים - הינם תוצאה של רמות הורמונליות נמוכות.

עייפות, דכאון – הינן תופעות נפוצות בקרב פציינטים שלא מקבלים טיפול.

גברים שאינם מקבלים את הטיפול הינם בסיכון גבוה יותר לפתח בעיות מטבוליות כגון: פרה-סוכרת או סוכרת.

סיכונים אלה פחותים כאשר מקבלים את הטיפול.

<u>נקודות מפתח</u>

- CHH הינו מצב נדיר הנגרם ע"י מחסור ב GnRH.
- CHH מתבטא בהעדר התבגרות מינית ופוריות.
- CHH אינה מחלה המקצרת את תוחלת החיים.
- קשה לאבחן CHH.
- הרבה חולים אינם מאובחנים עד שנות ההתבגרות המאוחרות או הבגרות המוקדמת.
- להבדיל ממחלות נדירות אחרות, ישנו טיפול זמין למחלה.
- ישנו צורך בטיפול לאורך החיים ובביקורים קבועים אצל הרופא המטפל.
- דבר עם הרופא שלך לגבי אופציות טיפוליות על מנת להחליט מה הכי טוב בשבילך.
- עצירת הטיפול מביאה להשלכות על מצבך הבריאותי ועל ההרגשה שלך.
- פוריות הינה אפשרית ברוב המקרים עם תמיכה הורמונלית מיוחדת.
- אתה יכול להעביר CHH לילדיך, לכן ייעוץ גנטי מומלץ מאוד.
- CHH יכולה להיות קשה נפשית לחלק מהאנשים.
- תוכל לקבל תמיכה מהרופא שלך ומקבוצות חולים.

מידע נוסף

http://www.nature.com/nrendo/journal/v11/n9/full/nrendo.2015.112.html

אתרי אינטרנט

http://www.gnrhnetwork.eu/hhn_home/hhn-patients_familles_gnrh_deficiency_kallmann_syndrome/hhn-onlinesupportgroups.htm

https://www.rareconnect.org/en/community/kallmann-syndrome

https://www.facebook.com/KallmannSyndrome/

(Magyar)

Amit a kongenitális hipogonadotrop hipogonadizmusról (CHH) és a Kallmann szindrómáról (KS) tudni kell

Mit jelent a CHH?

A CHH-t a **gonadotropin releasing hormon (GnRH) hiánya** okozza. Mikor ez a szaglóképesség elvesztésével (anozmia) is együtt jár, olfakto-genitális vagy Kallmann szindrómáról (KS) beszélünk. A fenti kórképek diagnózisa és kezelése nem különbözik.

Mi a GnRH élettani szerepe?

A GnRH a nemi fejlődés, a pubertás és a szaporodási képesség szempontjából fontos hormon.

1. A GnRH termelődési helye az a hipotalamusz nevű agyterület, amely számos testi funkció működését szabályozza.
2. A GnRH közvetlenül hatva az agyalapi mirigyre (cseresznye méretű mirigy a koponyaalapon) serkenti a luteinizáló hormon (LH) és a follikulus stimuláló hormon (FSH) termelődését.
3. Az LH és az FSH fontos szerepet játszanak a pubertásban és a szaporodásban
   • Férfiakban: Az LH és az FSH fokozza a here tesztoszteron és spermium termelését

   • Nőkben: Az LH és az FSH fokozza a petefészek ösztrogén és progeszteron hormonjainak termelését. E két hormon fontos szerepet játszik a termékenységben.

Mi történik GnRH hiányában?

A GnRH teljes vagy részleges hiányában megszűnik vagy csupán nagyon alacsony szinten zajlik az LH és az FSH termelése. **Ez a nemi érés hiányát és meddőséget** okoz (ld. az alábbi ábrát). Ilyen esetben:

- Férfiakban: 1) Nem következik be a here méretbeli növekedése, 2) nem termelődik tesztoszteron hormon normális mennyiségben és 3) nem képződik spermium sem.
- Nőkben: 1) Bár petesejtet tartalmazó tüszők jelen vannak a petefészkekben, azok nem indulnak fejlődésnek és nem következik be a tüszők érése és megrepedése (az ovuláció), 2) nem képződik ösztrogén és progeszteron hormon, és 3) elmaradnak a menstruációs ciklusok (havi vérzés).

A pubertás elmaradásának jelei kamasz korban és fiatal felnőttben

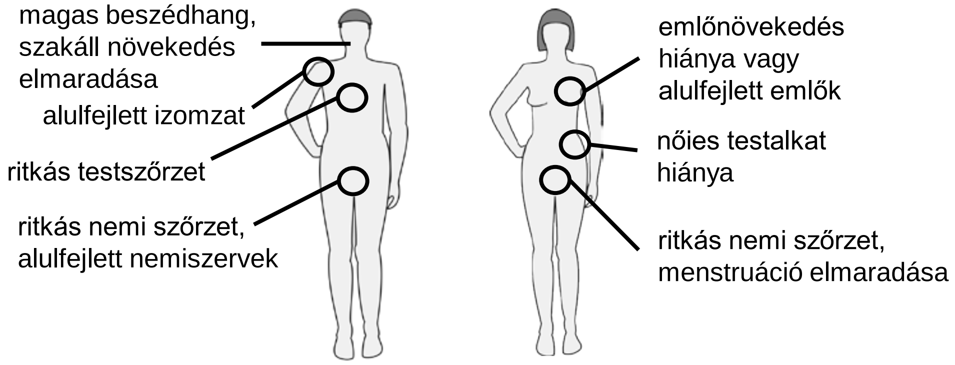

Mindezen problémák már születéskor fennállnak („kongenitálisak”), de a diagnózis gyakran csak tinédzser vagy felnőtt korban születik meg, mikor a nemi érés hiánya nyilvánvalóvá válik. **A legfontosabb tudnivaló, hogy ez az állapot kezelhető. A legtöbb esetben a kezelés élethosszig tartó hormon pótlást és utógondozást jelent.**

2017: www.gnrhnetwork.eu / andrew.dwyer@chuv.ch

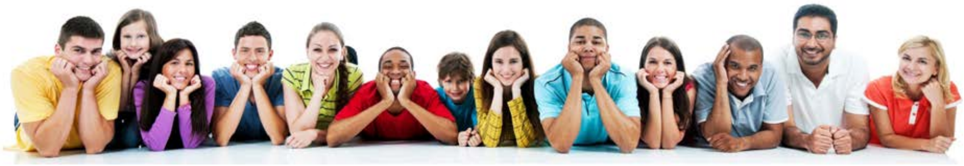

Kiket érint a CHH?

Mind férfiakat, mind nőket. A CHH kórkép férfiakban gyakrabban kerül felismerésre, melynek oka nem egyértelmű. Lehetséges, hogy számos érintett nő a menstruációs ciklus felépítése céljából nőgyógyászhoz fordul és fogamzásgátló tabletta felírását kéri, anélkül, hogy egyértelmű diagnózis születne meg. Egyes betegek esetében a pontos diagnózis soha nem születik meg és így nem történik meg a kapcsolatfelvétel szakorvossal sem.

Hány embert érint a CHH?

Mivel a CHH ritka betegség, előfordulási gyakoriságát nehéz pontosan megbecsülni. Ez 1:4000-1:10000 lehet, ami annyit jelent, hogy csak Európából a CHH-ban szenvedő betegek megtöltenék a 74000 főt befogadó Berlini Olimpiai Stadiont.

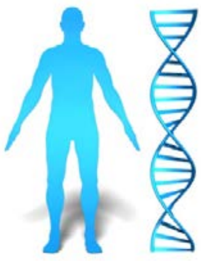

Öröklődik-e a CHH?

A CHH lehet örökletes, vagyis **átörökíthető** számos generáción át is a családon belül. Számos esetben azonban nincs más ismert beteg a családfában. Genetikai okot nagyjából az esetek felében sikerül kimutatni. A jelenleg folyó kutatások egyik célja a CHH genetikai hátterének jobb megértése. Általában nehéz a kezelőorvosnak azt megjósolnia, hogy a CHH-t okozó gént/géneket a beteg tovább örökíti-e az utódainak. Ez alól jelenleg csak az *ANOS1* (*KAL1*) gén képez kivételt.

A betegek felében sikerül feltárni genetikai okot, míg másik felében nem.

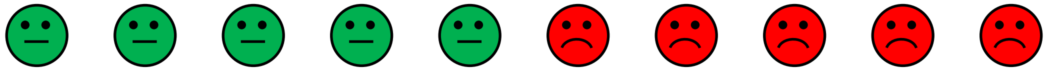

A CHH keletkezésének megértését tovább nehezíti, hogy bizonyos esetekben kettő vagy akár több, különböző génekben bekövetkezett változás (mutáció) együttesen felelős a kórképért. **Az ilyen CHH esetek különleges kihívást jelentenek az örökletesség megítélésében.** A CHH hátterének megértése és a diagnózis javítása további intenzív kutatást tesz majd szükségessé.

Miképp diagnosztizálható a CHH?

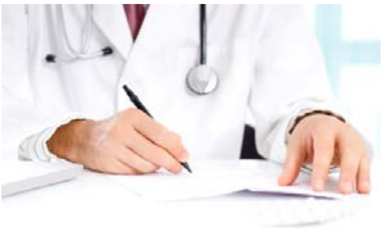

A CHH diagnózis felállítása nehéz. A pubertás ideje normálisan is változó. Emiatt nehéz lehet megítélni, hogy kései (de normális) pubertásról van-e szó, vagy szakorvosi kezelést igénylő CHH-ról. A pubertás lefolyásának nyomonkövetésére rendszeres orvosi ellenőrzés ajánlott. A szaglóérzék meglétére fontos mindig rákérdezni. CHH-ra utaló jelek lehetnek az alábbiak:

• **fiúk –** még nincs jele a pubertásnak 16 éves korban sem, és a szérum tesztoszteron, LH és FSH szintjei alacsonyak.

• **lányok –** még nem jelentkezik emlőfejlődés 14-15 éves korban sem, és nincs menstruáció (havi vérzés) 16-17 éves korban, alacsony szérum ösztrogén, LH és FSH szintek mellett.

• **További vizsgálatok (rtg, ultrahang, MRI)** is szükségesek lehetnek annak biztos megítéléséhez, hogy az alacsony hormon szintek nem egyéb betegség következményei.

Van-e olyan korai betegség tünet, amit keresni kell?
Sok esetben a CHH-nak nincsen semmilyen előjele kamaszkort megelőzően.

Egyes fiúgyermekekben a CHH előjele lehet a **mikropénisz vagy a le nem szállt here** (kriptorhizmus).

A mikropénisz gyermekkorban megkezdett hormonpótlással jól kezelhető. A le nem szállt here sebészi megoldást igényel még az első életévben, ami fontos a jövőbeli termékenység megőrzéséhez.

Egyes betegekben a CHH-tól független, további eltérések is megjelenhetnek.

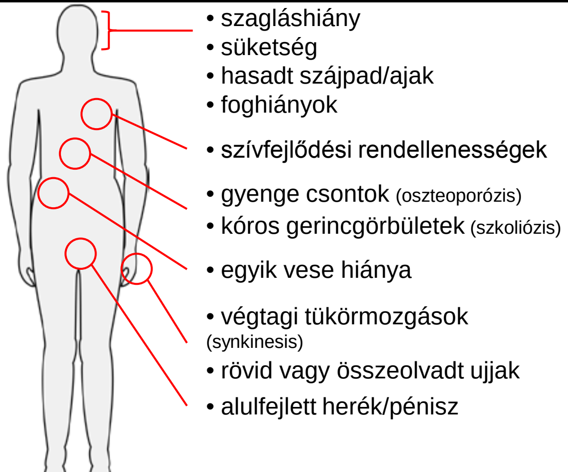

További lehetséges betegség tünetek

Hogyan hat az érintettekre a pubertás hiánya?
A pubertás elmaradása **gyakorta jár jelentős stresszel és lelki sérüléssel.** Ez a CHH-ban szenvedő betegek egy részénél jelent problémát. A pszichés következmények esetileg változó súlyosságúak. A tinédzser évek normál serdülőkben is nehézséggel járnak. A pubertás elmaradása viszont további olyan következményeket is maga után vonhat, mint az

- alacsony önbecsülés, csekély önbizalom
- félénkség, nehézségek az emberi kapcsolatokban
- szorongás, depresszió
- test miatti szégyenérzet

A szaglási rendellenesség károsan befolyásolja az ízek érzékelését és élvezetét. Megszűnik a gázok (pl. romló étel szagának) észlelése, egyesek saját vélt testszaguk miatt aggódnak.

Mi a teendő?
Összeségében a fenti következmények jól kezelhetőek. A lelki tanácsadás és a gyógyszeres terápia együttesen hatékony segítséget nyújtanak.

1. **A beteg beszéljen őszintén kezelőorvosával** – aki elképzelhető, hogy enélkül nincs is tudatában az állapot lelki következményeivel. A kezelőorvos segít a megfelelő tanácsadás és támogatás megtalálásában.
2. **A CHH-ban szenvedő lépjen kapcsolatba más betegekkel** – internetes csoportok (Facebook, RareConnect.org) és a személyes támogatói csoportok óriási segítséget jelenthetnek. Ezeken a fórumokon minden fontos probléma megbeszélésére lehetőség nyílik. Más hasonló betegek értik meg a legjobban, mit jelent naponta együtt élni CHH-val és hasznos gyakorlati tanácsokat, támogatást tudnak nyújtani.

Mi egyebet tehet még egy CHH-ban szenvedő beteg egészsége érdekében?
A CHH nem rövidíti meg az életet. Számos dolog tehető az egészség megőrzésére. A beteg

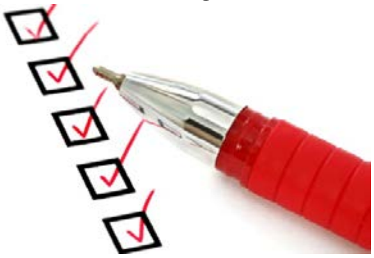

- tartson fenn rendszeres kapcsolatot kezelőorvosával,
- pontosan tartsa be a szakorvos által előírt kezeléseket
- táplálkozzon egészségesen
- végezzen rendszeres testmozgást
- ne dohányozzon

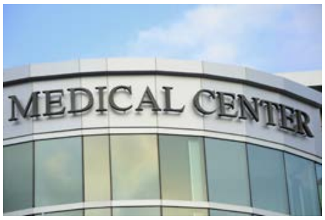

Gyógyítható-e a CHH?

Jelenleg, **oki kezelés CHH-ra nem áll rendelkezésre**. Nagyon nehéz egy veleszületett (genetikai) rendellenesség meggyógyítása. Kutatások folynak arra nézve, hogy miként lehet a hipotalamusz GnRH termelését visszaállítani. Ez a kutatás azonban még korai szakaszában van, és csak hosszabb távon kínál gyógyulási esélyt.

Kezelhető-e jelenleg a CHH?

Igen, létezik hatékony kezelés a pubertás olyan külső jegyeinek előidézésére, mint a szakáll növekedése férfiakban vagy az emlők növelése nőkben. Sőt, az esetek többségében speciális hormonkezeléssel a termékenység is kialakítható.

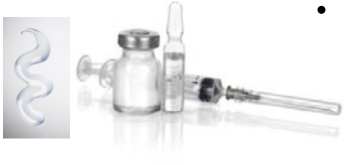

- férfiakban: a **tesztoszteron** hormon tapasz vagy injekció formájában történő pótlásával idézhetőek elő a pubertás jegyei. A kezelés növekedést, a hang mélyülését, a szakáll és a pénisz növekedését, és a libidó (nemi vágy) kialakítását idézi elő, de a termékenységet nem állítja helyre.

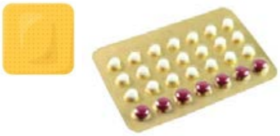

- nőkben: alacsony dózisban adott ösztrogén hormon (tabletta vagy tapasz formájában) növekedést okoz, és segíti az emlők fejlődését, a libidó (nemi vágy) és a nőies testalkat kialakulását. Progeszteron kezeléssel kombinálva szabályos menstruációs ciklusok (havi vérzés) alakíthatóak ki, de a termékenység nem áll helyre.

A kezelés megkezdésekor a **gyógyszeradag csak fokozatosan növelhető**. Ez frusztrációt okozhat mindazokban, akik gyors eredményben reménykednek. Elsőrendű cél azonban a maximális testnövekedést (és nőkben, emlőfejlődés) elérése.

Kevés CHH beteg a kezelés hatására meggyógyulhat, és képes lesz normális szintű hormon termelésre („reversal" jelenség). A jelenség okai nem ismertek és a bekövetkezte nem jósolható meg előre. **A „reversal" nem is mindig tartós. Ezért fontos, hogy a betegség lefolyását a kezelőorvos folyamatosan nyomon követhesse.**

Minden tízből egy (10%) CHH beteg mutat gyógyulást

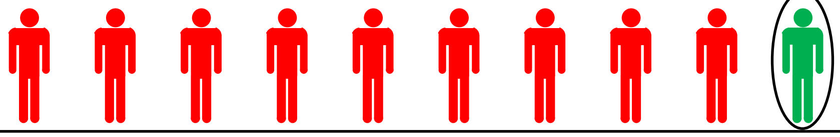

Lehet-e termékeny az, aki CHH betegségben szenved?

Igen, a CHH a meddőség kezelhető formája. A CHH betegek többsége, bár nem minden beteg termékeny lesz a **speciális hormonkezelés mellett.**

- A speciális kezelés heti többszöri hormon injekciókból vagy hordozható GnRH pumpa használatából áll (utóbbi a cukorbetegek inzulinpumpájához hasonló).
- A terméketlenség kezelése tapasztalt specialistát (**reproduktív endokrinológust**) igényel.
- Néha (nem mindig) szükség lehet művi megtermékenyítésre, mint az *in vitro* fertilizáció (IVF).

Termékenység: Összességében 4-ből 3 beteg (75%) esetén érhető el

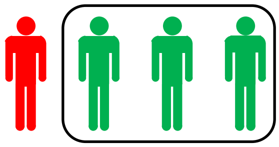

férfiakban: a spermium termeléshez 2 éves kezelés lehet szükséges
nőkben: esetenként termékenység érhető el már néhány hónapos kezeléssel is

Van-e annak kockázata, ha a CHH-t nem kezelik?
Noha a tesztoszteron és az ösztrogén nem létfontosságú hormonok, hiányuk súlyosan befolyásolhatja az **egészséget, a nemi működéseket és az életminőséget.**

<u>Férfiakban és nőkben</u>
- fokozott a kockázat az **alacsony csontsűrűségre (oszteoporózisra)** – ez azt jelenti, hogy a csontok gyengébbek és a csonttörés esélye megnövekszik. A CHH kezelése nélkül az oszteoporózis bármely életkorban jelentkezhet. Egyes CHH-ban szenvedő betegek külön kezelést igényelnek oszteoporózis ellen.
- **a csökkent nemi működés és vágy** az alacsony hormonszintek következményei
- **a fáradékonyság, levertség, depresszió** gyakori tünetek nem kezelt betegekben.

A nem-kezelt férfiak kockázata anyagcsere betegségekre, pre-diabéteszre és cukorbetegségre fokozott. Ezek a **kockázatok kezeléssel csökkenthetőek.**

Főbb pontok:

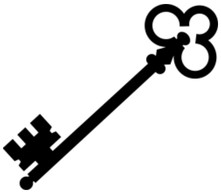

- A CHH olyan ritka kórkép, melyet a GnRH hiánya okoz
- A CHH a pubertás hiányával és terméketlenséggel jár
- A CHH <u>nem</u> csökkenti a várható életkort
- A CHH diagnózis felállítása igen nehéz
- Sokszor csak késői tinédzser vagy felnőtt korban születik diagnózis
- Szemben más ritka betegségekkel, a CHH kezelhető
- A kezelés élethosszig tart és rendszeres orvosi ellenőrzést igényel
- Konzultáljon kezelőorvosával a legmegfelelőbb terápia kiválasztásához
- A kezelés megszakítása az egészséget veszélyezteti
- Termékenység a legtöbb esetben megfelelő hormon kezeléssel elérhető
- A CHH öröklődhet, így a genetikai tanácsadás ajánlott
- A CHH súlyos pszichés zavarokat okoz egyes betegekben
- A kezelőorvos és a CHH beteg csoportok értékes támogatást képesek nyújtani

Hasznos szakirodalom
http://www.nature.com/nrendo/journal/v11/n9/full/nrendo.2015.112.html

Hasznos weboldalak
http://www.gnrhnetwork.eu/hhn_home/hhn-patients_familles_gnrh_deficiency_kallmann_syndrome/hhn-onlinesupportgroups.htm

https://www.rareconnect.org/en/community/kallmann-syndrome

https://www.facebook.com/KallmannSyndrome/

Italiano

Cosa dovresti sapere sull'Ipogonadismo Ipogonadotropo Congenito (CHH) e la sindrome di Kallmann (KS)

Cos'è il CHH?

CHH è causato dal deficit di **GnRH (ormone di rilascio delle gonadotropine)**. Quando si associa alla mancanza di olfatto (anosmia) è noto come sindrome olfatto-genitale o sindrome di Kallmann (KS). Tutte queste forme vengono diagnosticate e trattate nello stesso modo.

Cosa fa il GnRH?

Il GnRH è un ormone essenziale per lo sviluppo sessuale, la pubertà e la fertilità.

1. Il GnRH è rilasciato dall'ipotalamo, un'area del cervello che controlla varie funzioni del corpo.
2. Il GnRH agisce direttamente sulla ghiandola ipofisaria (una ghiandola delle dimensioni di un pisello sita nel cervello nelle vicinanze dell'ipotalamo) inducendo il rilascio di due ormoni: l'ormone luteinizzante (LH) e l'ormone follicolo stimolante (FSH).
3. LH e FSH sono importanti per la pubertà e la fertilità
   - nel maschio: LH e FSH stimolano i testicoli a produrre testosterone e spermatozoi
   - nella femmina: LH e FSH stimolano le ovaie a produrre estrogeni e progesterone che sono importanti per la fertilità.

Cosa succede in assenza di GnRH?

L'assenza parziale o completa di GnRH comporta una secrezione ridotta o assente di LH e FSH. Ne consegue un mancato sviluppo puberale e infertilità (vedi la figura sottostante). Questo significa che:

- nel maschio: 1) i testicoli non si sviluppano, 2) il testosterone non viene prodotto in quantità normali, e 3) gli spermatozoi non si sviluppano.
- nella femmina: 1) sebbene presenti, gli ovociti non si sviluppano o non crescono e non vengono rilasciati (ovulazione), 2) estrogeni e progesterone non vengono prodotti, e 3) non sono presenti le mestruazioni.

Segni di mancata pubertà negli adolescenti e nei giovani adulti

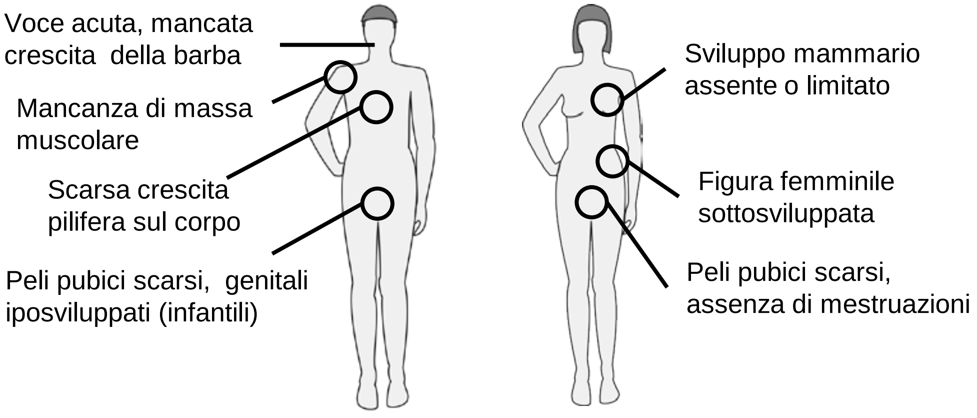

Questi difetti sono quasi sempre presenti alla nascita (detti congeniti). Tuttavia, la diagnosi viene fatta spesso in età adolescenziale o all'inizio dell'età adulta quando la pubertà non inizia. **È importante sapere che questa condizione è curabile. Nella maggior parte dei casi richiede una terapia ormonale sostitutiva e un'assistenza continua per tutta la vita.**

2017: www.gnrhnetwork.eu / marco.bonomi@unimi.it; luca.persani@unimi.it

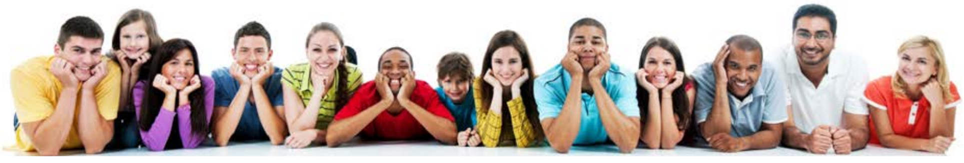

Chi è affetto da CHH?
Sono affetti sia maschi che femmine. Il CHH è diagnosticato più frequentemente nei maschi. La ragione di ciò non è chiara. Potrebbe essere che molte donne affette vengano viste dal ginecologo e venga loro prescritta una pillola contraccettiva per conseguire cicli regolari, senza che venga fatta una precisa diagnosi. Alcuni pazienti non arrivano mai ad una diagnosi corretta e possono non essere mai visti da uno specialista per il deficit di GnRH.

Quante persone sono affette?
CHH è una malattia rara quindi è difficile fare una stima precisa. Riteniamo che CHH colpisca all'incirca da 1 su 4.000 a 1 su 10.000 persone. Ciò significa che il numero intero di persone con CHH in Europa (circa 74.000) potrebbe riempire lo stadio Olimpico di Berlino in Germania.

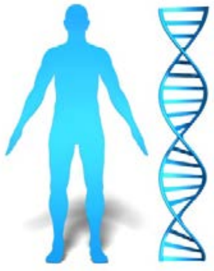

CHH viene ereditato? CHH può essere ereditario. Questo significa che **può essere trasmesso** attraverso le generazioni e all'interno delle famiglie. In molti casi non vi è alcuna storia familiare apparente. Una causa genetica viene identificata circa metà (50%) delle volte. Vi sono studi in corso e rimane ancora molto da scoprire riguardo alle cause genetiche di CHH. Nella maggior parte dei casi, è difficile per gli operatori sanitari prevedere in modo affidabile e preciso se un paziente passerà CHH ai propri figli.

Metà dei pazienti ha una causa genetica nota, metà non ha una causa genetica nota

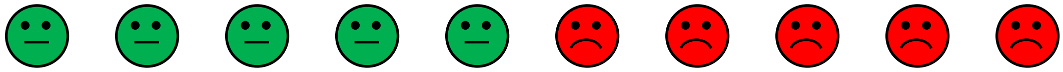

CHH è complicato perché in alcuni casi CHH può essere causato dalla combinazione di due o più varianti (mutazioni) in diversi geni. Questo rende **difficile predire se il CHH verrà trasmesso ai figli**. Per un gene, ANOS1 (KAL1), è facile da predire. Sono necessari ulteriori studi per comprendere meglio questo aspetto e migliorare la diagnosi.

Come viene diagnosticato il CHH?
CHH è difficile da diagnosticare. C'è un'ampia variabilità nel normale momento di inizio della pubertà. Ciò rende difficile stabilire se la pubertà sia ritardata (ma normale), o se invece si tratti di CHH e siano quindi necessarie terapie. Sono necessari dei controlli ambulatoriali regolari per valutare la progressione della pubertà. Ai pazienti andrebbe sempre chiesto riguardo al difetto di olfatto. Segni indicativi di CHH sono:

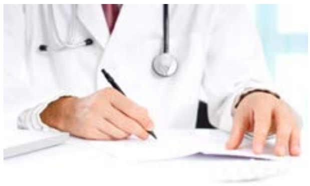

- **maschi-** assenza di segni di pubertà a 16 anni in presenza di bassi livelli di testosterone, LH e FSH.
- **femmine –** assente sviluppo mammario a 14-15 anni e mancanza di mestruazioni a 16-17 anni con livelli di estrogeni, LH e FSH.
- Ci sono **altri accertamenti che devono essere fatti** per escludere che non vi siano altri problemi a causare i bassi livelli ormonali. Tali accertamenti includono esami ematici e studi di diagnostica per immagine (radiografie, ecografie e RMN)

Ci sono segni precoci da cercare?
Per molte persone non ci sono segni di CHH prima del fallimento della pubertà.

Alcuni neonati maschi posso presentare segni suggestivi per CHH come il **micropene o la mancata discesa testicolare** (criptorchidismo).

Il micropene può essere trattato in età infantile con trattamenti ormonali. I testicoli ritenuti andrebbero corretti chirurgicamente già nel primo anno di vita per favorire la futura fertilità.

Ci sono altri segni aggiuntivi, non dovuti a CHH, che possono essere presenti in alcuni ma non in tutti i pazienti.

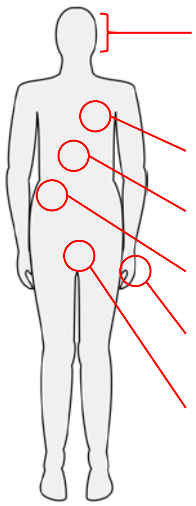

Possibili altri segni

In che modo l'assenza di pubertà può influire sull'individuo?
L'assenza di sviluppo puberale in contemporanea con i coetanei **può essere un evento stressante e talora persino traumatico.** Questo può essere un problema importante per alcuni pazienti con CHH. L'impatto, in alcuni casi, può essere grave sebbene possa variare da persona a persona. L'adolescenza può essere un momento critico anche per gli adolescenti che iniziano la pubertà regolarmente, ma la mancanza di sviluppo puberale e l'isolamento da parte dal gruppo dei coetanei può potenzialmente portare a:

- bassa autostima, scarsa fiducia in se stessi
- timidezza, difficoltà di interazione con i coetanei
- ansia, depressione
- scarsa immagine di sè

Il senso dell'olfatto anormale può incidere sul senso del gusto e sul piacere del cibo. Le persone potrebbero non essere capaci di rilevare il gas (o quando i cibi sono deteriorati). Alcuni possono essere preoccupati per l'odore del corpo.

Cosa puoi fare?
Nel complesso questi problemi possono essere superati. L'aiuto psicologico e le terapia possono essere utili.

1. **Parla con i tuoi medici** - potrebbero non rendersi conto che stai passando un periodo difficile. I medici possono aiutarti a trovare consulenza e sostegno.
2. **Entra in contatto con altri pazienti -** l'uso di gruppi in rete (Facebook, RareConnect.org) e gruppi di supporto faccia-a-faccia possono essere molto utili. Si tratta di spazi dove i pazienti possono discutere di questioni che sono importanti per loro. Gli altri pazienti sanno cosa significa vivere giorno per giorno con CHH e possono fornire consigli pratici e supporto.

Cosa dovresti fare per essere in buona salute?
Avere CHH non dovrebbe accorciare la tua vita. Ci sono cose che puoi fare per rimanere in buona salute

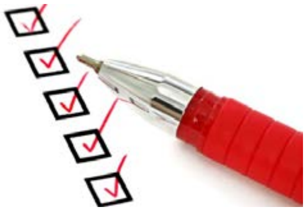

- mantenere l'assistenza sanitaria e le visite
- assumere i tuoi trattamenti come prescritto
- mantenere una dieta salutare
- svolgere una regolare attività fisica
- non fumare tabacco

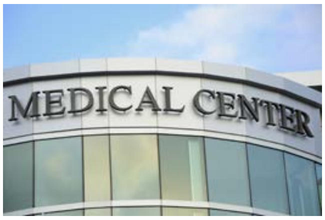

CHH è curabile?

Attualmente **non esiste una cura per CHH**. E’ molto difficile curare una malattia congenita (genetica). Sono in corso ricerche per vedere se siamo in grado di ripristinare la produzione di GnRH dall'ipotalamo. Questi studi sono ancora in una fase molto iniziale, ma si spera che questo sia possibile in futuro.

Ci sono terapie disponibili per CHH?

Sono disponibili terapie per lo sviluppo dei segni esteriori della pubertà - come la crescita della barba nei maschi e lo sviluppo mammario nelle femmine. Sono inoltre disponibili trattamenti ormonali specifici per aiutare a indurre la fertilità nella maggior parte casi.

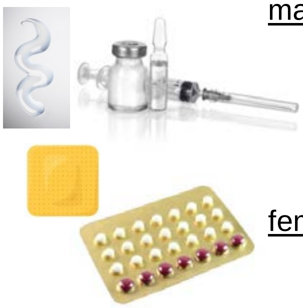

maschi: il **testosterone** (iniezioni o gel applicato sulla cute) è il trattamento più comune per favorire la comparsa di segni puberali. Il trattamento induce la crescita staturale, l’abbassamento del tono della voce, la crescita della barba, la crescita del pene, il desiderio sessuale (libido) e la funzione sessuale - ma non la fertilità.

femmine: l’estrogeno a basse dosi (compresse o cerotti) induce la crescita staturale e favorisce lo sviluppo mammario, il desiderio sessuale (libido) e la tipica conformazione femminile; quando viene combinato con il progesterone causa il regolare ciclo mestruale – ma non la fertilità

Quando si inizia il trattamento **la dose va incrementata gradualmente**. Questo può essere frustrante per alcuni pazienti che potrebbero aspettarsi risultati rapidi. Tuttavia, ciò è importante per massimizzare la crescita staturale (e lo sviluppo mammario nella femmina).

Alcuni pazienti con CHH recuperano dopo il trattamento e possono produrre normali livelli ormonali. Si parla di forme «*reversal*». Le ragioni di questo recupero non sono note e non siamo al momento in grado di predire chi lo avrà. **Questo recupero non è sempre duraturo. Pertanto è importante continuare ad essere seguiti da uno specialista.**

Circa 1 paziente su 10 (10%) con CHH ha un recupero

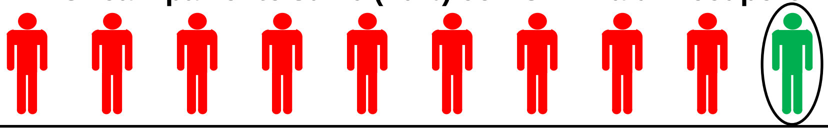

Una persona con CHH può diventare fertile?

Si, il **CHH è una forma trattabile d’infertilità.** La maggioranza - ma non tutte- le persone con CHH possono diventare fertili con **specifici trattamenti ormonali**.

- Le terapie specifiche possono essere sia iniezioni ormonali diverse volte alla settimana, sia una pompa portatile per GnRH (simile alla pompa usata dai diabetici).
- La terapia per la fertilità deve essere fatta da specialisti esperti (**endocrinologi della reproduzione**).
- a volte (ma non sempre) è necessaria una fertilità assistita, come la fertilizzazione *in vitro* (IVF).

Fertilità: nel complesso, 3 su 4 pazienti (75%) hanno successo

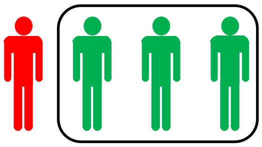

maschi: può richiedere fino a 2 anni per produrre spermatozoi

femmine: in alcuni casi la fertilità viene conseguita in pochi mesi

2017: www.gnrhnetwork.eu / marco.bonomi@unimi.it; luca.persani@unimi.it

Ci sono dei rischi se CHH non è trattato?

Sebbene il testosterone e l'estrogeno non siano ormoni vitali, la loro assenza (o carenza) può avere serie conseguenze **sulla salute, sulla funzione sessuale e sulla qualità della vita**.

<u>Nei maschi e nelle femmine:</u>

- aumento del rischio di **bassa densità ossea (osteoporosi)** – questo significa che le ossa sono fragili e c'è un rischio aumentato di fratture. Se CHH non è trattato, l'osteopeorosi può colpire la persona a qualsiasi età. Alcune persone con CHH necessitano di trattamenti aggiuntivi per l'osteoporosi.
- **ridotta funzione sessuale e desiderio** sono il risultato di bassi valori ormonali
- **stanchezza, debolezza e depressione** sono sintomi comuni nei pazienti senza trattamento

I maschi senza trattamento hanno anche un rischio più alto rischio di avere problemi metabolici come il pre-diabete o il diabete. **Questi rischi possono essere diminuiti stando in terapia.**

Punti chiave:

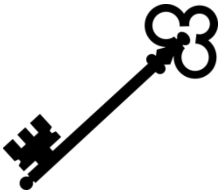

- CHH è una condizione rara causata dalla carenza di GnRH
- CHH causa un'assente pubertà e infertilità
- CHH <u>non dovrebbe</u> ridurre l'aspettativa di vita
- CHH è difficile da diagnosticare
- molte persone non vengono diagnosticate fino alla tarda adolescenza o l'inizio dell'età adulta
- diversamente da molte malattie rare, ci sono trattamenti disponibili
- necessiti di un trattamento per tutta la vita e di regolari controlli medici
- parla con il tuo medico circa le opzioni terapeutiche per stabilire la migliore per te
- interrompere il trattamento ha conseguenze negative per la tua salute e il tuo benessere
- la fertilità è possibile nella maggior parte dei casi con specifici trattamenti ormonali
- poi trasmettere CHH ai tuoi figli, quindi è raccomandata una consulenza genetica
- CHH è psicologicamente difficile per alcune persone
- puoi trovare supporto dai tuoi medici e dai gruppi di pazienti

Referenze utili

http://www.nature.com/nrendo/journal/v11/n9/full/nrendo.2015.112.html

Siti Web utili

http://www.gnrhnetwork.eu/hhn_home/hhn-patients_familles_gnrh_deficiency_kallmann_syndrome/hhn-onlinesupportgroups.htm

https://www.rareconnect.org/en/community/kallmann-syndrome

https://www.facebook.com/KallmannSyndrome/

선천성 성선자극호르몬결핍성 성선기능저하증 (CHH) 및 칼만 증후군(KS)에 대하여 알아야 할 점은 무엇인가?

선천성 성선자극호르몬결핍성 성선기능저하증 (CHH)이란 무엇인가?
성선자극 호르몬 결핍으로 발생하는 유전질환으로, 사춘기의 지연이나 결여를 특징으로 한다. 뇌의 후각신경조직 생성결핍으로 인한 후각상실증이 동반되는 경우에는 후각-성선기능 저하 증후군 또는 칼만증후군 (KS)이라고 불리며, 모두 같은 치료법이 적용된다.

성선자극호르몬이 하는 역할은?
성선자극호르몬은 사춘기에 일어나는 이차성징의 발달과 생식기능에 반드시 필요하다.

1. 성선자극호르몬은 신체의 여러가지 기능을 조절하는 시상하부라고하는 뇌의 부분에서 분비된다.
2. 분비된 성선자극호르몬은 뇌하수체 (시상하부 근처에 있는 완두콩만한 뇌의 구조)에 직접 작용하여 황체형성 호르몬과 난포자극호르몬을 생성 분비하게 한다.
3. 황체형성 호르몬과 난포자극호르몬은 사춘기와 생식기능에 필수적인 역할을 한다.
   - <u>남성의 경우</u>: 고환에서 테스토스테론과 정자 생성 촉진
   - <u>여성의 경우</u>: 난소에서 에스토로겐과 프로게스테론의 생성 촉진

성선자극호르몬이 결핍되면 어떤 문제가 발생하나?
성선자극호르몬이 부족하거나 완전히 결핍되면, 그로인한 황체형성 호르몬과 난포자극호르몬 결핍으로 이어져, 사춘기가 진행되지 못하며 불임이 된다 (아래 도표 참고)

- <u>남성의 경우:</u> 1) 고환이 성장하지 않음. 2) 테스토스테론이 정상 양으로 분비되지 않음. 3) 정자가 정상적으로 생성되지 않음.
- <u>여성의 경우</u>: 1) 난자가 존재하지만, 정상적으로 성장 배란되지 않음. 2) 에스트로겐과 프로게스테론이 분비되지 않음. 3) 생리 월경이 없음.

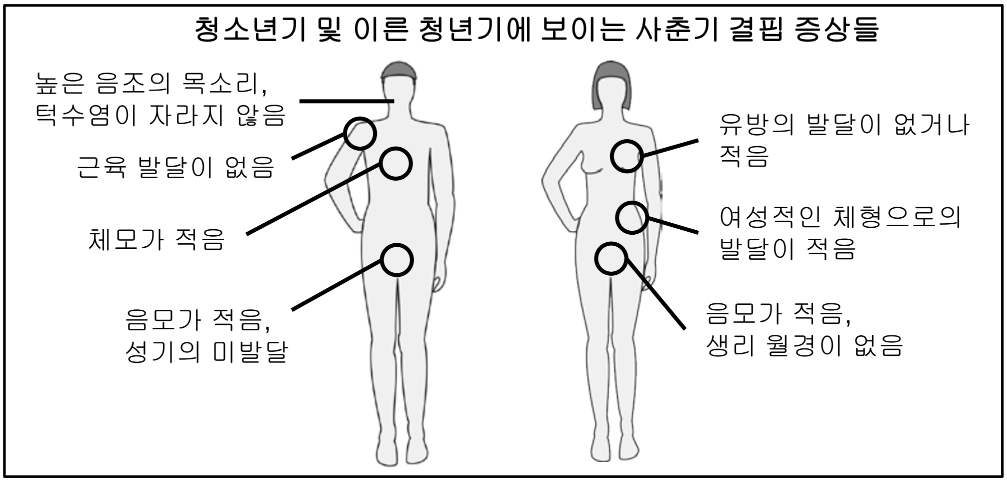

위의 증상들은 태어날때부터 발견되므로 선청성으로 간주됨. 그러나, 실제적 진단은 청소년기나 이른 청년기에 사춘기가 진행되지 않으므로 인해 진단받게됨. **중요한점은, 이러한 증상들은 치료가 가능하며, 다만, 대부분의 경우에 일생동안 호르몬 치료를 받고 지속적인 관리를 해야한다는 것이다.**

2017: www.gnrhnetwork.eu / andrew.dwyer@chuv.ch

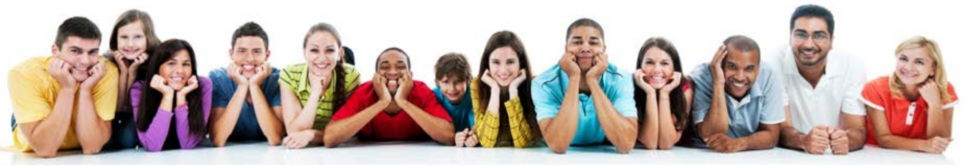

선천성 성선자극호르몬결핍성 성선기능저하증(CHH)은 누구에게 일어나나?

남녀 모두에서 발생하지만, 남자에게 좀 더 자주 나타난다. 그 이유는 아직 불분명하지만, 아마도, 불규칙한 월경주기를 보이는 여성들이 정확한 진단을 받지 못한 상황에서 산부인과 진찰을 통해 피임제등을 처방받아 증상치료를 했을 가능성이 높다. 즉, 환자들이 정확한 진단을 제대로 받지 못했거나 성호르몬 결핍증 관련 전문의를 찾지 않았을 경우가 많다.

얼마나 많은 사람들에게 발병하나?

이 질환은 희귀병으로서 정확하게 발병률을 알기 어렵다. 대략 4천에서 만명당 한명꼴로 나타난다고 보는데, 따라서 유럽 전체에서 약 7만4천명의 환자가 있을것으로 추측되며 이는 독일 베를린의 올림픽 주경기장을 채우는 숫자이다.

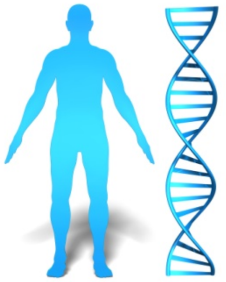

유전될 가능성은? 이 질환은 유전병으로서 가족내에서 **후손에게 전달 될수 있다.** 하지만, 많은 경우에 특별한 가족력이 없을수도 있다. 현재까지 약 50%의 경우에만 유전자 돌연변이가 확인되었다. 따라서 이 질병의 유전기작에 대한 연구가 계속 진행중이며, 아직도 발견해야 할 것들이 많다. 대부분의 경우, 이 질환이 자녀에게 유전될지에 대해 의료진이 정확하고 신뢰할수 있는 진단을 하기가 아직은 어렵다.

환자의 절반정도는 유전적 원인이 알려져 있으나, 나머지 절반은 알려진 정보가 없다.

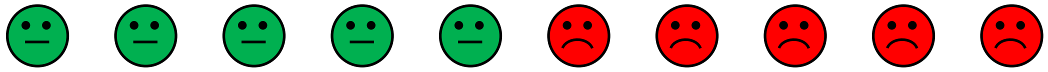

경우에 따라서는 2개나 그 이상의 다른 유전인자들에서 돌연변이가 발견되기도 하므로, 이 질환의 원인을 규명하는 것이 더욱 복잡해 질수 있고, **자녀에게 유전될 가능성을 정확히 예측하기 힘들다.** 비록 *ANOS1 (KAL1)* 이라는 유전자에 관해서는 비교적 정확한 예측이 가능하지만, 이 질환에 대한 더 나은 이해와 향상된 진단법을 찾기 위한 연구가 계속 필요하다.

CHH 진단은 어떻게 이루어지나?

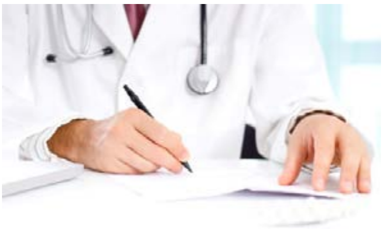

이 질환은 진단하기가 어렵다. 정상인에서도 사춘기의 시기는 개인별로 편차가 크기 때문에, 사춘기 발달이 좀 지체되었지만 정상인지, 아니면 치료가 필요한 질환 인지를 결정하는 것이 쉽지 않을수있다. 사춘기 발달 장애가 의심될 경우 규칙적인 내진을 통해 그 진행을 관찰해야 한다. 환자에게 후각상실이 있는지를 항상 확인해야 한다. 성선기능저하의 증상은:

- **남아의 경우** - 16세가 될때까지 사춘기 발달이 없고, 테스토스테론, 황체형성 호르몬, 난포자극호르몬의 혈중 농도가 낮다.
- **여아의 경우** - 14~15세가 될때까지 유방의 발달이 없고, 16~17세가 될때까지 생리 월경이 없다. 에스트로겐, 황체형성 호르몬, 난포자극호르몬의 혈중 농도가 낮다.
- 여러가지 검사를 통하여 이러한 호르몬 분비 저하가 다른 원인때문이 아닌지를 확인해야 한다. 예를 들어, 피검사나 영상진단 (X-ray, 초음파, MRI)등을 해야할수도 있다.

주의깊게 봐야할 조기 증상이 있나?

대부분의 경우 사춘기전에는 특별한 증상이 없다.

남아의 경우, 비정상 성기 (즉, **음경이 아주 작거나 고환이 복강에서 내려오지 않은 경우**)가 있을수 있다.

음경은 유아나 어린이시기에 호르몬 치료로 성장을 유도할수 있고, 복강내 고환은 1살 미만 유아의 경우 수술을 하면 정상으로 회복될수 있다.

이외에도 성선기능저하와는 무관한 몇가지 다른 증상들이 일부 환자에게서는 추가적으로 발견될수 있다.

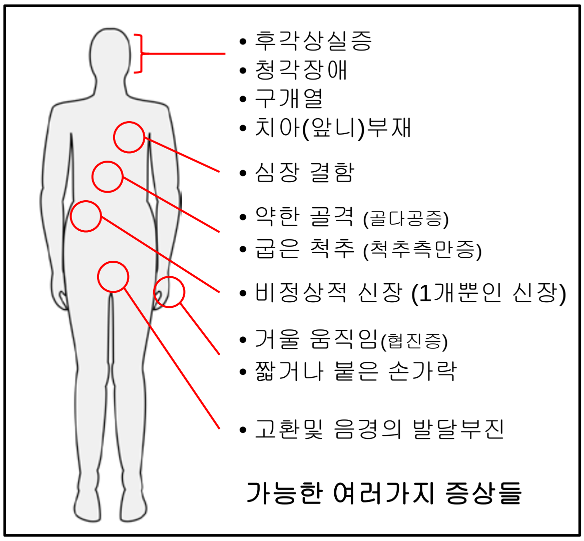

가능한 여러가지 증상들

사춘기가 없으면 개인적으로 어떤 영향을 받나?

남들과 비슷한 시기에 정상적인 사춘기를 겪지 않는것은 상당히 큰 스트레스가 될수 있으며 일부 환자들에게는 심각한 정신적 상처가 될수 있다. 개인별로 정도의 차이가 있을수는 있으나, 대부분의 청소년들은 사춘기 시절에 이미 여러가지 정신적 스트레스를 겪는데, 이시기에 친구들에 비하여 정상적인 성발달에서 뒤쳐지는 것은 청소년들에게 상당히 큰 문제를 야기할수 있다. 예를 들어:

- 자아 존중감 저하, 자신감 상실
- 수줍음, 친구들과의 교제가 어려움
- 불안증, 우울증
- 부정적 신체이미지

비정상적인 후각은 미각에도 영향을 미치기 때문에 음식 맛을 즐기지 못할수 있다. 가스냄새 (또는 상한 음식)를 감지하지 못하기도 한다. 일부의 경우 자신의 체취에 대해 걱정을 하기도 한다.

어떤 해결책이 있나?

정신과 상담과 심리치료를 통하여 이러한 문제들은 대부분 극복될수 있습니다.

1. **의료진과 상의를 하십시요** – 다른 사람들은 여러분이 얼마나 힘든 시간을 겪는다는것을 잘 모를수도 있읍니다. 전문적인 상담과 도움을 받으십시요.
2. **다른 환자들과 소통하십시요** - 인너텟상에서 온라인 그룹 (Facebook, RareConnect.org)에 참여를 하거나 직접 대면하는 환자모임등이 도움이 될수 있읍니다. 이러한 기회를 통하여 환자들이 서로의 고통을 호소하고 이 질병을 가지고 살아가는 일상에서의 어려움을 극복할수 있는 조언과 정보들을 교환할수 있읍니다.

나의 건강을 지키기 위하여 어떻게 해야하나?

이 질환은 수명에는 영향을 미치지 않습니다. 자신의 건강을 유지하기 위하여 아래와 같은 일들을 할수 있읍니다.

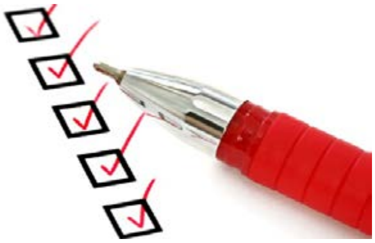

- 의료진과의 상담과 진찰을 꾸준히 받는다.
- 처방받은 치료를 반드시 실천한다.
- 건강한 식단
- 규칙적인 운동
- 금연

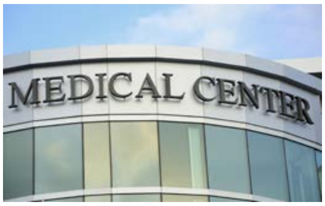

CHH는 치료 가능한가?

선천성 유전병은 고치기가 힘들기 때문에 현재상태로는 **치료법이 없다**. 하지만, 뇌하수체에서 성선자극호르몬의 생성기능을 회복시키기 위한 여러가지 연구가 진행중이다. 이러한 연구는 아직 초기 단계이지만 미래에 실현 가능성이 있다.

현재 가능한 치료방법은 무엇인가?

사춘기 이차성징 발달을 유도하기 위한 치료가 가능하다 – 예를 들어 남성의 수염을 자라게 하거나 여성의 유방을 발달시킬수 있다. 특별한 호르몬 치료를 통하여 많은 경우 불임을 치료할수도 있다.

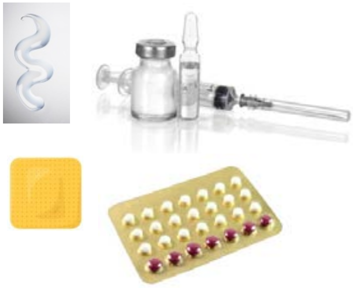

<u>남성</u>: 테스토스테론 (피부에 젤을 도포하거나 주사) 이 사춘기를 유도하는 가장 일반적인 치료제이다. 이를 통하여 사춘기의 신체적 성장, 변성, 수염, 성기 발달, 성욕, 성기능 발달등을 유도할수 있다 (임신은 제외).

<u>여성</u>: 적은양의 **에스트로겐** (피부에 붙이거나 알약복용) 투여를 통하여 사춘기의 신체적 성장, 유방 발달, 성욕, 여성다운 체형을 유도할수 있으며, 프로게스테론과 병합사용시 규칙적인 월경 생리주기를 이룰수 있다 (임신은 제외).

처음 치료를 시작할때의 **최소 호르몬 복용량을 서서히 조절하며 증가해야한다.** 따라서 빠른 효과를 기대했던 환자라면 실망을 느낄 수도 있다. 그러나, 최대한의 성기능 성장효과 (여성의 경우 유방 발달 포함)를 얻는 것이 가장 중요하므로, 이러한 절차를 따라야 한다.

일부 환자의 경우에는 치료후 완치가 되는 수도 있으며 정상인 수준의 호르몬을 생성할 수 있게 된다. 이러한 경우를 반전이라고 부르는데, 그 정확한 이유는 아직 밝혀지지 않았으며, 어떤 환자가 반전을 하게 되는지를 예측할 수가 없다. 또한, 반전이 항상 지속되는 것이 아니기에, 전문의료진이 지속적으로 관찰하는 것이 중요하다.

질환을 가진 열명중 한명 (10%)의 환자가 반전이 될수 있다.

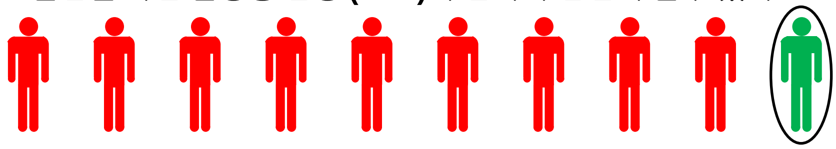

성선자극호르몬결핍성 성선기능저하증을 가진 환자가 임신을 할수 있나?

CHH는 **치료가 가능한 불임질환**중의 하나이다. 모두는 아니지만 대부분의 경우, 이 질환을 가진 환자도 특별한 호르몬 치료를 받으면 임신할수있다.

- 특별한 호르몬 주사를 일주일에 몇 번 맞거나, 성선자극호르몬 펌프 (당뇨환자에게 사용되는 인슐린 펌프와 비슷한 휴대용 펌프)를 착용하는 방법이 있다.
- 불임치료는 경험이 있는 전문의료진 (생식내분비 전문의) 의 진단을 요한다.
- 늘 그렇지는 않지만 흔히 인공수정등과 같은 임신보조 치료가 필요하다.

임신: 대략 4명중 3명의 환자 (75%)가 임신에 성공한다.

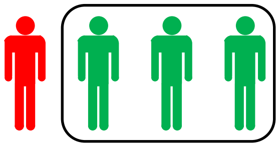

<u>남성</u>: 정자를 생산하는데 최대 2년까지 걸릴 수 있다.
<u>여성</u>: 경우에 따라서는 수개월내에 임신이 될 수도 있다.

2017: www.gnrhnetwork.eu / andrew.dwyer@chuv.ch

CHH에 필요한 치료를 받지 않을시 발생할 위험은?
테스토스테론과 에스트로겐의 결핍이나 부족이 생명에 위협을 주지는 않으나 방치했을시에는 심각한 건강문제와 성기능 및 삶의 질에 영향을 줄수 있다.

<u>남성과 여성 모두에서</u>

- 골빈도가 낮아질 위험이 커짐 **(골다공증)** – 뼈가 약해지고 골절상을 입을 위험이 높아진다. CHH 때문에 생긴 골다공증은 연령에 상관없이 올수 있으며, CHH 환자는 특히 추가적인 치료를 필요로 한다.
- 성 호르몬 결핍으로 인한 **성기능 장애및 성욕저하**
- 치료를 중단한 환자의 경우에 **피로감, 우울증, 의욕저하**등이 흔히 나타난다.

남성 환자가 치료를 받지 않았을 경우 당뇨병 전 증후군 또는 당뇨병등의 대사기능 장애가 올 확률이 높아진다. **이러한 위험은 치료를 지속해야만 낮아질수 있다.**

중요 개요:

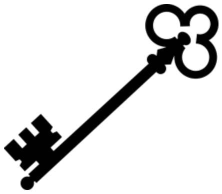

- 선천성 성선자극호르몬결핍성 성선기능저하증은 성호르몬 부족 때문에 생긴 휘귀병이다.
- 이 질환은 사춘기 이차성징 발달부재와 불임을 야기한다.
- 이 질환은 수명에는 지장이 없다.
- 이 질환은 정확한 진단이 어렵다.
- 많은 환자의 경우 십대 청소년기나 어린 청년기에 진단을 받게된다.
- 휘귀병임에도 불구하고 치료가 가능하다.
- 의료진과의 정기적인 상담하에 일생동안 치료를 받아야 한다.
- 주치의와 의논을 해서 본인에게 가장 적합한 치료방법을 찾아야 한다.
- 치료를 중단했을 경우 건강과 삶의 질에 나쁜 영향을 미치게 된다.
- 특별한 호르몬 치료를 통하여 임신이 가능한 경우가 많다.
- 이 질환은 유전병이므로 자녀에게 대물림될 가능성이 있다. 따라서 가족계획시 전문가의 상담을 받을것을 권유한다.
- 이 질환은 심리적 정신적 고통을 유발할수 있다.
- 전문 의료진과 환자 모임등을 통하여 도움을 받을수 있다.

유용한 참고문서
http://www.nature.com/nrendo/journal/v11/n9/full/nrendo.2015.112.html

유용한 웹사이트
http://www.gnrhnetwork.eu/hhn_home/hhn-patients_familles_gnrh_deficiency_kallmann_syndrome/hhn-onlinesupportgroups.htm

https://www.rareconnect.org/en/community/kallmann-syndrome

https://www.facebook.com/KallmannSyndrome/

Co należy wiedzieć o wrodzonym hipogonadyźmie hipogonadotropowym (congenital hypogonadotropic hypogonadism; CHH) i zespole Kallmanna (Kallmann syndrome; KS)

Co to jest CHH?

CHH jest spowodowany **niedoborem GnRH (hormon uwalniający gonadotropiny)**. Niedobór GnRH połączony z brakiem węchu (anosmią) określany jest zespołem węchowo-płciowym lub zespołem Kallmanna (KS). Powyższe zaburzenia są diagnozowane i leczone w ten sam sposób.

Jak działa GnRH?

GnRH warunkuje rozwoju płci, dojrzewanie oraz płodność:

1. GnRH jest uwalniany z podwzgórza, obszaru mózgu, który kontroluje szereg funkcji ciała.
2. GnRH oddziałuje bezpośrednio na przysadkę (gruczoł wielkości ziarna grochu znajdujący się w mózgu) uwalniając dwa hormony: hormon luteinizujący (LH) i hormon folikulotropowy (FSH).
3. LH i FSH warunkuje dojrzewanie i płodność
   • u mężczyzn: LH i FSH stymulują jądra do produkcji testosteronu i nasienia
   • u kobiet: LH i FSH stymulują jajniki do produkcji estrogenu i progesteronu, które warunkują płodność

Co się stanie w przypadku braku GnRH?

Całkowity lub częściowy niedobór GnRH prowadzi do braku lub bardzo niskiego stężenia LH i FSH. Wynikiem tego jest **brak dojrzewania oraz niepłodność** (patrz rycina poniżej). To oznacza, że:

- u mężczyzn: 1) jądra nie powiększają się, 2) testosteron nie jest produkowany w prawidłowych ilościach i 3) nasienie nie jest wytwarzane

- u kobiet: 1) pomimo obecności komórek jajowych, ich rozwój i wzrost jest zahamowany, co uniemożliwia ich uwalnianie (owulację), 2) estrogen i progesteron nie są produkowane i 3) miesiączki (krwawienia) nie występują.

Objawy braku dojrzewania u młodzieży i młodych dorosłych

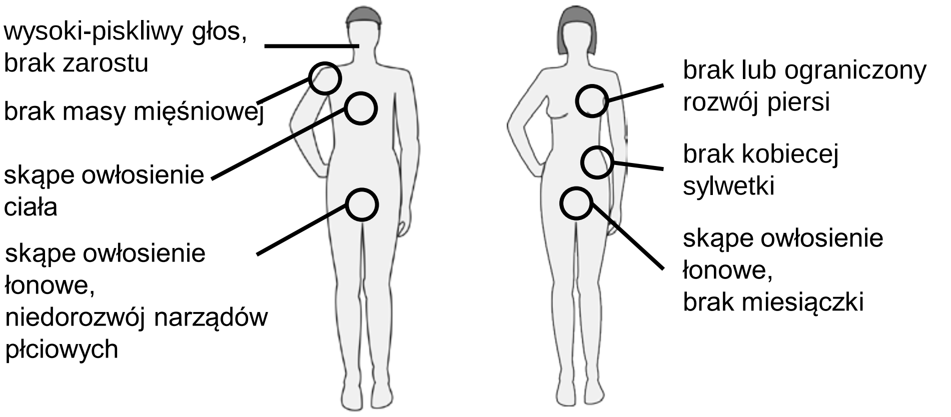

Problemy te są prawie zawsze obecne od urodzenia (określane wrodzonymi), ale często dopiero brak dojrzewania w okresie młodzieńczym pozwala postawić diagnozę. **Najważniejsze, że leczenie jest możliwe. W większości przypadków terapia hormonalna/zastępcza jest prowadzona przez całe życie a pacjenci wymagają stałej opieki.**

2017: www.gnrhnetwork.eu / andrew.dwyer@chuv.ch / marek.niedziela@ump.edu.pl

Kto choruje na CHH?

Na CHH chorują zarówno mężczyźni jak i kobiety. CHH jest częściej rozpoznawany u mężczyzn. Powód tego nie jest jasny. Możliwe jest, że wiele kobiet dotkniętych CHH jest konsultowanych przez ginekologa i otrzymują receptę na „pigułkę” antykoncepcyjną aby mieć regularne miesiączki i dlatego nie można ustalić jednoznacznego rozpoznania. Niektórzy pacjenci nigdy nie otrzymają właściwego rozpoznania i może nigdy nie spotkają się ze specjalistą od niedoboru GnRH.

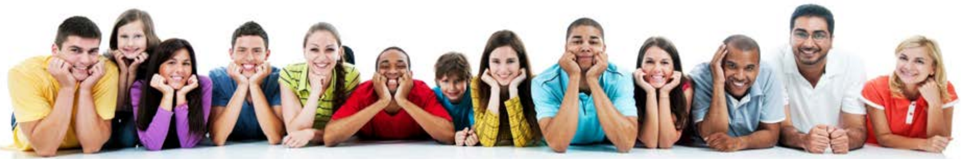

Ile osób jest dotkniętych tą chorobą?

CHH występuje rzadko i dlatego dokładne oszacowanie częstości występowania jest trudne. Wydaje się, że CHH występuje u około 1 na 4'000 do 10'000 osób. Oznacza to, że całkowita liczba pacjentów z CHH w Europie (czyli około 74'000) mogłaby wypełnić stadion Olimpijski w Berlinie (Niemcy).

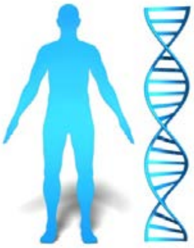

Czy CHH jest dziedziczny?

CHH może być dziedziczny. Oznacza to, że **może być przekazywany z pokolenia na pokolenie** i w obrębie rodzin. W wielu przypadkach wywiad rodzinny nie jest obciążony. W około połowie przypadków (50%) możemy już zidentyfikować przyczynę genetyczną. Prowadzone są dalsze badania aby odkryć kolejne przyczyny genetyczne CHH. W większości przypadków lekarze nie potrafią przewidzieć z jakim prawdopodobieństwem pacjent przekaże CHH swoim dzieciom.

U połowy pacjentów znana jest przyczyna genetyczna, a u połowy przyczyna ta nie jest znana

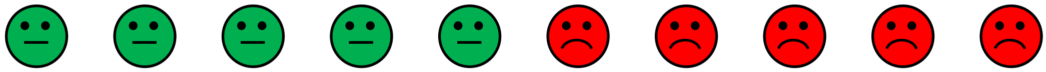

CHH może być jeszcze bardziej skomplikowanym schorzeniem, gdyż w niektórych przypadkach może być spowodowany obecnością dwóch lub więcej zmian (mutacji) w różnych genach. **Określenie ryzyka przekazania choroby potomstwu w takich przypadkach jest jeszcze trudniejsze.** Ryzyko to jest łatwo przewidzieć dla jednego genu - *ANOS1* (*KAL1*). Potrzebujemy więcej badań aby lepiej zrozumieć i rozpoznawać CHH.

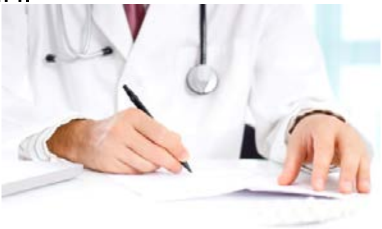

Jak rozpoznawany jest CHH?

Trudno jest postawić rozpoznanie CHH. Zakres normy czasowej dla okresu dojrzewania jest szeroki. To sprawia, że bardzo trudno jest stwierdzić czy dojrzewanie jest opóźnione (ale prawidłowe), lub czy jest to już CHH, który wymaga leczenia. Wówczas konieczne są regularne wizyty aby śledzić postęp dojrzewania. Pacjenci powinni być zawsze pytani o zaburzenia węchu. Objawy sugerujące CHH to:

- **u chłopców** – brak cech dojrzewania po 16 r. ż. wraz z towarzyszącym niskim stężeniem testosteronu, LH i FSH.

  u dziewcząt – brak rozwoju piersi po 14-15 r. ż. i brak miesiączki (krwawienia) po 16 r. ż. wraz z niskim stężeniem estrogenu, LH i FSH.
- **Należy wykonać dodatkowe badania** aby udowodnić, że nie ma innych przyczyn obniżonego stężenia hormonów. Badania obejmują testy krwi i badania obrazowe (RTG, USG i MR).

2017: www.gnrhnetwork.eu / andrew.dwyer@chuv.ch / marek.niedziela@ump.edu.pl

Czy są jakieś wczesne objawy, których należy poszukiwać?

U wielu osób nie występują żadne objawy CHH do czasu braku dojrzewania.

Niektóre niemowlęta płci męskiej mogą posiadać cechy sugerujące CHH, takie jak **małe prącie lub niezstąpione jądra** (wnętrostwo).

Małe prącie (mikropenis) można leczyć hormonalnie w okresie niemowlęcym / dziecięcym. Niezstąpione jądra powinny być skorygowane chirurgicznie wcześnie, w pierwszym roku życia w celu zachowania przyszłej płodności.
Są jeszcze inne objawy, nie związane z CHH, które występują u niektórych tylko pacjentów.

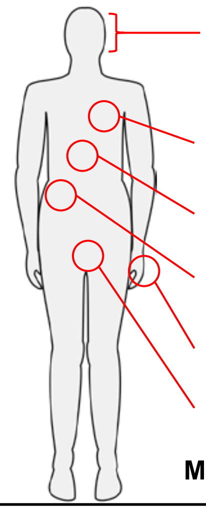

Możliwe inne objawy

Jaki wpływ może mieć brak dojrzewania?

Nie przechodzenie przez okres dojrzewania w tym samym czasie, co rówieśnicy **może być bardzo stresujące a nawet traumatyczne.** Dla wielu pacjentów z CHH może stanowić to istotny problem, który może mieć dotkliwe konsekwencje. Nastolatkowie, którzy rozpoczynają dojrzewanie o czasie, ale u których dojrzewanie prawidłowo nie postępuje, pozostają w tyle, w stosunku do rówieśników, co może powodować potencjalne problemy takie jak:

- niska samoocena, brak pewności siebie
- nieśmiałość, trudności w interakcji z rówieśnikami
- lęk, depresja
- poczucie „gorszego" wyglądu

Nieprawidłowy węch może mieć wpływ na zmysł smaku i przyjemność z jedzenia. Takie osoby mogą nie być w stanie wykryć gaz (lub zepsutą żywność). Niektórzy z nich mogą być zaniepokojeni o swój zapach ciała.

Co możesz zrobić?

Zwykle, wyżej wymienione dolegliwości mogą być przezwyciężane, ale porada psychologiczna i terapia mogą być przydatne:

1. **porozmawiaj ze swoimi lekarzami** - oni mogą nie zdawać sobie sprawy, że masz trudny okres. Pomogą Tobie znaleźć poradę i wsparcie.
2. **skontaktuj się z innymi pacjentami –** poprzez grupy internetowe (Facebook, RareConnect.org) czy grupy wsparcia (bezpośredni kontakt „twarzą w twarz"). Są to miejsca gdzie pacjenci mogą rozmawiać o sprawach, które są dla nich ważne. Inni pacjenci rozumieją co to znaczy żyć dzień za dniem z CHH i mogą dostarczyć praktycznych rad i wsparcia.

Co powinieneś zrobić, aby być zdrowym?

CHH nie powinien wpłynąć na skrócenie twojego życia. Są rzeczy, które możesz zrobić aby pozostać zdrowym:

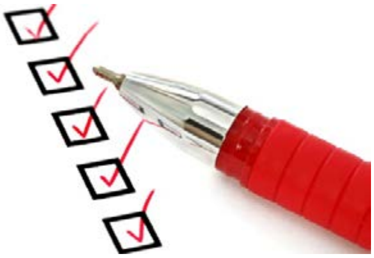

- być pod stałą opieką zdrowotną
- stosować przepisane leczenie
- zdrowo się odżywiać
- regularnie ćwiczyć
- nie palić tytoniu

2017: www.gnrhnetwork.eu / andrew.dwyer@chuv.ch / marek.niedziela@ump.edu.pl

Jakie sposoby leczenia CHH są dostępne?

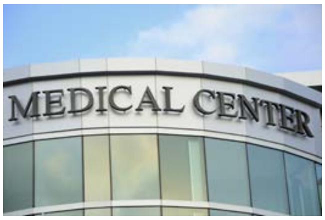

Czy CHH jest wyleczalny?

W chwili obecnej, **nie można wyleczyć CHH**. Bardzo trudno jest wyleczyć wrodzoną (genetyczną) chorobę. Prowadzone są wstępne badania nad możliwością przywrócenia produkcji GnRH w podwzgórzu. Mamy ogromną nadzieję, że dzięki nim będzie to możliwe w niedalekiej przyszłości.

Na czym polega leczenie CHH?

Dzięki dostępnemu leczeniu możliwy jest rozwój zewnętrznych objawów dojrzewania – takich jak zarost u mężczyzn i rozwój piersi u kobiet. dostępne są także Ponadto specjalistyczne leczenie hormonalne pozwala w większości przypadków wspomagać płodność:

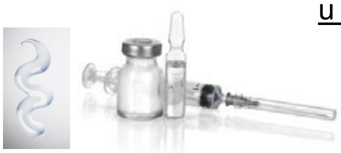

u mężczyzn: **testosteron** (w postaci żelu na skórę lub zastrzyków) jest najczęstszym sposobem leczenia rozwoju dojrzewania. Leczenie stymuluje wzrastanie, obniżenie barwy głosu, zarost, rozwój prącia, popęd płciowy (libido) i funkcję seksualną, ale nie płodność.

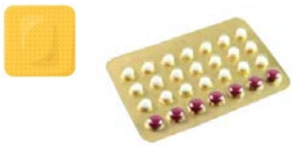

u kobiet: niskie dawki **estrogenu** (pigułka lub plaster) stymulują wzrastanie, rozwój piersi, popęd płciowy (libido) i rozwój kobiecego wyglądu; estrogen skojarzony z progesteronem powoduje wystąpienie regularnych miesiączek (krwawień), ale nie wpływa na płodność.

Po rozpoczęciu leczenia dalsze **dawki leku są zwiększane stopniowo**, co może okazać się frustrujące dla niektórych pacjentów, którzy oczekują szybkich rezultatów.
Jednak ważne jest żeby wiedzieć, że dzięki takiemu postępowaniu uzyskamy najlepszy wzrost (a także rozwój piersi u kobiet).

U niektórych osób z CHH dochodzi do wyleczenia po zastosowanym leczenia i zaczynają produkować prawidłowe ilości hormonów. Nazywamy to odwróceniem. Przyczyny tego nie są poznane i nie jesteśmy w stanie przewidzieć, który pacjent będzie miał taką odwracalność. **To odwrócenie nie zawsze jest trwałe. Dlatego ważne jest, aby być dokładnie monitorowanym przez lekarza.**

U około 1 na 10 pacjentów z CHH (10%) dochodzi do odwrócenia

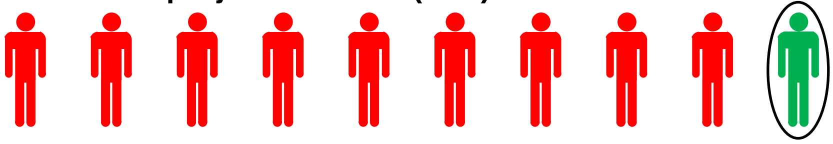

Czy pacjent z CHH może być płodnym?

Tak, **CHH jest uleczalną formą niepłodności.** Większość osób z CHH, choć nie wszyscy, może stać się płodnym po zastosowaniu **specjalistycznego leczenia hormonalnego.**

- leczenie specjalistyczne może być stosowane zarówno w formie zastrzyków hormonalnych wykonywanych kilka razy w tygodniu lub stosując przenośną pompę GnRH (podobną do tej stosowanej w cukrzycy).
- leczenie stymulujące płodność wymaga opieki doświadczonych specjalistów (**endokrynologów ds. rozrodu**)
- czasem (ale nie zawsze) konieczny jest wspomagany rozród, taki jak zapłodnienie *in vitro* (IVF).

Płodność: zwykle 3 na 4 pacjentów (75%) staje się płodnym

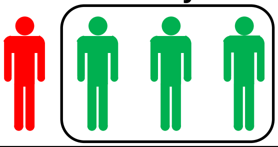

mężczyźni: proces produkcji nasienia może potrwać do 2 lat

kobiety: niektóre pacjentki stają się płodne po kilku miesiącach

Czy istnieje jakieś ryzyko, jeśli CHH nie jest leczony?

Chociaż testosteron i estrogen nie są istotnymi dla życia hormonami to ich brak (lub niedobór) może mieć poważny wpływ na **zdrowie, funkcję seksualną i jakość życia.**

U mężczyzn i kobiet:

- zwiększone ryzyko **obniżonej gęstości kostnej (osteoporoza)** – to oznacza, że kości są słabe i bardziej podatne na złamania. Jeśli CHH nie jest leczony, wówczas osteoporoza może wystąpić w każdym wieku. Niektórzy pacjenci z CHH wymagają dodatkowego leczenia z powodu osteoporozy.
- **obniżona funkcja seksualna i pożądanie** są wynikiem niskich stężeń hormonów
- **zmęczenie, gorsze samopoczucie oraz depresja** są powszechne u nieleczonych pacjentów lub po zaprzestaniu leczenia

Mężczyźni, którzy nie są leczeni, są także bardziej narażeni na wystąpienie zaburzeń metabolicznych, takich jak stan przedcukrzycowy czy cukrzyca. **Ryzyko to można zmniejszyć kontynuując leczenie.**

Punkty kluczowe:

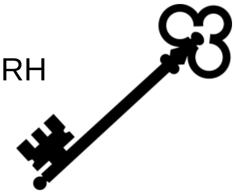

- CHH jest rzadkim schorzeniem spowodowanym niedoborem GnRH
- CHH jest przyczyną braku dojrzewania i niepłodności
- CHH nie powinien wpłynąć na skrócenia twojego życia
- CHH jest trudno rozpoznać
- wiele osób nie ma postawionego rozpoznania aż do późnych lat młodzieńczych czy nawet wczesnego okresu dorosłości
- w przeciwieństwie do wielu rzadkich chorób dostępne jest leczenie CHH
- pacjent wymaga długiego okresu leczenia i regularnych wizyt u lekarza
- rozmawiaj ze swoim lekarzem o sposobach leczenia aby zdecydować, który rodzaj leczenia jest dla ciebie najlepszy
- zaprzestanie leczenia ma negatywne konsekwencje dla twojego zdrowia i dobrego samopoczucia
- płodność jest możliwa w większości przypadków stosując specjalistyczne leczenie hormonalne
- możesz przekazać CHH potomstwu stąd rekomendowane jest poradnictwo genetyczne
- CHH jest trudnym problemem psychologicznym dla niektórych osób
- możesz otrzymać wsparcie od swoich lekarzy i grup wsparcia pacjentów

Przydatne odniesienia:

http://www.nature.com/nrendo/journal/v11/n9/full/nrendo.2015.112.html

Przydatne witryny internetowe:

http://www.gnrhnetwork.eu/hhn_home/hhn-patients_familles_gnrh_deficiency_kallmann_syndrome/hhn-onlinesupportgroups.htm
https://www.rareconnect.org/en/community/kallmann-syndrome
https://www.facebook.com/KallmannSyndrome/

Português
(Portuguese)

O que precisa saber acerca do hipogonadismo hipogonadotrófico congénito (HHC) e do síndrome de Kallmann (SK)

O que é o HHC?

O HHC é uma doença causada por uma **deficiência da hormona GnRH** (gonadotropin releasing hormone – hormona libertadora das gonadotrofinas). Quando ocorre conjuntamente com uma falta do sentido do olfato (anosmia), essa doença é conhecida como síndrome olfato-genital ou S. de Kallmann (SK). Ambas as situações são diagnosticadas e tratadas de forma semelhante.

O que é que faz a hormona GnRH?

A GnRH é uma hormona essencial para o desenvolvimento sexual, da puberdade e da fertilidade.

1. A GnRH é libertada no hipotálamo, uma área do cérebro que controla várias funções no nosso corpo.
2. A GnRH atua diretamente na hipófise (pituitária) (uma glândula do tamanho de uma ervilha situada junto ao hipotálamo) produzindo a libertação de duas hormonas: a hormona luteinizante (LH) e a hormona folículo-estimulante (FSH).
3. A LH e a FSH são importantes para o desenvolvimento da puberdade e para a fertilidade.
   - <u>nos homens</u>: a LH e a FSH estimulam os testículos a produzirem testosterona e espermatozoides.
   - <u>nas mulheres</u>: a LH e a FSH estimulam os ovários a produzirem estrogénios e progesterona, que são importantes para a fertilidade.

O que é que acontece se não houver GnRH?

A deficiência completa ou parcial de GnRH resulta na falta, ou valores muito baixos, de LH e FSH, que por sua vez resulta no **não desenvolvimento da puberdade e em infertilidade** (ver figura abaixo). Isto significa que:

- <u>nos homens:</u> 1) os testículos não crescem, 2) a testosterona não é produzida nas quantidades normais, e 3) os espermatozoides não são produzidos.
- <u>nas mulheres:</u> 1) as células (ovócitos) necessárias para a fertilização estão presentes mas não se desenvolvem e não são libertadas (ovulação), 2) Os estrogénios e progesterona não são produzidos, e 3) não ocorrem períodos menstruais.

Sinais da não ocorrência de puberdade em adolescentes e adultos jovens

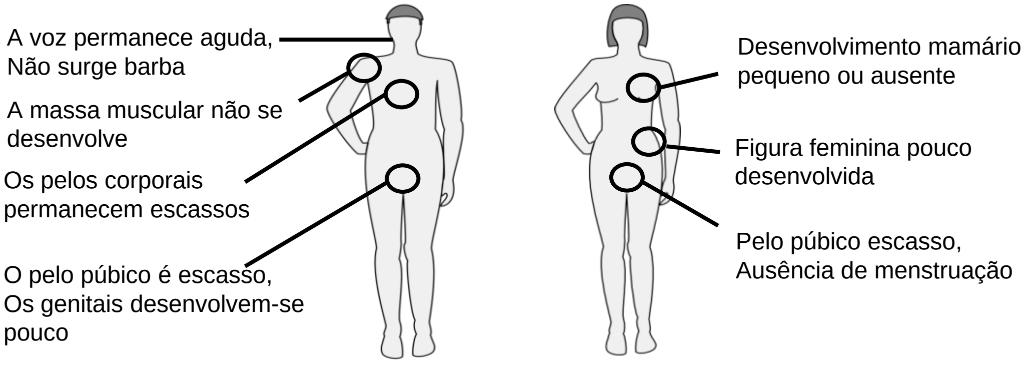

Estes problemas estão geralmente presentes na altura do nascimento (por isso esta doença é chamada congénita), mas o diagnóstico frequentemente só é feito na adolescência ou quando se é já um adulto jovem e a puberdade não começou. **É muito importante saber que esta condição tem tratamento. Na maioria dos casos é necessário fazer o tratamento de substituição com hormonas e vigilância clínica durante toda a vida.**

2017: www.gnrhnetwork.eu / andrew.dwyer@chuv.ch

Quem é afetado pelo HHC e como se diagnostica?

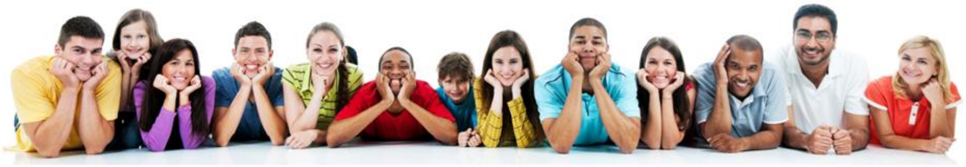

Quem pode ser afetado por HHC?

Tanto homens como mulheres podem ser afetados. O HHC é mais frequentemente diagnosticado em homens, mas não se sabe bem porquê. Poderá ser porque muitas mulheres vão ao Ginecologista e são tratadas com contracetivos orais («pílula») para regular os períodos menstruais sem antes serem devidamente diagnosticadas. Existem casos de pacientes que nunca foram diagnosticados e nunca consultaram um especialista em deficiência de GnRH.

Quantas pessoas afetadas, existem?

O HHC é raro, pelo que é difícil indicar uma estimativa exata. Acredita-se que esta doença ocorre em 1 em cada 4 000 a 10 000 pessoas. Isto significa que o total de pessoas com HHC na Europa (cerca de 74 000) daria para encher o Estádio da Luz, em Lisboa.

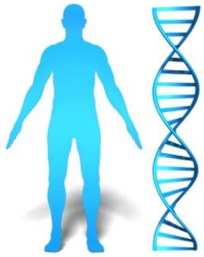

O HHC é hereditário? O HHC pode ser hereditário. Isto significa que **pode ser transmitido** ao longo das gerações em algumas famílias. Em muitos casos, porém, não há uma história familiar evidente. Uma causa genética pode ser identificada em cerca de metade dos casos (50%). No entanto, é provável que a investigação nesta área venha a descobrir mais causas genéticas de HHC. Na maioria dos casos, é difícil prever com segurança se um paciente vai transmitir o HHC aos seus filhos.

Metade dos doentes tem uma causa genética conhecida e outra metade não tem.

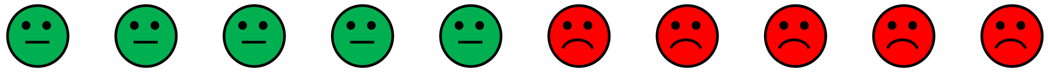

O HHC é uma doença complicada porque em alguns casos pode ser causada por uma combinação de duas ou mais alterações (mutações) em genes diferentes. Isto torna **difícil prever se o HHC será transmitido aos filhos**. Para um dos genes, *ANOS1* (*KAL1*), a previsão é mais simples. É necessário que se continue a investigar esta doença para a compreender melhor e melhorar o diagnóstico.

Como é que se diagnostica o HHC?

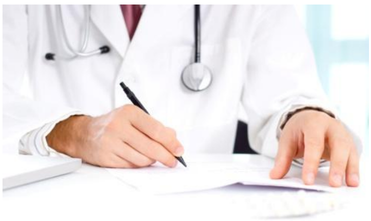

O HHC é difícil de diagnosticar. O tempo «normal» para ocorrer a puberdade é muito variável, pelo que é muito difícil decidir se a puberdade está atrasada (e no entanto normal), ou se se trata de um caso de HHC e a precisar de tratamento. São necessárias consultas regulares para seguir a evolução da puberdade. Deve-se perguntar sempre aos pacientes se têm alguma deficiência do olfato. Os sinais que indicam a existência de HHC são:

- **Nos rapazes –** se não houver sinais de puberdade pelos 16 anos e houver níveis de testosterona, LH e FSH baixos.
- **Nas raparigas –** se não houver desenvolvimento mamário pelos 14-15 anos e não começarem a ter menstruações pelos 16-17 anos, e níveis de estrogénios, LH e FSH baixos.
- **Outros testes são necessários** para ter a certeza de que não há outros problemas que possam estar a causar os níveis baixos das hormonas. Esses testes incluem análises sanguíneas e exames de imagem (radiografias, ecografias e ressonâncias magnéticas).

Há sinais precoces a que deva estar atento?

Na maior parte dos casos, não há sinais de HHC até se verificar que não ocorreu a puberdade.

Algumas crianças do sexo masculino apresentam sinais que sugerem o HHC: **micropénis ou testículos que não desceram** para as bolsas escrotais (criptorquidia).

O micropenis pode ser tratado na infância ou antes da puberdade através de um tratamento hormonal. Os testículos não descidos devem ser tratados cirurgicamente o mais cedo possível, de preferência no primeiro ano de vida, para não afetar a fertilidade no futuro.
Há ainda outros sinais não diretamente relacionados com o HHC que ocorrem em alguns doentes, mas não em todos**.**

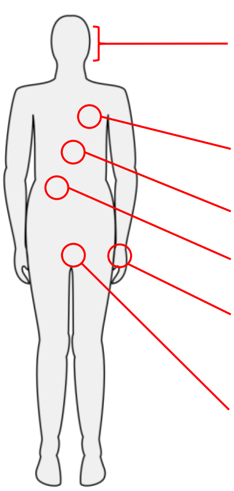

Como é que a falta da puberdade afeta uma pessoa?

Não ter o desenvolvimento pubertário ao mesmo tempo das pessoas da mesma idade **pode ser muito stressante e às vezes traumatizante**. Este pode ser um grande problema para alguns doentes com HHC. O impacto que isto causa pode em alguns casos ser grave, embora seja variável de pessoa para pessoa. Os anos da adolescência são difíceis mesmo para as pessoas que desenvolvem a puberdade no tempo normal, mas aquelas que não iniciam a puberdade e que são deixadas de lado pelo seu grupo de companheiros podem ter potenciais consequências que incluem:

- baixa autoestima, falta de confiança
- timidez, dificuldade em interagir com outros
- ansiedade, depressão
- fraca autoimagem corporal

A falta de olfato poderá afetar o seu sentido do paladar e de apreciar a comida. Algumas pessoas poderão não detetar o cheiro a gás ou de comida estragada. Algumas poderão ficar preocupadas com o seu odor corporal.

O que pode fazer?

No geral, estas poderão ser resolvidas. O aconselhamento e tratamento psicológico pode ser útil.

1. **fale com o seu profissional de saúde** – eles poderão não se aperceber que está a passar momentos difíceis. Poderão ajudá-lo a encontrar aconselhamento e apoio.
2. **comunique com outros pacientes –** os grupos online (Facebook, RareConnect.org) e grupos de apoio de cara-a-cara poderão ser uma boa ajuda. Estes são locais onde os pacientes poderão conversar sobre aspetos que são importantes para eles. Outros pacientes compreendem como é viver com HHC no dia-a-dia e poderão dar conselhos práticos e apoio.

O que deve fazer para se manter saudável?

Ter HHC não implica menos anos de vida. Algumas recomendações para se manter saudável:

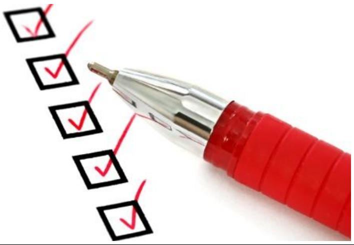

- manter consultas médicas regulares
- tomar a medicação da forma indicada
- manter uma dieta saudável
- fazer exercício físico regular
- não fumar

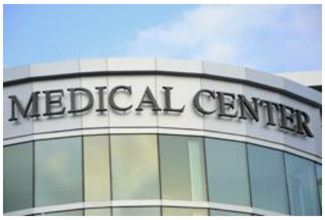

O HHC tem cura?

De momento, **não existe cura para o HHC**. É muito difícil curar uma doença congénita (genética). Estão a decorrer investigações para tentar repor a produção de GnRH pelo hipotálamo. As investigações estão ainda numa fase muito inicial mas espera-se que isto seja possível no futuro.

Existem tratamentos para o HHC?

Existem tratamentos para desenvolver os sinais exteriores da puberdade, tais como o crescimento da barba nos homens e o desenvolvimento mamário nas mulheres. Existem tratamentos hormonais especiais para ajudar a desenvolver a fertilidade na maioria dos casos.

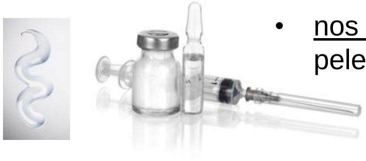

- <u>nos homens</u>: o tratamento com **testosterona** (na forma de gel aplicado na pele ou injeções) é o mais usado para desenvolver os sinais de puberdade. O tratamento induz o crescimento, uma voz mais masculina, o desenvolvimento da barba e do pénis, o aumento do desejo (líbido) e função sexual – mas não a fertilidade.

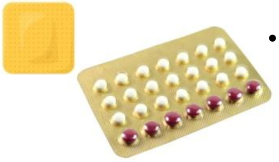

- <u>nas mulheres</u>: os **estrogénios** em baixas doses (comprimidos ou adesivos) induzem o crescimento e ajudam o desenvolvimento mamário, o aumento do desejo sexual (líbido) e um corpo mais feminino, e combinado com progesterona, causam períodos menstruais regulares – mas não a fertilidade.

Quando o tratamento é iniciado, a **dose é ajustada gradualmente**. Isto poderá ser frustrante para algumas pessoas que esperam um resultado mais rápido. No entanto, isto é importante para maximizar o crescimento (e o desenvolvimento mamário nas mulheres).

Algumas pessoas com HHC recuperam após o tratamento e podem produzir níveis hormonais normais. A isto chama-se reversão. As razões para isto não são conhecidas e de momento não é possível prever quem terá esta reversão. **Esta recuperação nem sempre é duradoura, pelo que é importante continuar a ter acompanhamento médico.**

Cerca de 1 em cada 10 (10%) casos terá uma reversão

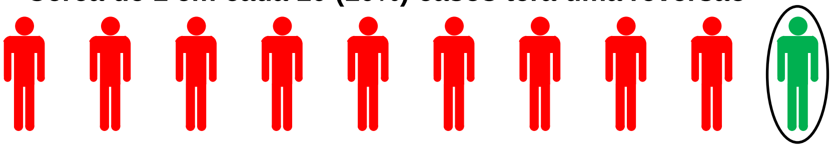

Uma pessoa com HHC pode vir a ser fértil?

Sim, o **HHC é uma forma de infertilidade tratável.** A maioria (mas não todas) das pessoas com HCC pode tornar-se fértil através de **tratamentos hormonais especializados.**

- o tratamento especializado poderá incluir injeções de hormonas, várias vezes por semana, ou o uso de uma bomba portátil de GnRH (semelhante às bombas usadas por alguns diabéticos).
- o tratamento deverá ser feito por especialistas experientes (**em endocrinologia e reprodução**).
- por vezes (mas nem sempre) é necessário recorrer à reprodução medicamente assistida, como a fertilização *in vitro*.

Fertilidade: Em termos gerais, 3 em cada 4 casos (75%) terão sucesso

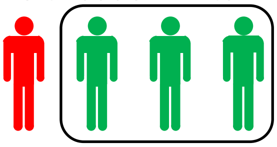

<u>homens</u>: podem levar 2 anos a desenvolver espermatozoides

<u>mulheres</u>: por vezes a fertilidade é conseguida em poucos meses

2017: www.gnrhnetwork.eu / andrew.dwyer@chuv.ch

Existem riscos em não tratar o HHC?

Apesar da testosterona e estrogénios não serem hormonas indispensáveis para a vida, a sua ausência (ou deficiência) pode prejudicar seriamente a **saúde, função sexual e qualidade de vida.**

Em homens e mulheres

- aumento do risco de **baixa densidade óssea (osteoporose)** –isto significa que os ossos são frágeis e têm mais risco de sofrerem fraturas. Se o HHC não for tratado, a osteoporose pode ocorrer em qualquer idade. Algumas pessoas com HHC podem precisar de tratamentos adicionais para a osteoporose.
- **diminuição da função e desejo sexual** é o resultado dos baixos níveis hormonais.
- **cansaço e depressão** são frequentes em pessoas não tratadas.

Homens sem tratamento têm também um maior risco de problemas metabólicos, tais como pré-diabetes ou diabetes. **Estes riscos podem ser reduzidos mantendo o tratamento**.

Pontos importantes:

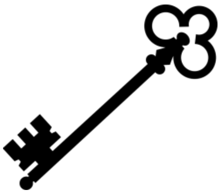

- HHC é uma condição rara causada por deficiência de GnRH
- HHC resulta em ausência de puberdade e infertilidade
- HHC não reduz o tempo da sua vida
- HHC é difícil de diagnosticar
- muitas pessoas só são diagnosticadas tarde na adolescência ou quando adultos
- ao contrário de outras doenças raras, existem tratamentos disponíveis
- precisará de tratamento ao longo de toda a sua vida e de consultas regulares com o seu médico
- fale com o seu médico sobre as opções de tratamento mais apropriadas para si
- a suspensão do tratamento pode ter consequências negativas para a sua saúde
- a fertilidade é muitas vezes possível através de tratamentos hormonais especiais
- o HHC pode ser transmitido aos seus descendentes, pelo que poderá precisar de aconselhamento genético
- o HHC é psicologicamente difícil para algumas pessoas
- poderá encontrar apoio nos profissionais de saúde e em grupos de doentes

Referência útil

http://www.nature.com/nrendo/journal/v11/n9/full/nrendo.2015.112.html

Websites úteis

http://www.gnrhnetwork.eu/hhn_home/hhn-patients_familles_gnrh_deficiency_kallmann_syndrome/hhn-onlinesupportgroups.htm

https://www.rareconnect.org/en/community/kallmann-syndrome

https://www.facebook.com/KallmannSyndrome/

Ce trebuie să știți despre hipogonadismul hipogonadotrop congenital (CHH) și sindromul Kallmann (KS)

Ce este CHH?

CHH este produs de deficiența **GnRH (gonadotropin releasing hormone)**. Când se însoțește de absența simțului mirosului (anosmie) este cunoscut ca sindrom olfacto-genital sau sindrom Kallmann (KS). Toate formele sunt diagnosticate și tratate în același fel.

Care este funcția GnRH?

GnRH este un hormon esențial pentru dezvoltarea sexuală, pubertate și fertilitate.

1. GnRH este secretat de hipotalamus, o mică zonă de la baza creierului care controlează mai multe funcții ale organismului.
2. GnRH actionează direct asupra glandei hipofize (o mică glandă de la baza creierului) care va elibera: hormonul luteinizant (LH) și hormonul foliculo-stimulator (FSH).
3. LH și FSH sunt esențiali pentru pubertate și pentru fertilitate.
   - la bărbați : LH & FSH stimulează testiculii să producă testosteron și spermă.
   - la femei: LH & FSH stimulează ovarele să producă estrogeni, progesteron și determină ovulația esențială pentru fertilitate.

Ce se întâmplă dacă nu exită GnRH?

Deficitul parțial sau complect de GnRH determină absența partială sau complectă a secreției de LH și FSH care se va traduce în : absența pubertății și infertilitate (vezi figura de mai jos). Asta înseamnă că:

- La bărbați: 1) testiculii nu cresc, 2) nu se secretă testosteron în cantitate suficientă, 3) nu se produce spermă.
- La femei: 1) deși sunt prezente în ovare, ovulele nu se dezvoltă și nu sunt eliberate (ovulație), 2) estrogenii și progesteronul nu sunt secretați, sau sunt secretați în cantitate insuficientă 3) nu apare sângerarea menstruală.

Semne de nedezvoltare pubertară la adolescenți și adulți

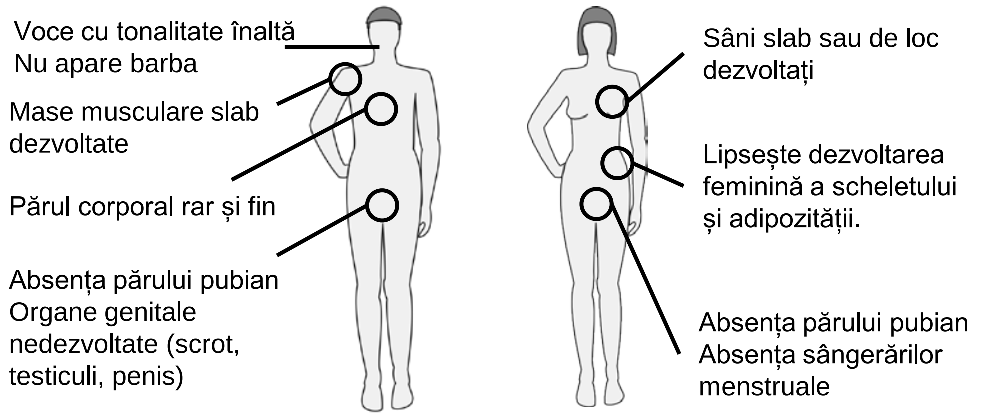

Aceste anomalii sunt prezente încă de la naștere (congenitale) dar diagnosticul este frecvent făcut la adolescent sau adult, când manifestarea clinică devine evidentă prin neapariția pubertății. **Important, aceasta anomalie este tratabilă! In majoritatea cazurilor este necesar tratament hormonal pentru toată viața și monitorizare medicală.**

2017: www.gnrhnetwork.eu / andrew.dwyer@chuv.ch

Cine este afectat de CHH și cum se face diagnosticul?

Cine este afectat de CHH?

Atât bărbații cât și femeile pot fi afectați, dar CHH este mai frecvent diagnosticat la bărbați din motive necunoscute. O explicație ar fi aceea că femeile se adresează ginecologului care instituie tratament cu tablete contraceptive, sub care ciclurile vor surveni regulat, fără a fi precizat diagnosticul și fără a fi investigate pentru deficitul de GnRH.

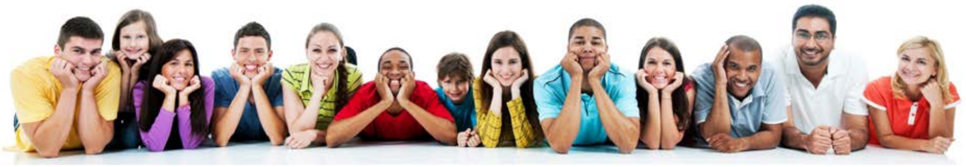

Cât de mulți oameni sunt afectați?

CHH este o boală rară, așa că estimarea precisă este dificilă. In prezent se estimează că afectează între 1/4000 si 1/10000 oameni. Asta înseamnă că la populația Europei, numărul pacienților este în jur de 74000, cam cât capacitatea stadionului olimpic din Berlin.

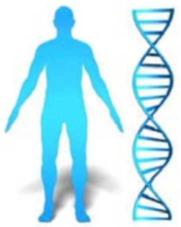

CHH este o boală moștenită?

CHH poate fi ereditară (moștenită), adică poate fi transmisă în familie de-a lungul generațiilor. În multe cazuri însă nu există o istorie familială. O cauză genetică poate fi identificată la jumătate dintre pacienți, dar cercetările continuă pentru a identifica și alte anomalii genetice care produc boala. In majoritatea cazurilor, este dificil de precizat cu acuratețe daca pacienții cu CHH vor transmite boala la copiii lor.

In prezent, doar la jumătate din pacienți cauza genetică poate fi identificată

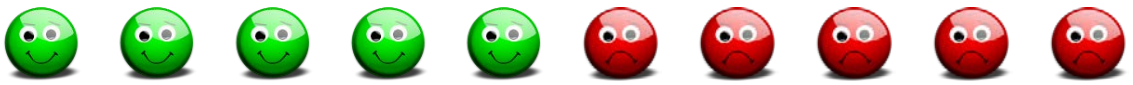

CHH este o boală complicată fiindcă, în unele cazuri poate fi produsă de mai multe mutații în gene diferite. Din această cauză este **greu de precizat dacă boala va fi transmisă copiilor.** In cazul genei ANOS1 (*KAL1*) transmiterea la descendenți este ușor predictibilă. Este necesară continuarea cercetărilor pentru a înțelege mai bine boala și a îmbunătăți diagnosticul.

Cum este diagnostiocat CHH ?

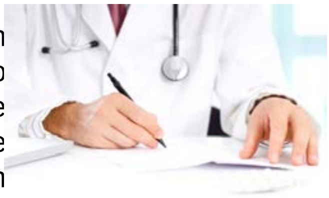

CHH este greu de diagnosticat . Există o mare variabilitate în debutul pubertății normale, de aceea este dificil de diferențiat o pubertate întârziată (dar normală) de CHH, care va avea nevoie de tratament pentru inducerea pubertății. De aceea, în caz de dubiu, este necesară urmărirea progresiei pubertare prin consultații regulate. Pacienții vor fi întotdeauna interogați asupra simțului mirosului. Semne clinice care sugerează CHH sunt:

• **la băieți –** absența semnelor pubertare până la 16 ani, testosteron, FSH, LH scăzuți indică CHH.
• **la fete -** absența dezvoltării mamare până la 14-15 ani și a menstruațiilor până la 16-17 ani, cu estradiol, LH & FSH scăzuți indică CHH.

• **Sunt necesare și alte teste** care să excludă alte cauze de insuficiență hormonală.: teste de sânge, imagistică (Rx, ecografie, RMN).

Există semne precoce ale bolii?

La majoritatea pacienților nu există semne de CHH înainte de vârsta pubertară când se constată absența semnelor de pubertate.

Unii băieți cu CHH prezintă semne sugestive din copilărie: **micropenis si testiculi nedescinși în scrot (cri**ptorchidism).

Micropenisul poate fi tratat în copilărie cu ajutorul hormonilor. Criptorhidia trebuie tratată în primul an de viață (hormonal sau chirurgical) pentru a conserva fertilitatea testiculilor.

Există și alte semne, care nu sunt datorate CHH, dar care se pot asocia la unii pacienti, nu la toti.

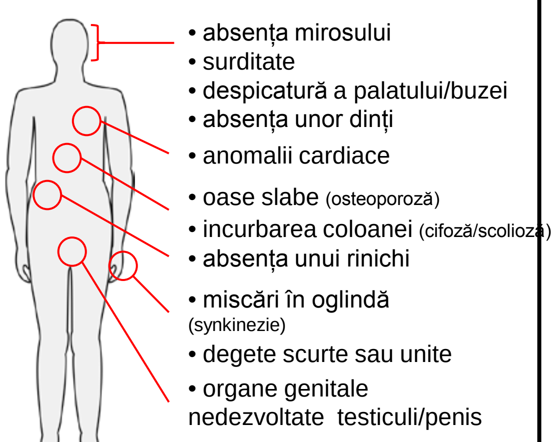

Alte anomalii posibile

In ce fel este afectată o persoană de absența pubertății?

Nedezvoltarea caracterelor sexuale secundare în același timp cu tinerii de aceeași vârstă poate fi foarte stresant și traumatizant. Poate fi o problemă majoră pentru unii pacienți cu CHH. Impactul poate fi sever în unele cazuri, deși variază de la o persoană la alta. Anii adolescenței pot fi dificili și pentru adolescenții cu pubertate normală, în timp ce nedezvoltarea pubertară, marginalizarea în grupul de colegi, poate genera pentru cei afectați de CHH probleme psihologice printre care:

- lipsa încrederii in sine
- timiditate, dificultate în relațiile cu colegii
- anxietate, depresie
- imagine corporală negativă

Absența simțului mirosului afectează capacitatea de a percepe gustul și scade plăcerea de a mânca. Cei afectați nu percep mirosul gazului metan sau gustul mâncării alterate, aspecte potențial periculoase; unii pot fi stresați de mirosul propriului corp.

Ce poți să faci?

In mare, aceste probleme psihologice pot fi depășite dar uneori este nevoie de consiliere psihologică și/sau psihoterapie.

1. **Vorbește cu medicul tău despre aceste probleme** – este posibil ca el să nu-și dea seama de dificultățile prin care treci. Te va ajuta sa găsești consiliere și ajutor.

2. **Ia legătura cu alți pacienți -** cu ajutorul grupurilor on-line (Facebook, RareConnect.org) sau întâlniri ale grupurilor de suport care pot fi foarte utile. Sunt locuri în care pacienții vorbesc despre aspecte importante pentru ei în contextul CHH. Pacienții știu din proprie experiență cum e să trăiești zi de zi cu CHH și pot oferi soluții și sfaturi utile.

Ce să faci ca să fii sănătos?

Să ai CHH nu îți va scurta durata vieții. Există lucruri pe care le poți face ca să fii sănătos:

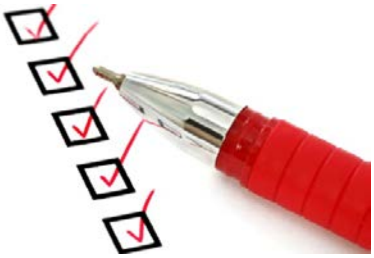

- Consultă cu regularitate medicul!
- Ia-ți tratamentul corect, în dozele prescrise!
- Mănâncă sănătos!
- Fă exercițiu fizic regulat!
- Nu fuma!

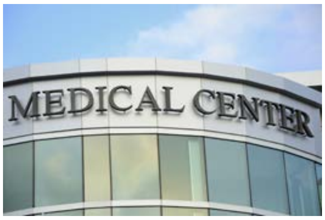

Poate fi vindecat CHH?

În mod curent **nu există vindecare pentru CHH**. Este foarte dificil să vindeci o boală congenitală, genetică. Există cercetări în curs privind posibilitatea de a restaura secreția hipotalamică de GnRH, dar aceste cercetări sunt încă la început; se speră că acest lucru va fi posibil în viitor.

Există tratamente disponibile pentru CHH?

Există tratamente capabile sa dezvolte semnele de sexualizare pubertară – precum: creșterea bărbii la băieți sau dezvoltarea sânilor la fete. De asemenea, sunt disponibile tratamente care permit, în majoritatea cazurilor, dobândirea fertilității.

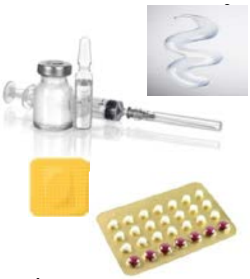

La băieți: **testosteronul** (gel aplicat pe piele sau injecții) este tratamentul utilizat pentru a obține dezvoltarea pubertară. Tratamentul stimulează: creșterea, modificarea vocii, creșterea bărbii, dezvoltarea scrotului și a penisului precum și o funcție sexuală normală – dar nu și fertilitatea.

La fete: doze mici de estrogeni (tablete, gel, patch) stimulează creșterea staturală, dezvoltarea sânilor și a aspectului feminin, iar în combinație ciclică cu progesteronul va produce sângerarea menstruală lunară – dar nu și fertilitate.

Ajustarea dozelor trebuie făcută progresiv, începând cu doze mici, ceea ce poate fi frustrant pentru pacienți (care așteaptă o transformare rapidă) dar este important pentru a obține o creștere staturală corespunzătoare și o dezvoltare mamară corespunzătoare la femei.

Unii pacienți cu CHH se vindecă după o perioadă de tratament și pot produce hormonii în cantități normale. Se numește reversibilitate. Cauzele nu sunt înțelese și nu se poate prezice care pacient va avea reversibilitate. **Acesta reversibilitate este uneori temporară, așa că pacientul trebuie atent monitorizat de medicul specialist.**

In 1 din 10 cazuri (10%) CHH este reversibilă

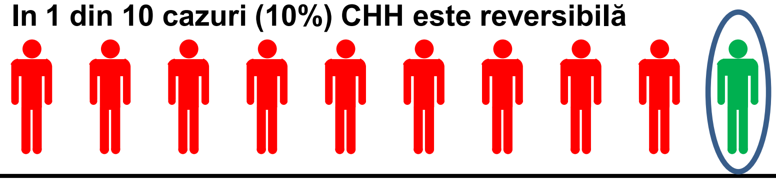

Poate o persoană cu CHH să devină fertilă?

Da, **CHH este o formă tratabilă de infertilitate.** Majoritatea – dar nu toți – pacienții cu CHH pot deveni fertili cu ajutorul unui **tratament hormonal specializat.**

- Tratamentul specific constă fie în injecții cu hormoni, de mai multe ori pe săptămână, fie în pompă de GnRH (asemănătoare pompelor de insulină folosite în tratamentul diabetului).
- Tratamentul de fertilitate este asigurat de specialiști cu experiență în endocrinologia reproducerii.
- Uneori sunt necesare tehnici de fertilizare asistată, de exemplu *fertilizarea in vitro* (FIV).

Fertilitate: 3 din 4 pacienți (75%) reușesc sa devină fertili

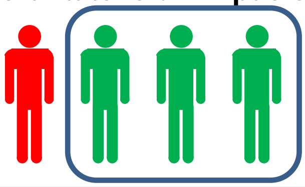

- bărbat: poate dura pană la doi ani să se obțină sperma corespunzătoare
- femeie: în unele cazuri fertilitatea este obținută în câteva luni.

Care sunt riscurile dacă CHH nu este tratat?
În timp ce testosteronul și estradiolul nu sunt hormoni esențiali pentru supraviețuire, absența (sau insuficiența) lor are un impact negativ asupra: **sănătății, funcției sexuale și calității vieții.**

La bărbat și femeie:

- Risc crescut de **densitate minerală osoasă scăzută (osteoporoză)** – care înseamnă o fragilitate mai mare a oaselor și un risc mai mare de fractură. Dacă CHH nu este tratat, osteoporoza poate surveni la orice vârstă. Unii pacienți cu CHH, chiar tratați hormonal corect, necesită tratament suplimentar pentru osteoporoză.
- **Dorința si funcția sexuală scăzute** sunt un rezultat al nivelului hormonal scăzut.
- **Oboseala, descurajarea, depresia** sunt simptome frecvente la pacienții netratați.

Bărbații netratați au un risc mai mare pentru tulburări metabolice (pre-diabet sau diabet)
Aceste riscuri pot fi scăzute prin urmarea constantă a tratamentului.

Elemente cheie:

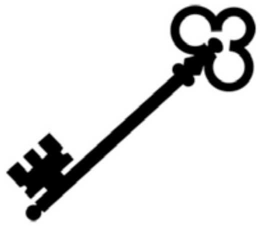

- CHH este o boală rară produsă de deficitul de GnRH.
- CHH determină absența pubertății și infertilitate.
- CHH nu trebuie să scurteze durata vieții.
- CHH este dificil de diagnosticat.
- Mulți pacienți sunt diagnosticați doar la vârsta adolescentei sau de adult.
- Spre deosebire de multe alte boli rare, pentru CHH există tratament eficace.
- Aveți nevoie de tratament pentru toată viața și de controale medicale periodice.
- Discutați cu medicul dumneavoastră endocrinolog despre opțiunile de tratament pentru a-l alege pe cel mai bun pentru dumneavoastră.
- Oprirea tratamentului are consecințe negative asupra sănătății si a stării de bine.
- Fertilitatea este posibilă, în majoritatea cazurilor, cu tratament hormonal special.
- Puteți transmite CHH copiilor dumneavoastră, de aceea consilierea genetică este recomandată.
- Pentru unii pacienți, CHH este dificil din punct de vedere psihologic.
- Veți găsi sprijin din partea medicilor curanți și a grupurilor de pacienți.

Referințe utile:
http://www.nature.com/nrendo/journal/v11/n9/full/nrendo.2015.112.html
Website-uri utile:
http://www.gnrhnetwork.eu/hhn_home/hhn-patients_familles_gnrh_deficiency_kallmann_syndrome/hhn-onlinesupportgroups.htm
https://www.rareconnect.org/en/community/kallmann-syndrome
https://www.facebook.com/KallmannSyndrome/

Что вам нужно знать о врождённом гипогонадотропном гипогонадизме (ВГГ) и синдроме Кальмана (Каллмана)?

Что такое ВГГ?

ВГГ возникает **из-за дефицита гонадотропин-рилизинг-гормона (второе название - гонадолиберин) (ГнРГ).** Если при этом пациент не чувствует запахи (такое состояние называется «аносмия»), то в таком случае диагностируют офтальмогенитальный синдром Кальмана (Каллмана). Диагностика и лечение этих заболеваний не отличаются.

Какую роль играет ГнРГ?

ГнРГ – это гормон, необходимый для полового созревания и фертильности.

1. Этот гормон вырабатывается в гипоталамусе – участке мозга, который регулирует несколько функций организма.
2. ГнРГ стимулирует гипофиз (гипофиз – это железа размером с горошину, расположенная в головном мозге), что приводит к секреции двух гормонов: фолликулостимулирующего гормона (ФСГ) и лютеинизирующего гормона (ЛГ).
3. ЛГ и ФСГ также необходимы для нормального полового созревания и фертильности.
   • <u>У мужчин</u>: ЛГ и ФСГ стимулируют яички, которые вырабатывают тестостерон и сперму.
   • <u>У женщин</u>: ЛГ и ФСГ стимулируют яичники, которые вырабатывают эстроген и прогестерон.

Эти гормоны влияют на фертильность.

Что происходит в организме при отсутствии ГнРГ?

Если в организме не хватает ГнРГ, то уровень ЛГ И ФСГ также очень низкий, или же эти гормоны вообще отсутствуют. В результате, **полове созревание не наступает и развивается бесплодие** (см. рисунок). А это значит, что:

- <u>• у мужчин: </u>1) не развиваются яички, 2) не вырабатывается тестостерон в нужных количествах, 3) отсутствует сперма
- <u>у женщин:</u> 1) хотя и имеются яйцеклетки, они не развиваются и, соответсвтенно, отсутствует овуляция, 2) эстроген и прогестерон не вырабатываются, 3) отсутствуют менструации.

Признаки отсутствия полового созревания у подростков и молодых людей

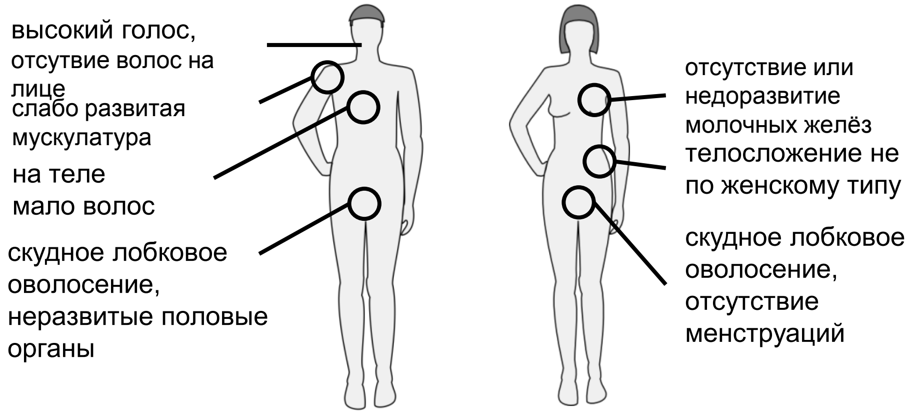

Указанные проблемы почти всегда наблюдаются с самого рождения (их называют «врождёнными»). Однако само заболевание обычно диагностируют в подростковом или раннем юношестком возрасте, когда у пациента не наступает половое созревание. **Помните, это заболевание можно лечить. В большинстве случаев, необходима заместительная гормональная терапия в течение всей жизни и сопутствующее лечение.**

Кто страдает ВГГ и как это заболевание диагностируют?

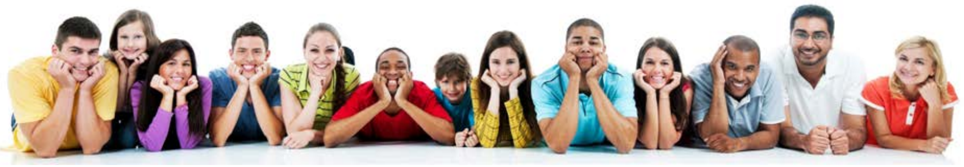

У кого диагностируют ВГГ?

Этим заболеванием страдают и мужчины, и женщины. ВГГ чаще встречается у мужчин. Причина этого неизвестна. Вполне вероятно, что женщины с ВГГ обращаются к гинекологам, но те лишь назначают противозачаточные препараты, чтобы нормализовать месячные, не поставив при этом точный диагноз. У некоторых пациентов ВГГ так никогда и не будет диагностирован, и они никогда не обратятся к врачу из-за дефицита ГнРГ.

Сколько человек страдает этим заболеванием?

ВГГ – это редкое заболевание, поэтому точное количество пациентов установить трудно. Считается, что ВГГ страдает 1 человек из 4000-10000. Это значит, что все пациенты с ВГГ в Европе (около 74000 человек) поместятся на Олимпийском стадионе в Берлине (Германия).

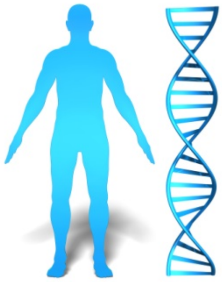

Передаётся ли заболевание по наследству? ВГГ может быть наследственным. Это значит, что он **может передаваться по наследству.** Хотя во многих случаях в семейном анамнезе это заболевание отсутствует. Примерно в половине случаев (50%) ВГГ вызывается генетическими факторами. В настоящее время проводятся научные исследования, которые должны дать больше информации о генетических факторах, вызывающих ВГГ. В большинстве случаев, врач не может с полной уверенностью и абсолютной точностью сказать, передадут ли пациенты с ВГГ это заболевание своим детям.

У половины пациентов наследственные причины ВГГ известны, у половины – нет

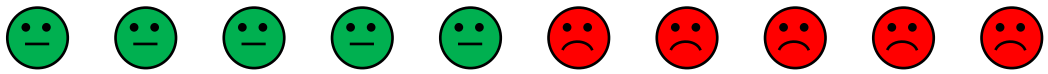

ВГГ – это сложное по своей структуре заболевание, поскольку его может вызывать сочетание двух и более изменений (мутаций) в различных генах. Поэтому **почти невозможно предсказать, унаследуют ли дети пациента ВГГ.** Чтобы лучше понять механизмы заболевания и усовершенствовать диагностические методы, необходимо провести дополнительные исследования.

Как диагностируют ВГГ?

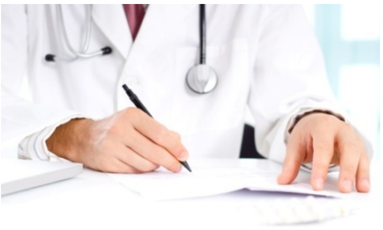

Диагностировать ВГГ непросто. Половое созревание может наступать в разном возрасте. Поэтому чрезвычайно трудно определить, имеет ли место задержка половго созревания (как один из вариантов нормы) <u>или</u> мы имеем дело с ВГГ, и в этом случае требуется лечение. Такие пациенты должны находится под наблюдением у эндокринолога, который будет следить за процессом полового созревания. На ВГГ могут указывать следующие симптомы:

- **мальчики** – отсутствие признаков полового созревания к 16 годам, при этом уровень тестостерона, ЛГ и ФСГ низкий.
- **девочки** – молочные железы к 14-15 годам неразвиты, а в возрасте 16-17 лет отсутствуют менструации, при этом уровень эстрогена, ЛГ и ФСГ низкий.
- Чтобы исключить другие причины низкого уровня гормонов, **необходимо провести дополнительные анализы и исследования**.

Есть ли какие-то ранние симптомы заболевания?
У многих пациентов какие-либо симптомы ВГГ отсутствуют до тех пор, пока не станет ясно, что половое созревание не наступает.

У некоторых мальчиков может быть **микропенис или неопущение яичка** (крипторхизм) – симптомы, указывающие на ВГГ.

Размеры пениса можно увеличить в младенчестве или в детстве с помощью гормонов. Неопущение яичка можно вылечить хирургическим путём в первый год жизни ребёнка, что позволит ему в будущем иметь детей.

Есть и другие симптомы, не связанные с ВГГ, которые проявляются лишь у некоторых пациентов.

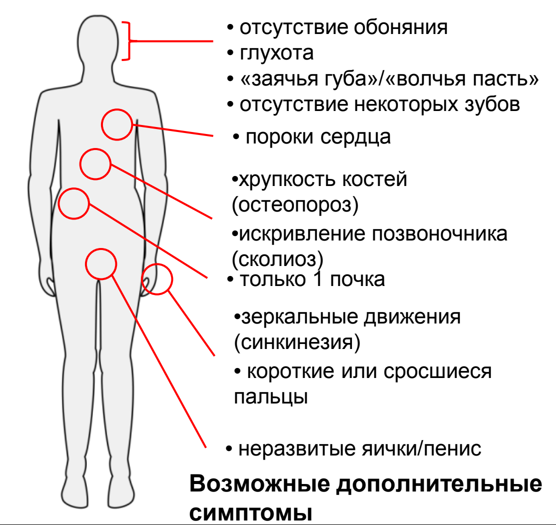

Возможные дополнительные симптомы

Как отсутствие полового созревания влияет на человека?
Когда все вокруг развиваются нормально, а у пациента с ВГГ полового созревания не происходит, это **может вызывать стресс и психологическую травму.** Для многих пациентов это большая трагедия. Переживания иногда могут быть очень сильными, хотя у разных людей они протекают по-разному. Подростковый возраст сам по себе – это трудный период жизни для людей с нормальным развитием. Но если у кого-то половое созревание не происходит, такой человек заметно отличается от сверстников и может наблюдаться следующее:

- низкая самооценка, неуверенность в себе
- застенчивость, трудности в общении со сверстниками
- тревога, депрессия
- недовольство своим телом

Что вы можете сделать?
В целом, указанные проблемы можно решить. Возможно, понадобится психологическая помощь.

1. **Поговорите с врачом.** Он может и не знать, как вам сейчас тяжело. Врач может порекомендовать вам соответствующего специалиста.
2. **Поговорите с другими пациентами.** Их можно найти в Интернете (Фейсбук, RareConnect.org). Существуют также различные группы поддержки. Существуют места, где пациенты могут обсудить важные для них вопросы. Другие пациенты понимают, что значит жить с ВГГ каждый день, они могут дать практические советы и поддержать.

Как вы можете сохранить здоровье?
ВГГ не укорачивает жизнь. Следующие рекомендации помогут вам сохранить здоровье

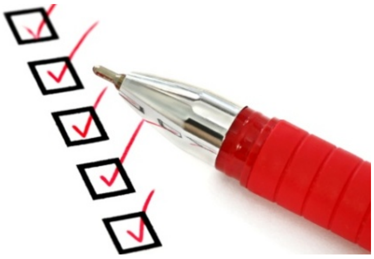

- выполнять назначения врача
- принимать назначенный препарат
- соблюдать режим питания
- регулярно заниматься спортом
- не курить

2017: www.gnrhnetwork.eu / andrew.dwyer@chuv.ch

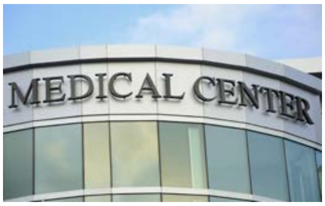

Можно ли вылечить ВГГ?

В настоящее время **лечения ВГГ не существует.** Наследственное (генетическое) заболевание лечить очень сложно. В настоящее время проводятся исследования на тему: можно ли восстановить синтез ГнРГ в гипоталамусе? Исследования находятся на начальных этапах, но учёные надеются, что в будущем это станет возможно.

Какие препараты используют при ВГГ?

Есть препараты, влияющие на такие внешние признаки полового созревания, как рост волос на лице у мужчин и развитие молочных желёз у женщин. В большинстве случаев с помощью определённых препаратов можно восстановить фертильность.

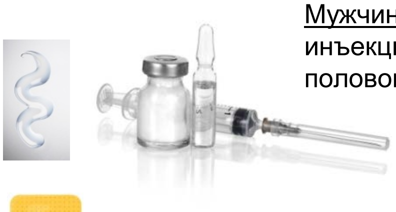

Мужчины: **Тестостерон** (гель для наружного применения или инъекции) – наиболее распространённый препарат для обеспечения полового созревания. Благодаря лечению, пациент становится сильнее, его голос грубеет, появляется растительность на лице, увеличивается пенис, появляется половое влечение (либидо) и нормализуется половая функция. При этом фертильность отсутствует.

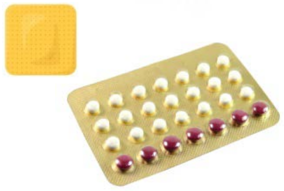

Женщины: низкие дозы эстрогена (пластырь или таблетки) приводят к увеличению и развитию молочной железы, появляется половое влечение (либидо), форма тела изменяется по женскому типу, а при добавлении прогестерона нормализуется менструальный цикл. При этом фертильность отсутствует.

После назначения лечения необходимо **подбирать дозу препарата.** Из-за этого, пациенты, ожидающие быстрых результатов, могут расстроиться. Однако важно добиться оптимального результата (в том числе, развития молочных желёз у женщин).

У некоторых пациентов все проявления болезни полностью исчезают, так что их организм производит достаточное количество гормнов. Это называется обратным развитием болезни. Почему так происходит, неизвестно, и спрогнозировать обратное развитие невозможно. **Такое выздоровление бывает временным. Поэтому необходимо врачебное наблюдение.**

Примерно у 1 из 10 пациентов с ВГГ (10%) отмечается обратное развитие заболевания

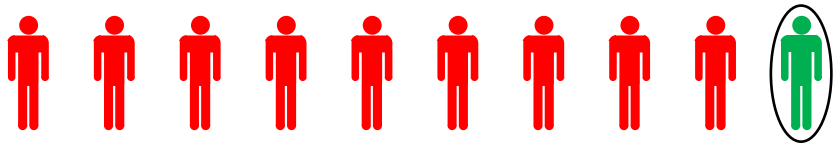

Могут ли у пациента с ВГГ быть дети?

Да, **бесплодие при ВГГ успешно лечится.** Большинство (но не все) пациенты с ВГГ, благодаря **приёму гормонов**, могут иметь детей.

- пациенты могут получать гормоны в виде инъекций несколько раз в неделю или с помощью ГнРГ-помпы (похожей на инсулиновую помпу для диабетиков).
- лечение бесплодия должно проводиться опытными специалистами (**репродуктивными эндокринологами**)
- иногда (но не всегда) проводят искусственное оплодотворение, например экстракорпоральное оплодотворение (ЭКО, «дети из пробирки»).

Фертильность: В целом, лечение успешно у 3 из 4 пациентов (75%)

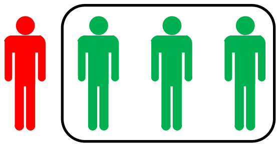

Мужчины: иногда требуется 2 года, чтобы организм начал вырабатывать сперму

Женщины: в некоторых случаях фертильность восстанавливается уже через несколько месяцев

А если не лечить ВГГ? Как это повлияет на пациента?

Хотя тестостерон и эстроген нельзя назвать жизненноважными гормонами, их отуствие (или нехватка) может отрицательно сказаться на **здоровье пациента, половой функции и качестве жизни.**

У мужчин и женщин

- высок риск **снижения минеральной плотности костей (остеопороза)**, то есть кости становятся хрупкими и риск переломов возрастает. Если ВГГ не лечить, то остеопороз может развиться у человека в любом возрасте. Некоторым пациентам с ВГГ необходимо дополнительное лечение остеопороза.
- **снижение полового влечение и нарушение половой функции** – результат низкого уровня гормонов
- **утомляемость, сниженное настроение и депрессия** нередко наблюдаются у пациентов без лечения

У мужчин, не получающих лечения, высок риск метаболических нарушений, например преддиабета или диабета. **Однако риск можно снизить благодаря лечению.**

Ключевые моменты:

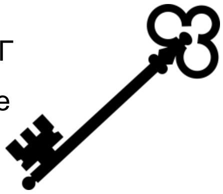

- ВГГ – это редкое заболевание, которое вызывается нехваткой ГнРГ
- При ВГГ не наступает половое созревание и отмечается бесплодие
- ВГГ не укорачивает жизнь
- Диагностировать ВГГ непросто
- в большинстве случаев ВГГ диагностируют только в позднем подростковом периоде или ранней юности
- в отличие от многих редких заболеваний в этом случае лечение возможно
- лечение необходимо получать в течение всей жизни и регулярно посещать врача
- обсудите лечение с врачом, чтобы выбрать наилучший метод
- если прекратить лечение, это может плохо отразиться на здоровье
- в большинстве случаев бесплодие можно вылечить благодаря специальному гормональному лечению
- ВГГ передаётся по наследству, поэтому необходима генетическая консультация
- у некоторых пациентов ВГГ приводит к эмоциональным проблемам
- вы можете найти поддержку в группах для пациентов и у врачей

Полезные ссылки

http://www.nature.com/nrendo/journal/v11/n9/full/nrendo.2015.112.html

Полезные сайты

http://www.gnrhnetwork.eu/hhn_home/hhn-patients_familles_gnrh_deficiency_kallmann_syndrome/hhn-onlinesupportgroups.htm

https://www.rareconnect.org/en/community/kallmann-syndrome

https://www.facebook.com/KallmannSyndrome/

(Serbian)

Šta bi trebalo da znate o kongenitalnom hipogonadotropnom hipogonadizmu (KHH) & Kalmanovom sindromu (KS)

Šta je KHH?
KHH nastaje usled **nedostatka GnRH (gonadotropin oslobađajućeg hormona)**. Ukoliko istovremeno postoji i gubitak osećaja mirisa (anosmija), radi se o olfakto-genitalnom sindromu ili Kalmanovom sindromu (KS). Ova oboljenja se na isti nacin dijagnostikuju i leče.

Kakva je uloga GnRH?
GnRH je hormon neophodan za polni razvoj, pubertet i fertilitet.

1. GnRH se oslobađa iz neurona hipotalamusa (deo mozga koji kontroliše neke od telesnih funkcija).
2. GnRH deluje direktno na hipofizu (žlezda u mozgu veličine graška, smeštena blizu hipotalamusa) da oslobodi dva hormona: luteinizirajući hormon (LH) & folikulostimulirajući hormon (FSH).
3. LH & FSH su važni za pubertet i reproduktivnu funkciju
   • kod muškaraca: LH & FSH stimulišu testise da stvaraju testosteron i spermu

   • kod žena: LH & FSH stimulišu jajnike da stvaraju estrogen & progesteron koji su važni za reprodukciju.

Šta se dešava ukoliko nema GnRH?
Potpuni ili delimični nedostatak GnRH dovodi do nedostatka ili veoma niskih koncentracija LH i FSH. Ovo za posledicu ima **izostanak puberteta i neplodnost** (vidi grafikon ispod). To znači da:

- kod muškaraca: 1) testisi ne rastu, 2) testosteron se ne stvara u dovoljnoj količini, i 3) izostaje pojava sperme.
- kod žena: 1) jajne ćelije se ne razvijaju – iako su prisutne, one ne rastu i ne oslobađaju se (ovulacija), 2) estrogen i progesteron se ne stvaraju, i 3) nema menstrualnog krvarenja .

Znaci izostanka puberteta kod adolescenata i mladih odraslih osoba

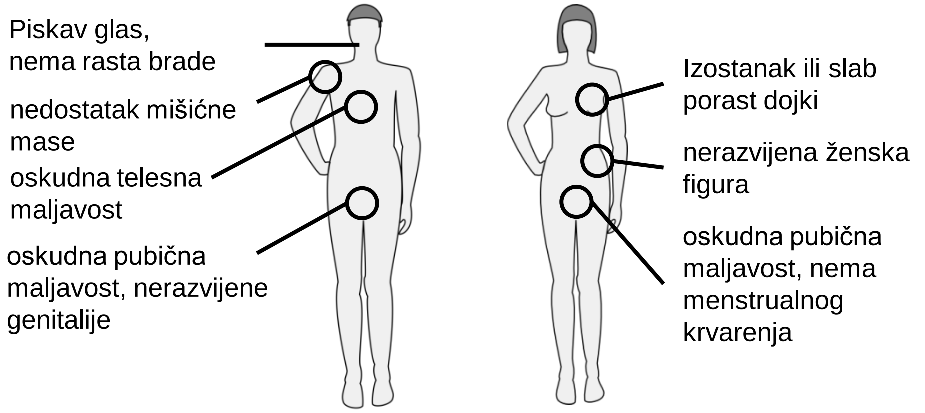

Ovi problemi su gotovo uvek prisutni već na rođenju (nazivaju se kongenitalni) ali se dijagnoza često postavlja u adolescentnom periodu ili kod mladih odraslih osoba, kada ne dolazi do pojave puberteta. **Veoma je važno istaći da se ovo stanje može lečiti. Kod najvećeg broja pacijenata, potrebna je doživotna nadoknada hormona & ostala nega.**

2017: www.gnrhnetwork.eu / andrew.dwyer@chuv.ch

Kod koga se može javiti KHH?

I muškarci i žene mogu imati ovo oboljenje. KHH se znatno češće dijagnostikuje kod muškaraca. Razlog za to nije jasan. Moguće je da mnoge obolele žene posećuju ginekologa koji im prepisuje kontraceptivne pilule kako bi regulisale mesečni ciklus, bez prethodnog postavljanja jasne dijagnoze. Kod nekih pacijenata se nikada ne postavi tačna dijagnoza i oni nikada ne budu pregledani od strane specijaliste za deficit GnRH.

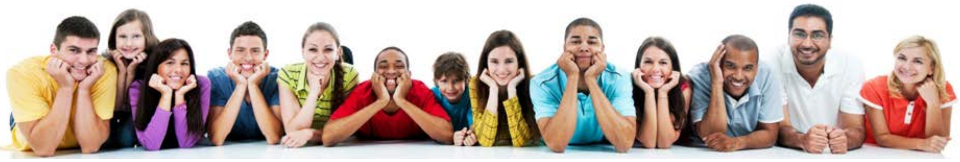

Koliko ljudi je obolelo?

KHH je retko oboljenje, tako da to nije lako tačno proceniti. Smatramo da se KHH javlja kod 1 osobe na 4.000 do 10.000 osoba. To znači da ukupan broj pacijenata sa KHH u Evropi (oko 74.000) može da ispuni Olimpijski stadion u Berlinu, Nemačka.

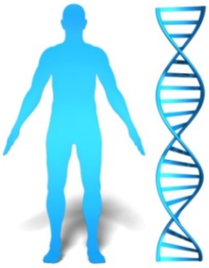

Da li se KHH nasleđuje?

KHH može da bude nasledno oboljenje. To znači da se može javiti **u nekoliko generacija** i unutar nekih porodica. U mnogim slučajevima nema jasne porodične istorije oboljenja. Genetski razlog može da se nađe kod oko polovine (50%) pacijenata. Istraživanja su u toku i u budućnosti će se otkriti genetski uzroci KHH. Lekari teško mogu tačno i precizno da predvide da li će se KHH preneti na decu pacijenata sa KHH.

Polovina pacijenata ima poznat genetski uzrok, polovina nema poznati genetski uzrok

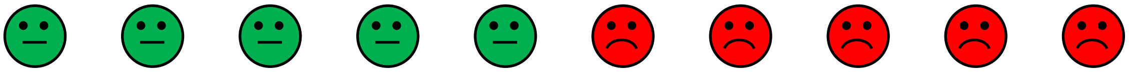

KHH je složeno oboljenje jer u nekim slučajevima KHH može biti uzrokovan kombinacijom dva ili više mutacija različitih gena. Ova činjenica **otežava predviđanje da li će se KHH javiti i kod dece pacijenata**. Za jedan gen, A*NOS1* (*KAL1*) , jednostavno je to predvideti. Dodatna ispitivanja su neophodna kako bi bolje razumeli ovo oboljenje i poboljšali njegovu dijagnostiku.

Kako se postavlja dijagnoza KHH?

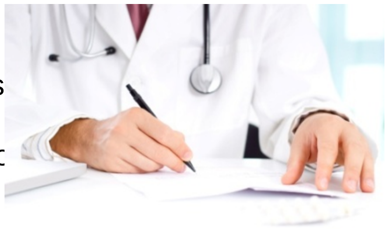

Teško je postaviti dijagnozu KHH. Veliki je opseg normalnog vremena početka puberteta. Ovo veoma otežava zapažanje da li s radi o kasnom pubertetu (ali normalnom), <u>ili</u> se radi o KHH te zahteva medicinski tretman. Redovne kontrole su neophodne kakc bi se pratio razvoj puberteta. Pacijente treba <u>uvek</u> pitati o poremećaju osećaja mirisa. Znaci koji mogu da ukažu na KHH su:

• **dečaci –** na KHH ukazuje izostanak znakova puberteta u uzrastu od 16 godina, uz nizak nivo testosterona, LH & FSH.

• **devojčice –** na KHH ukazuje izostanak razvoja grudi u uzrastu 14-15 godina, izostanak menstrualnog krvarenja u uzrastu od 16-17 godina, uz nizak nivo estrogena, LH & FSH.

• **Moraju se uraditi dodatni testovi** kako bi se isključila druga oboljenja koja mogu dovesti do niskog nivoa hormona u krvi. Rade se analize krvi i snimanja (Rentgen, ultrazvuk & NMR)

2017: www.gnrhnetwork.eu / andrew.dwyer@chuv.ch

Ima li nekih drugih ranih znakova koje treba potražiti?
Kod mnogih osoba nema drugih znakova KHH pre izostanka puberteta.

Neke bebe muškog pola mogu imati neke znakove koji ukazuju na KHH, **mikropenis ili nespuštene testise** (kriptorhidizam).

Mikropenis se može lečiti u detinjstvu hormonskom terapijom. Nespušteni testisi treba da budu operativno lečeni rano u prvoj godini života kako bi se očuvao fertilitet u budućnosti.

Postoje i drugi pridruženi znaci (ne usled KHH) koji se mogu javiti kod nekih, ali ne kod svih pacijenata.

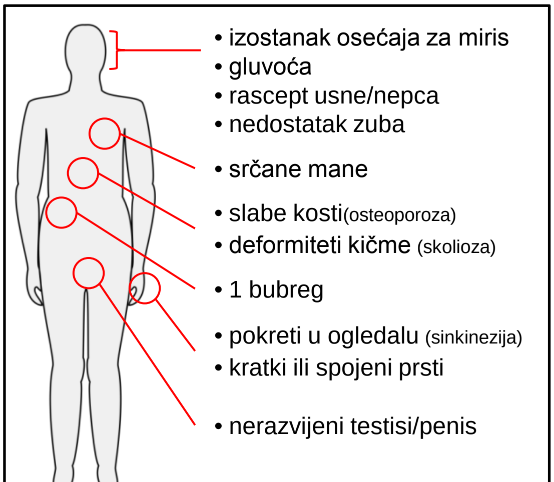

Mogući drugi znaci

Kako može izostanak puberteta uticati na život?
Izostanak puberteta u vreme kada se kod vršnjaka pubertet javlja **može biti veoma stresno i ponekada traumatično iskustvo**. Ovo može biti najznačajniji problem kod nekih pacijenata sa KHH. Posledice mogu biti u nekim slučajevima teške mada mogu varirati od osobe do osobe. Vreme adolescencije može biti teško za tinejdžere koji u normalno vreme ulaze u pubertet, ali kroz pubertet ne prolaze kao i vršnjaci, kasne za njima što može za posledicu imati:

- nisko samopoštovanje, nepoverenje
- stidljivost, teško uspostavljanje kontakta sa vršnjacima
- anksioznost, depresija
- nezadovoljstvo izgledom

Izmenjen osećaj za miris može da utiče na vaš osećaj ukusa i uživanje u hrani. Pacijenti ne mogu da osete miris gasa (ili kada se hrana pokvari). Neki pacijenti su zabrinuti zbog mirisa tela.

Šta možete uraditi?
Naposletku, ovo se sve može prevazići. Psihološko savetovanje i terapija mogu biti korisni.

1. **Pričajte sa svojim lekarom** – oni možda ne shvataju da vi imate problem. Oni vam mogu pomoći da dobijete savet i podršku.
2. **Povežite se sa drugim pacijentima –** korišćenje online grupa (Facebook, RareConnect.org) i grupe pacijenata mogu biti veoma korisne. To su mesta gde pacijenti mogu da pričaju o temama koje su im važne. Drugi pacijenti razumeju kako je živeti sa KHH svaki dan i mogu vam pružiti praktičan savet i podršku.

Šta možete uraditi kako bi bili zdravi?
KHH ne treba da skrati vaš životni vek. Postoje stvari koje možete uraditi kako bi ostali zdravi

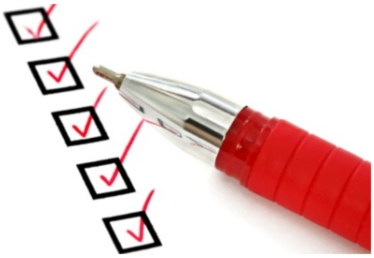

- redovno se kontrolisati kod lekara
- uzimati lekove kako je određeno
- hraniti se zdravo
- redovno se baviti fizičkom aktivnošću
- ne pušiti

2017: www.gnrhnetwork.eu / andrew.dwyer@chuv.ch

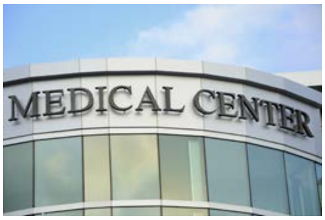

Da li je KHH izlečiva bolest?

U ovom trenutku, **ne postoji izlečenje KHH**. Veoma je teško izlečiti kongenitalni (genetski) poremećaj. Istraživanja su u toku sa ciljem da se obnovi stvaranje GnRH u hipotalamusu. Ova istraživanja su još u veoma ranoj fazi ali postoji nada da će to biti moguće u budućnosti.

Ima li lekova za KHH?

Postoje lekovi koji omogućavaju pojavu znakova puberteta (kao što je rast brade kod muškaraca i razvoj dojki kod žena). Postoji takođe posebno hormonsko lečenje za razvoj fertiliteta kod većine pacijenata.

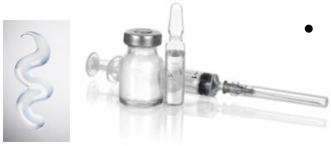

- kod muškaraca: **testosteron** (gel koji se primenjuje na kožu ili injekcije) je najčešći način lečenja kojim se razvijaju znaci puberteta. Lečenje dovodi do rasta, produbljivanja glasa, rasta brade, rasta penisa i seksualne funkcije – ali ne i fertiliteta.

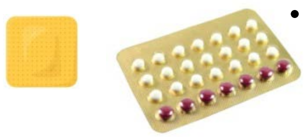

- kod žena: nisko-dozni estrogen (pilula ili flaster) dovodi do rasta i omogućava razvoj grudi , libida i ženske figure, u kombinaciji sa progesteronom dovodi do redovnih mesečnih krvarenja, ali ne i fertiliteta.

Kada se započne lečenje, **postepeno se prilagođava doza leka.** Ovo može biti frustrirajuće za neke pacijente koji možda očekuju brze rezultate. Važno je da se omogući maksimalan rast (i razvoj grudi kod žena).

Kod nekih osoba sa KHH može doći do oporavka nakon lečenja i do normalizacije nivoa hormona. Ovo je označeno kao reverzija. Razlozi za oporavak nisu jasni i ne možemo predvideti kod koga će doći do ovog oporavka. **Oporavak ne traje uvek dugo. Iz tog razloga, važno je da postoji stalni medicinski nadzor i kontrole.**

Kod oko 1 od 10 (10%) pacijenata sa KHH dolazi do oporavka

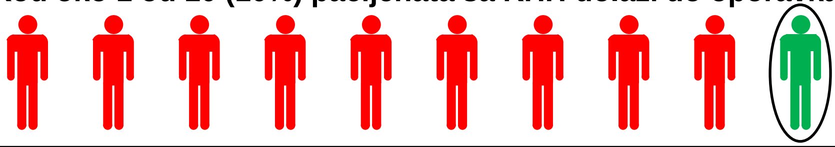

Da li osoba sa KHH može da ima potomstvo?

Da, **KHH je uzrok infertiliteta koji se može lečiti.** Najveći broj pacijenata sa KHH, ali ne svi, mogu imati potomstvo nakon **specijalizovanog hormonskog lečenja.**

Fertilitet: kod 3 od 4 pacijenta (75%) je uspešno

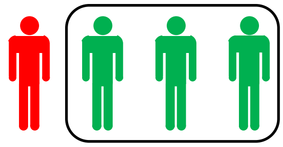

muškarci: može biti potrebno i do 2 godine za razvoj sperme

žene: u nekim slučajevima, fertilitet se uspostavlja za nekoliko meseci

- Specijalizovano lečenje obuhvata ili hormonske injekcije nekoliko puta nedeljno ili nošenje portabilne GnRH pumpe (poput one koja se koristi u dijabetesu).
- u lečenju fertiliteta neophodno je učešće iskusnih specijalista (**reproduktivni endokrinolozi**)
- ponekad (ali ne uvek) neophodno je primeniti metodu asistiranog fertiliteta, kao što je *in vitro* fertilizacija (IVF).

Ima li nekih rizika ukoliko se KHH ne leči?
Iako testosteron i estrogen nisu od vitalnog značaja, njihov nedostatak (ili deficit) može ozbiljno uticati na vaše **zdravlje, seksualnu funkciju i kvalitet života.**

<u>Kod muškaraca & žena</u>

- Povećan rizik za **manju koštanu gustinu (osteoporoza)** – ovo znači da su kosti slabe i povećan je rizik za pojavu fraktura. Ukoliko se KHH ne leči, osteoporoza može da se javi u svakom životnom dobu. Nekim pacijentima sa KHH je potrebna dodatna terapija za osteoporozu
- **Smanjena seksualna funkcija i želja** posledice su niskog nivoa hormona
- **Umor, malaksalost i depresija** su česti među pacijentima koji ne primaju terapiju

Muškarci koji se ne leče imaju povećan rizik i za pojavu metaboličkih problema, kao što su predijabetes ili dijabetes. **Ovi rizici se mogu smanjiti redovnom terapijom**.

Ključni zaključci:

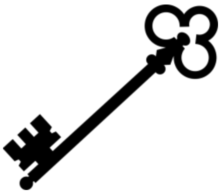

- KHH je retko oboljenje uzrokovano nedostakom GnRH
- KHH dovodi do izostanka puberteta i infertiliteta
- KHH <u>ne</u> treba da skrati vaš životni vek
- KHH se teško dijagnostikuje
- kod mnogih pacijenata dijagnoza se postavlja tek u kasnoj adolescenciji ili ranom odraslom dobu
- za razliku od mnogih retkih bolesti, ovo oboljenje se može lečiti
- potrebno vam je doživotno lečenje i redovne kontrole kod vašeg lekara
- razgovarajte sa vašim lekarom o terapijskim opcijama kako bi se donela odluka koja je za vas najbolja
- prekid terapije ima negativne posledice po vaše zdravlje i kvalitet života
- fertilitet je moguć u mnogim slučajevima uz specijalno hormonsko lečenje
- možete preneti KHH vašoj deci, tako da se preporučuje genetsko savetovanje
- KHH predstavlja psihološki problem nekim ljudima
- možete dobiti podršku od vašeg lekara i grupa pacijenata

Korisne reference
http://www.nature.com/nrendo/journal/v11/n9/full/nrendo.2015.112.html

Korisni vebsajtovi
http://www.gnrhnetwork.eu/hhn_home/hhn-patients_familles_gnrh_deficiency_kallmann_syndrome/hhn-onlinesupportgroups.htm

https://www.rareconnect.org/en/community/kallmann-syndrome

https://www.facebook.com/KallmannSyndrome/

Slovensko
(Slovenian)

Kaj je dobro vedeti o prirojenem hipogonadotropnem hipogonadizmu (PHH) & Kallmannovem sindromu (KS)

Kaj je PHH?

PHH je posledica **pomanjkanja GnRH (gonadotropin sproščujočega hormona)**. Kadar je temu pridružena še izguba občutka za vonj (anozmija), imenujemo to olfakto-genitalni sindrom ali Kallmannov sindrom (KS). Vse to na enak način diagnosticiramo in zdravimo.

Kakšna je vloga GnRH?

GnRH je hormon, nujen za spolni razvoj, puberteto in plodnost.

1. GnRH se sprošča iz hipotalamusa, področja možganov, ki nadzoruje več telesnih funkcij.
2. GnRH direktno spodbuja hipofizo (blizu v možganih ležečo žlezo velikosti graha) k sproščanju dveh hormonov: luteinizirajočega hormona (LH) & folikle stimulirajočega hormona (FSH).
3. LH & FSH sta bistvena za puberteto in omogočata plodnost
   - Pri moških: LH & FSH spodbujata sproščanje testosterona in tvorbo semena v modih
   - Pri ženskah: LH & FSH spodbujata jajčnike k sproščanju estrogena in progesterona, ki sta pomembna za plodnost.

Kaj se zgodi, če se GnRH ne izloča?

Popolno ali delno pomanjkanje GnRH povzroči odsotna ali znižana LH in FSH. To povzroči **odpoved pubertetnega razvoja in neplodnost** (glej sliko spodaj). To pomeni, da:

- pri moških: 1) moda ne rastejo, 2) testosteron se ne sprošča v normalnih količinah, in 3) seme se ne tvori.
- Pri ženskah: 1) čeprav so jajčeca prisotna, jajčeca ne zorijo ali rastejo in se ne sproščajo (ovulacija), 2) ne sproščata se estrogen in progesteron, in 3) ni menstruacije (krvavitve).

Znaki odpovedi pubertetnega razvoja pri mladostnikih in mladih odraslih

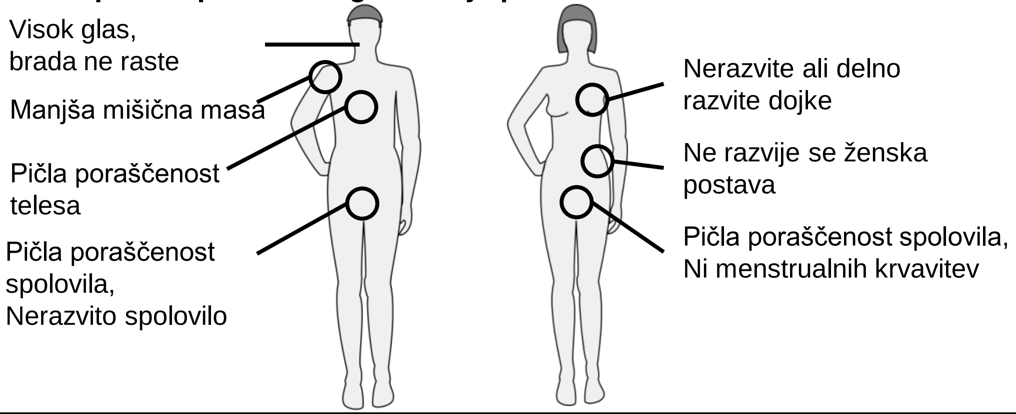

Motnja je skoraj praviloma prisotna že ob rojstvu (zato se imenuje prirojena), toda ugotovimo jo običajno v adolescenci ali zgodnji odrasli dobi, ko ni začetka pubertete. **Pomembno je, da to motnjo lahko zdravimo. V večini primerov zahteva doživljenjsko nadomeščanje hormonov & zdravniško spremljanje.**

2017: www.gnrhnetwork.eu / andrew.dwyer@chuv.ch

Kdo zboli za PHH?

Moški in ženske lahko zbolijo. PHH je pogosteje ugotovljen pri moških. Razlog za to ni znan. Možno je, da številne prizadete ženske obiščejo ginekologa, pri katerem dobijo recept za kontracepcijske tablete, s katerimi imajo reden mesečen ciklus, vendar ne izvejo nujno jasne diagnoze. Nekateri bolniki nikoli ne dobijo točne diagnoze in lahko nikoli ne obiščejo specialista za pomanjkanje GnRH.

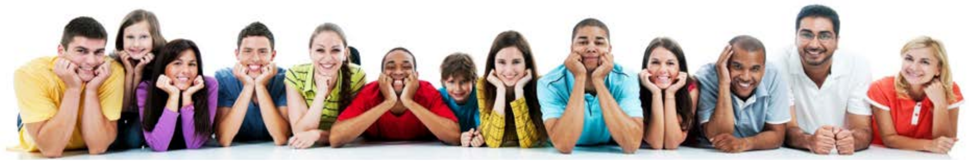

Koliko ljudi ima to bolezen?

PHH je redka bolezen zato točna ocena ni možna. Mislimo, da za PHH zboli približno 1 na 4.000 do 10.000 ljudi. To pomeni, da bi celotno število bolnikov s PHH v Evropi (okrog 74.000) napolnilo Olimpijski stadion v Berlinu v Nemčiji.

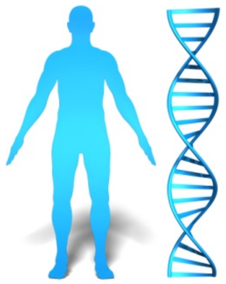

Je PHH dedna bolezen?

PHH je lahko dedna bolezen. To pomeni, da **se lahko deduje** skozi več generacij in znotraj družin. V mnogih primerih ni znanih dodatnih obolelih v družini. Genetski vzrok lahko ugotovimo pri približno polovici (50%) bolnikov. Raziskave tečejo in verjetno bo sčasoma več znanega o genetskih vzrokih PHH. V večini primerov zdravniki težko zanesljivo in točno napovejo, ali bodo otroci bolnika s PHH podedovali bolezen.

Pri polovici bolnikov lahko ugotovimo genetski vzrok, pri polovici pa ne.

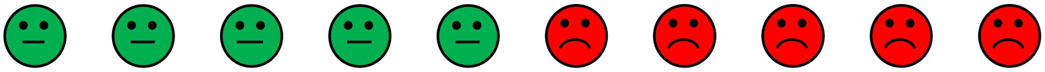

PHH je zapletena bolezen, namreč v nekaterih primerih bolezen povzroči kombinacija dveh ali več sprememb (mutacij) v različnih genih. Zato **je izziv napovedati ali bodo otroci bolnika s PHH podedovali bolezen ali ne.** Za en gen, ANOS1 (KAL1), je lahko napovedati. Več podatkov iz raziskav je potrebnih za boljše razumevanje tega in natančnejšo diagnozo.

Kako postavimo diagnozo PHH?

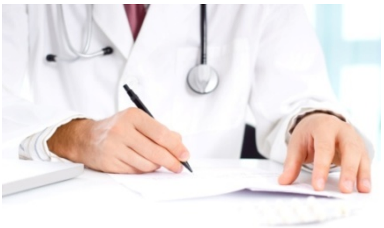

Težko je postaviti diagnozo PHH. Obstaja širok starostni razpon normalnega pričetka pubertetnega razvoja. Zato se je težko odločiti, ali gre za kasno puberteto (toda normalno), ali gre za PHH in je potrebno zdravljenje. Za spremljanje napredovanja pubertete so potrebni redni zdravniški pregledi. Bolnike je treba vedno vprašati o morebitni motnji občutka za vonj. Znaki, ki nakazujejo, da gre lahko za PHH:

- **dečki –** ni pubertete do 16. leta starosti z nizkimi vrednostmi testosterona, LH & FSH.
- **dekleta –** ni razvoja dojk do 14-15. leta starosti in ni menstruacij do 16-17. leta starosti z nizkimi vrednostmi estrogena, LH & FSH.
- **Opraviti je potrebno dodatne preiskave** s katerimi opredelimo morebitne druge vzroke za nizke vrednosti hormonov. Preiskave vključujejo krvne preiskave in slikovne preiskave (rentgen, ultrazvok & MRI)

Kako lahko PHH vpliva na moje zdravje in življenje?

Kateri so zgodnji znaki bolezni?

Številni pred odpovedjo pubertetnega razvoja nimajo nobenih znakov PHH.

Nekateri fantki imajo kot dojenčki **manjše spolovilo** ali **nespuščena moda** (kriptorhidizem), kar so znaki, ki lahko nakazujejo PHH.

Manjše spolovilo lahko zdravimo s hormonskim zdravljenjem v obdobju dojenčka ali v otroštvu. Nespuščena moda je zaradi vpliva na kasnejšo plodnost treba operativno oskrbeti zgodaj, v prvem letu življenja.

Možni so drugi znaki, nepovezani s PHH, ki so pri nekaterih, toda ne vseh posameznikih.

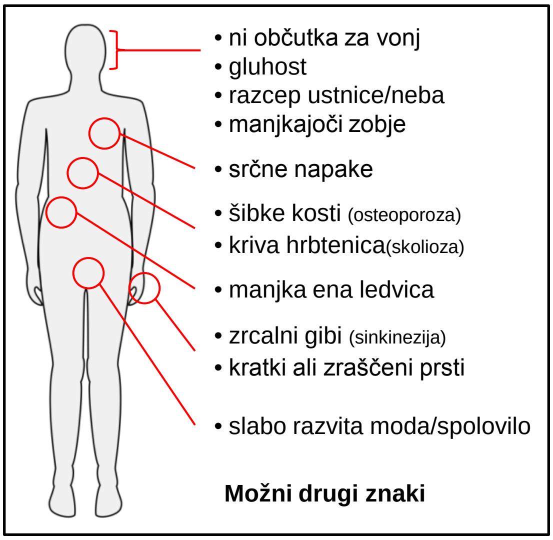

Kako lahko na posameznika vpliva odsotnost pubertete?

Kasnitev vstopa v puberteto glede na vrstnike je lahko **zelo stresno in včasih travmatično**. Nekaterim bolnikom s PHH lahko predstavlja to eno od glavnih težav. Na nekatere je lahko vpliv tega zelo močan, vendar je različen od osebe do osebe. Najstniška leta so lahko naporna za najstnike, ki vstopijo v puberteto v običajni starosti, toda ne vstopiti v puberteto in izostati za vrstniki lahko povzroči naslednje težave:

- nizko samopodobo, malo zaupanja vase
- sramežljivost, težave v stikih z vrstniki
- strah, depresijo
- slabo telesno podobo

Motnja v občutku za vonj lahko vpliva na občutek za okus in uživanje hrane. Ljudje so lahko nezmožni zaznati plin (ali pokvarjena živila). Nekateri so lahko zaskrbljeni zaradi telesnega vonja.

Kaj lahko storite?

Težave so rešljive. V pomoč je lahko psihološko svetovanje in zdravljenje.

1. **Pogovorite se s svojim zdravnikom**- morda se ne zaveda, da vam je težko. Lahko vam pomaga najti nasvet in pomoč.
2. **Povežite se z drugimi bolniki –** v pomoč so lahko skupine na medmrežju (Facebook, RareConnect.org) ali srečanja podpornih skupin. To so mesta, kjer lahko bolniki razpravljajo o zanje pomembnih težavah. Drugi bolniki razumejo, kako je živeti s PHH iz dneva v dan, in lahko nudijo praktične nasvete in podporo.

Kaj morate storiti, da boste zdravi?

Zaradi PHH naj bi vaše življenje ne bilo krajše. Da ostanete zdravi, je potrebno, da:

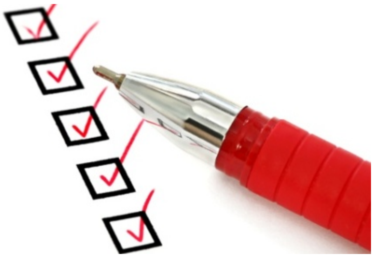

- imate redno zdravniško oskrbo in preglede
- jemljete predpisana zdravila
- se zdravo prehranjujete
- vzdržujete redno telesno aktivnost
- ne kadite cigaret

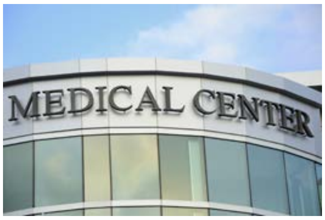

Je PHH ozdravljiva bolezen?

Trenutno **ni zdravila, ki bi lahko odpravilo PHH**. Zelo težko je pozdraviti prirojeno (genetsko) bolezen. V teku so raziskave s katerimi se skuša vzpostaviti tvorbo GnRH v hipotalamusu. Te raziskave so za zdaj še v zelo zgodnji fazi, toda želeli bi si, da bi bilo v prihodnosti to mogoče.

Ali je na voljo zdravljenje PHH?

Obstaja zdravljenje, ki omogoča razvoj pubertetnih znakov, kot sta rast brade pri moških in razvoj prsi pri ženskah. Na voljo so tudi posebna hormonska zdravljenja, ki večini posameznikov omogočijo plodnost.

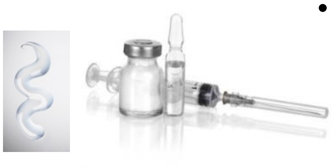

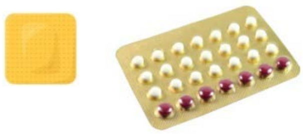

- Moški: **testosteron** (gel, ki se ga nanese na kožo ali injekcije) je najpogosteje uporabljeno zdravljenje za razvoj pubertetnih znakov. Zdravljenje spodbudi rast, znižanje glasu, rast brade, rast spolovila, spolno slo (libido) in spolno funkcijo – toda ne plodnosti.
- Ženske: nizek odmerek **estrogena** (tablete ali obliži) spodbudi rast in omogoči razvoj prsi in ženske postave ter spolno slo (libido), v kombinaciji s progesteronom pa omogoči reden menstrualni cikel (krvavitve) – toda ne plodnosti.

Ob začetku zdravljenja je potrebno **odmerek postopno prilagajati**. To je lahko za koga, ki si želi hitrih rezultatov, moteče, vendar je pomembno za optimalno rast (in razvoj prsi pri ženskah)

Nekateri ljudje s PHH ozdravijo po zdravljenju in lahko izločajo normalne vrednosti hormonov. To imenujemo preobrat. Razlogov za ta pojav ne razumemo in tudi ne znamo napovedati, pri komu bo prišlo do takega preobrata. **Tudi to ozdravljenje ni vedno trajno. Zato je pomembno, da zdravstveno stanje bolnika spremlja zdravnik.**

Približno 1 od 10 (10%) bolnikov s PHH doživi preobrat

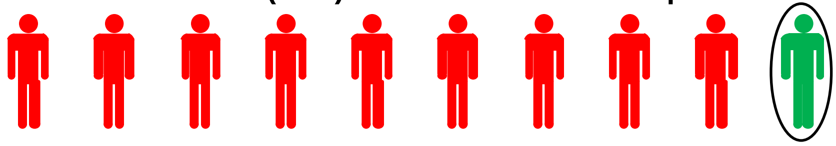

Ali oseba s PHH lahko postane plodna?

Da, **PHH je ozdravljiva oblika neplodnosti.** Večinoma - toda ne vsi - ljudje s PHH lahko postanejo plodni s pomočjo **specialnega hormonskega zdravljenja.**

Plodnost: 3 od 4 bolnikov (75%) so uspešni

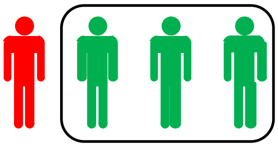

moški: lahko traja tudi do 2 leti, da se razvije seme
ženske: v nekaterih primerih se plodnost doseže v nekaj mesecih

- Specialno zdravljenje so lahko ali hormonske injekcije večkrat tedensko ali nošenje prenosne GnRH črpalke (podobne inzulinski črpalki, ki se uporablja za sladkorno bolezen).
- zdravljenje plodnosti zahteva obravnavo izkušenih specialistov (**reproduktivnih endokrinologov**)
- včasih (toda ne vedno) je potrebna oploditev z biomedicinsko pomočjo, kot je zunajtelesna oploditev (in vitro fertilizacija (IVF)).

Kakšna so tveganja, če PHH ne zdravimo?
Čeprav testosteron in estrogen nista ključna za preživetje, lahko njuna odsotnost (ali pomanjkanje) resno vpliva na **zdravje, spolno funkcijo in kvaliteto življenja**.

Pri moških & ženskah
•Višje tveganje za **znižano kostno gostoto (osteoporozo)** – to pomeni, da so kosti šibke in obstaja večje tveganje za zlome. Če PHH ne zdravimo, lahko osteoporoza prizadene človeka v katerikoli starosti. Nekateri posamezniki s PHH potrebujejo dodatno zdravljenje za osteoporozo.
•**Manjša spolna funkcija in želja** sta posledici nizke ravni hormonov.
•**Utrujenost, potrtost in depresija** so pogosti pri osebah brez zdravljenja.

Moški brez zdravljenja imajo tudi večje tveganje za presnovne zaplete, kot je sladkorna bolezen. **Ta tveganja lahko zmanjšamo z rednim zdravljenjem**.

Ključni poudarki:

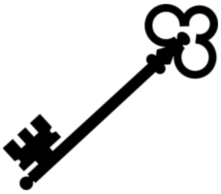

- PHH je redka bolezen, katero povzroča pomanjkanje GnRH
- Posledici PHH sta odsotnost pubertete in neplodnost
- PHH naj ne bi skrajšal vašega življenja
- Diagnozo PHH težko postavimo
- Pri mnogih se diagnoza ugotovi šele v pozni adolescenci ali zgodnji odrasli dobi
- Za razliko od mnogih redkih bolezni, zdravljenje obstaja
- Potrebujete doživljenjsko zdravljenje in redno zdravniško obravnavo
- Pogovorite se z zdravnikom in se odločite, katero zdravljenje je za vas najboljše
- Prenehanje zdravljenja ima negativne posledice za vaše zdravje in počutje
- Plodnost je večinoma možna s pomočjo specifičnega hormonskega zdravljenja
- PHH se lahko prenese na vaše otroke, zato je priporočeno genetsko svetovanje
- PHH lahko za nekatere predstavlja psihološko breme
- Podporo lahko najdete pri zdravstvenem osebju in skupinah bolnikov

Priporočena literatura
http://www.nature.com/nrendo/journal/v11/n9/full/nrendo.2015.112.html

Priporočene medmrežne strani
http://www.gnrhnetwork.eu/hhn_home/hhn-patients_familles_gnrh_deficiency_kallmann_syndrome/hhn-onlinesupportgroups.htm

https://www.rareconnect.org/en/community/kallmann-syndrome

https://www.facebook.com/KallmannSyndrome/

(Español)

Qué debes saber sobre el hipogonadismo hipogonadotropo congénito (HHC) y síndrome de Kallmann (SK)

¿Qué es el HHC?

El HHC está causado por una **deficiencia de GnRH (hormona liberadora de gonadotropina).** Cuando se combina con una ausencia del sentido del olfato (anosmia), se le conoce como síndrome olfato-genital o síndrome de Kallmann (SK). Todos ellos son diagnosticados y tratados de la misma forma.

¿Qué hace la GnRH?

La GnRH es una hormona esencial para el desarrollo sexual, pubertad y fertilidad.

1. La GnRH es liberada desde el hipotálamo, un área del cerebro que controla diversas funciones corporales.
2. La GnRH actúa directamente sobre la glándula hipofisaria (una glándula cercana en el cerebro, del tamaño de un guisante) para que libere dos hormonas: la hormona luteinizante (LH) y la folículo estimulante (FSH).
3. La LH y la FSH son importantes para la pubertad y fertilidad
   - en hombres: LH y FSH estimulan los testículos para fabricar testosterona y esperma
   - en mujeres: LH y FSH estimulan los ovarios para fabricar estrógenos y progesterona que son importantes para la fertilidad.

¿Qué ocurre si no hay GnRH?

La deficiencia completa o parcial de GnRH produce ausencia o niveles muy bajos de LH y FSH. Esto ocasiona un **fallo en la pubertad e infertilidad** (ver figura debajo). Esto significa que:

- en hombres: 1) los testículos no crecen 2) no se produce testosterona en cantidades normales y 3) el esperma no se desarrolla.
- en mujeres: 1) aunque los óvulos están presentes no se desarrollan o crecen y no son liberados (ovulación) 2) los estrógenos y la progesterona no son producidos, y 3) no aparecen periodos menstruales (sangrado).

Signos de pubertad fallida en adolescentes y adultos jóvenes

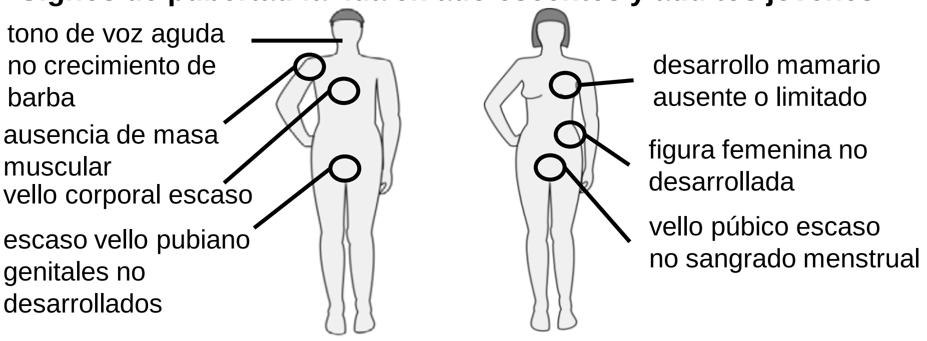

Estos problemas están casi siempre presentes al nacimiento (llamado congénito). Sin embargo, el diagnóstico se hace con frecuencia durante la adolescencia o principio de la edad adulta cuando no se inicia la pubertad. Notablemente**, esta enfermedad es tratable. En la mayoría de los casos requiere sustitución hormonal de por vida & atención continuada.**

2017: www.gnrhnetwork.eu / andrew.dwyer@chuv.ch

¿A quién afecta el HHC y cómo se diagnostica?

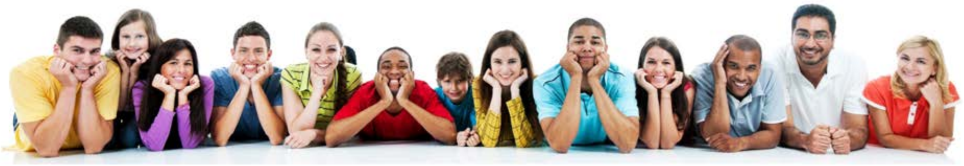

¿A quién afecta el HHC?

Afecta tanto a hombres como a mujeres. El HHC es más frecuentemente diagnosticado en hombres. La razón de esto no está clara. Puede ser porque muchas mujeres afectas van al ginecólogo y se les receta la « píldora » anticonceptiva para tener menstruaciones regulares, y pueden no obtener un diagnóstico claro. Algunos pacientes no logran nunca un diagnóstico exacto y pueden no ver nunca un especialista para la deficiencia de GnRH.

¿Cuántas personas hay afectadas?

El HHC es raro, por lo que es difícil realizar un cálculo preciso. Creemos que el HHC occurre en alrededor de 1 de cada 4.000 a 10.000 personas. Esto significa que el número total de pacientes con HHC en Europa (alrededor de 74.000) llenaría el estadio Olímpico de Berlín, Alemania.

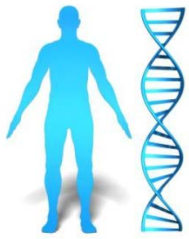

¿El HHC es heredado?

El HHC puede ser hereditario. Esto significa que **puede ser transmitido** a través de las generaciones y dentro de las familias. En muchos casos, no hay historia familiar aparente. Una causa genética puede ser identificada en alrededor de la mitad (50%) de las ocasiones. Se sigue investigando y queda más por descubrir sobre las causas genéticas del HHC. En la mayoría de los casos es difícil para los profesionales sanitarios predecir de forma fiable y precisa si un paciente transmitirá el HHC a sus hijos.

La mitad de los pacientes tienen una causa genética conocida, y la otra mitad no

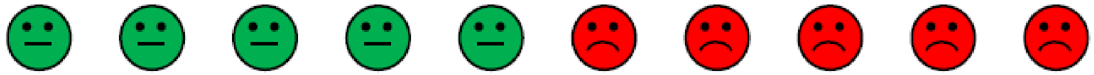

El HHC es complicado porque en algunos casos puede ser causado por una combinación de dos o más cambios (mutaciones) en genes diferentes. Esto hace **complicado predecir si el HHC será transmitido a los hijos.** Para un gen, *ANOS1* (*KAL1*), es sencillo de predecir. Se necesita más investigación para entender mejor esto y mejorar el diagnóstico.

¿Cómo se diagnostica el HHC?

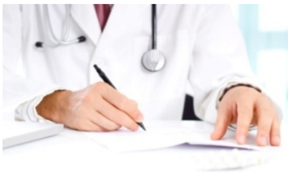

El HHC es difícil de diagnosticar. Hay un amplio margen en el momento normal de la pubertad. Esto hace muy difícil decidir si la pubertad es retrasada (pero normal) o si es un HHC y requiere tratamiento médico. Se necesitan citas regulares para seguir la progresión de la pubertad. Los pacientes siempre deben ser preguntados sobre un sentido del olfato defectuoso. Son signos que apuntan a HHC:

• **chicos -** no signos de pubertad a los 16 años con baja testosterona, LH & FSH.

• **chicas -** no desarrollo mamario a los 14-15 años y ausencia de periodo menstrual (sangrado) a los 16-17 años con bajos estrógenos, LH y FSH.

• **Se deben hacer otros tests** para asegurar que no haya otros problemas causando los bajos niveles hormonales. Los tests incluyen análisis de sangre y estudios de imagen (radiografías, ecografía & RM)

¿Existen signos tempranos para buscar?

Para muchas personas no hay signos de HHC antes del fallo en la pubertad.

Algunos niños varones pueden mostrar signos sugestivos de HHC, **micropene o testículos no descendidos** (criptorquidismo).

El micropene puede ser tratado en la infancia / niñez con tratamiento hormonal.
Los testículos no descendidos deberían ser corregidos mediante cirugía de forma precoz en los primeros años de vida para ayudar a la fertilidad futura.

Hay signos adicionales, no debidos al HHC, que aparecen en algunos pacientes pero no en todos.

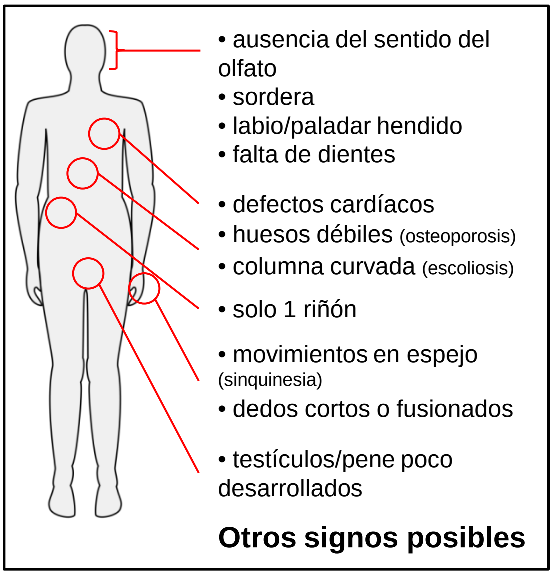

¿Cómo afectaría a alguien la ausencia de pubertad?

No tener la pubertad al mismo tiempo que los compañeros **puede ser muy estresante y algunas veces traumático**. Este puede ser un problema importante para algunos pacientes con HHC. El impacto puede ser severo en algunos casos aunque variará de una persona a otra. Los años de la adolescencia pueden ser difíciles para los adolescentes que inician la pubertad en el momento normal, pero no atravesar la pubertad y ser dejado atrás por el grupo de compañeros puede derivar en potenciales cuestiones como:

- baja autoestima, poca confianza
- timidez, dificultad para interactuar con compañeros
- ansiedad, depresión
- pobre imagen corporal

Un sentido anormal del olfato puede afectar tu sentido del gusto y disfrute de la comida. Las personas pueden no ser capaces de detectar gas (o cuando los alimentos se deterioran). Algunos pueden estar preocupados por el olor corporal.

¿Qué puedes hacer?

Por lo general, esto puede ser superado. El asesoramiento y terapia psicológicos pueden ser útiles.

1. **habla con tu profesional sanitario -** ellos pueden no darse cuenta de que estás pasando por un momento difícil. Ellos te pueden ayudar a encontrar asesoramiento y apoyo.
2. **conecta con otros pacientes -** usar grupos online (Facebook, RareConnect.org) y grupos de apoyo presencial puede ser muy útil. Son sitios donde los pacientes pueden hablar de los asuntos que son importantes para ellos. Los otros pacientes entienden cómo es vivir el día a día con HHC y pueden proporcionar consejo práctico y apoyo.

¿Qué deberías hacer para estar sano?

Tener HHC no debería acortar tu vida. Hay cosas que puedes hacer para permanecer sano

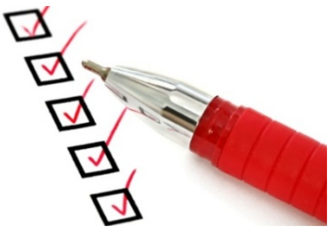

- tener atención sanitaria continua y consultas
- tomar tu tratamiento como está prescrito
- comer una dieta sana
- hacer ejercicio regularmente
- no fumar tabaco

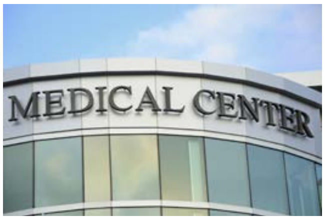

¿El HHC es curable?
Actualmente **no existe cura para el HHC**. Es muy difícil curar un trastorno congénito (genético). Hay investigaciones en marcha para ver si podemos restaurar la producción de GnRH por el hipotálamo. Esta investigación está todavía en un estadío muy temprana pero se espera que esto sea posible en el futuro.

¿Hay tratamientos disponibles para el HHC?
Hay tratamientos disponibles para desarrollar los signos externos de la pubertad -como crecimiento de la barba en los hombres o desarrollo mamario en las mujeres-. También hay tratamientos hormonales especiales para ayudar a desarrollar la fertilidad en la mayoría de los casos.

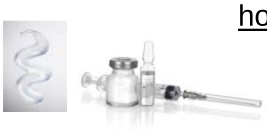

hombres: **Testosterona** (gel de aplicación cutánea o inyecciones) es el tratamiento más común para desarrollar los signos de la pubertad. El tratamiento induce el crecimiento, engrosamiento de la voz, crecimiento del pene, e impulso sexual (libido) - pero no la fertilidad.

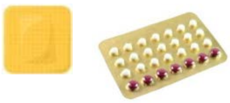

mujeres: Estrógenos a dosis bajas (parche o píldoras) inducen el crecimiento y ayudan a desarrollar las mamas, el impuso sexual (libido) y la figura femenina, combinados con progesterona provocan periodos menstruales regulares (sangrados) - pero no la fertilidad.

Cuando se inicia el tratamiento por primera vez, **el ajuste de dosis es gradual**. Esto puede ser frustrante para algunas personas que podrían esperar resultados rápidos. Sin embargo, es importante para maximizar el crecimiento (y el desarrollo mamario en mujeres).

Unas pocas personas con HHC se recuperan tras el tratamiento y pueden producir niveles hormonales normales. A esto se le llama « una reversión ». Las razones de esto no son comprendidas y no somos capaces de predecir quién va a tener esta reversión. **Esta recuperación no siempre perdura. Por eso es importante ser seguido de cerca por un profesional sanitario.**

Alrededor de 1 de cada 10 (10%) pacientes con HCC tiene una reversión

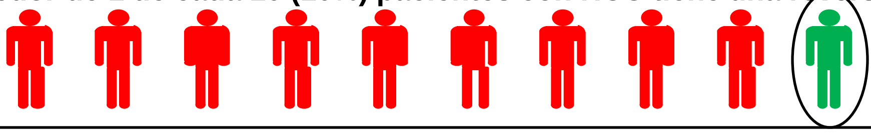

¿Puede una persona con HCC llegar a ser fértil?
Sí, el **HHC es una forma tratable de infertilidad.** La mayoría de las personas con HHC - pero no todas - pueden llegar a ser fértiles con **tratamiento hormonal especializado.**

- el tratamiento especializado puede ser o bien inyecciones varios días a la semana o llevar una bomba portátil dispensadora de GnRH (como la usada para la diabetes)
- el tratamiento para la fertilidad requiere atención por especialistas experimentados **(endocrinólogos reproductivos)**
- a veces (pero no siempre) se necesita reproducción asistida, como fecundación *in vitro* (FIV).

Fertilidad: Globalmente, 3 de cada 4 pacientes (75%) tienen éxito

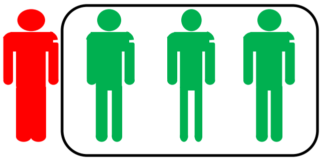

hombres: se puede tardar hasta 2 años en desarrollar esperma

mujeres: en algunos casos se consigue la fertilidad en unos pocos meses

¿Existe algún riesgo si el HHC no es tratado?
Aunque la testosterona y los estrógenos no son hormonas esenciales para la vida, su ausencia (o déficit) puede afectar seriamente la **salud, función sexual y calidad de vida.**

En hombres & mujeres
- riesgo aumentado de **baja densidad ósea (osteoporosis)** – esto significa que los huesos son débiles y hay un riesgo de fracturas mayor de lo normal. Si el HHC no es tratado, la osteoporosis puede afectar a una persona a cualquier edad. Algunas personas con HHC requieren tratamiento extra para la osteoporosis.
- **la disminución de la función y deseo sexual** es el resultado de los bajos niveles hormonales
- **la fatiga, ánimo decaído y depresión** son comunes en pacientes sin tratamiento

Los hombres sin tratamiento tienen también más riesgo de tener problemas metabólicos como pre-diabetes o diabetes. **Estos riesgos pueden ser reducidos permaneciendo en tratamiento.**

Puntos clave:

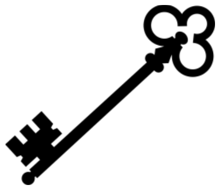

- el HHC es una enfermedad rara causada por deficiencia de GnRH
- el HHC resulta en ausencia de pubertad e infertilidad
- el HHC no debería acortar tu vida
- el HHC es difícil de diagnosticar
- muchas personas no son diagnosticadas hasta el final de la adolescencia o inicio de la edad adulta
- a diferencia de muchas enfermedades raras, existen tratamientos disponibles
- necesitas tratamiento de por vida y consultas regulares con tu médico
- habla con tu médico sobre opciones de tratamiento para decidir qué es mejor para ti
- suspender el tratamiento tiene consecuencias negativas sobre tu salud y bienestar
- la fertilidad es posible en la mayoría de los casos con tratamiento hormonal especial
- puedes transmitir el HHC a tus hijos, por lo que se recomienda consejo genético
- el HHC es psicológicamente difícil para algunas personas
- puedes encontrar apoyo en tus profesionales sanitarios y grupos de pacientes

Referencia útil
http://www.nature.com/nrendo/journal/v11/n9/full/nrendo.2015.112.html

Páginas web útiles
http://www.gnrhnetwork.eu/hhn_home/hhn-patients_familles_gnrh_deficiency_kallmann_syndrome/hhn-onlinesupportgroups.htm

https://www.rareconnect.org/en/community/kallmann-syndrome

https://www.facebook.com/KallmannSyndrome/

Konjenital hipogonadotropik hipogonadizm (KHH) ve Kallmann Sendromu (KS) hakkında bilmeniz gerekenler.

KHH nedir?

Konjenital hipogonadotropik hipogonadizm, Gonadotropin salma hormonu eksikliği nedeniyle oluşur. Koku duygusunun eksikliği (anosmi) ile birleştiğinde Olfacto-genital sendrom veya Kallmann sendromu olarak bilinir. Tüm bunlar aynı şekilde teşhis edilir ve tedavi edilir.

Gonadotropin salgılayan hormon ne yapar?

Gonadotropin, cinsel gelişim, ergenlik ve doğurganlık için gerekli bir hormondur.

1. Gonadotropin salgılayan hormon, beyindeki çeşitli vücut işlevlerini kontrol eden bir bölgedeki hipotalamustan salınır.
2. Gonadotropin salgılayan hormon iki hormonu serbest bırakmak için doğrudan hipofiz bezi üzerinde hareket eder: lüteinize edici hormon (LH) ve folikül stimüle edici hormon (FSH).
3. Ergenlik ve doğurganlık için lüteinize edici hormone (LH) ve folikül stimüle edici hormone (FSH) önemlidir.

- Erkeklerde: lüteinize edici hormon (LH) ve, folikül stimüle edici hormon (FSH) testisleri testosteron ve sperm oluşturmak için uyarır.

- Kadınlarda: LH ve FSH yumurtalığı uyarır ve doğurganlık için önemli olan östrojen ve progesteron yaratır

Gonadotropin salgılayan hormon (GnRH) yoksa ne olur? Komple veya kısmi Gonadotropin salgılayan hormon (GnRH) eksikliği, LH ve FSH'nin mevcut olmamasına veya çok düşük olmasına neden olur. Bu ergenlik ve infertilitede başarısızlıkla sonuçlanır (aşağıdaki şekle bakınız). Bunun anlamı şudur:

- **erkeklerde**: 1) testisler büyümez, 2) testosteron normal miktarda üretilmez ve 3) sperm gelişmez.

- **kadınlarda**: 1) yumurta mevcut olmasına rağmen, gelişme ya da çoğalma ve salınmazlar (ovülasyon), 2) östrojen ve progesterone üretilmez ve 3) menstrual dönem (kanama) oluşmaz.

Ergenlerde ve genc eriskinlrde basarisiz ergenlik belirtileri

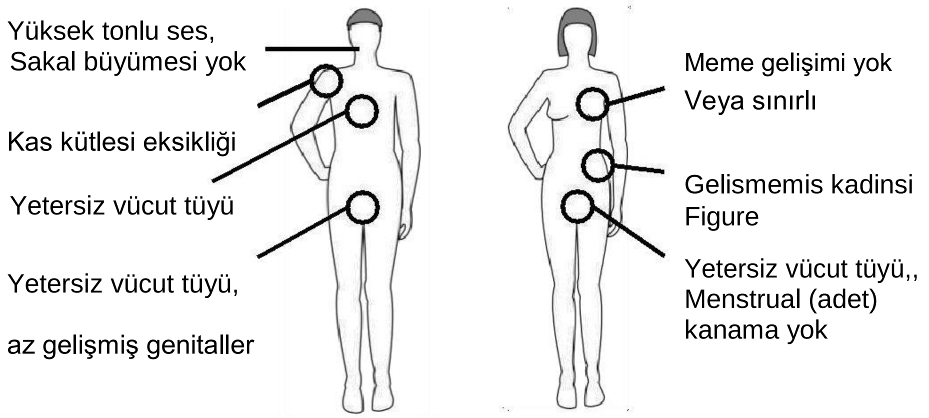

Bu problemler doğumda neredeyse her zaman mevcuttur (doğuştan). Bununla birlikte, tanı genellikle ergenlik başlamadığı gençlik yıllarında veya erken yetişkinlikte yapılır. **Önemlisi, bu durum tedavi edilebilir. Çoğu durumda yaşam boyu hormon replasmanı ve süren bakım gerektirir.**

Kim KHH'den etkileniyor ve nasıl teşhis konuyor?

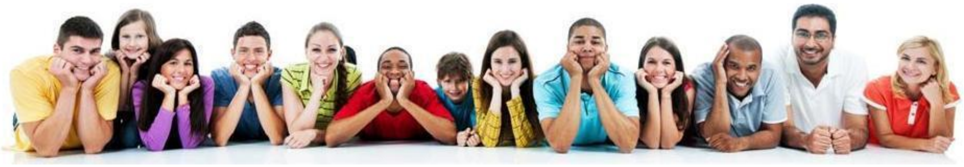

KHH'den kimler etkileniyor?

Hem erkekler hem de kadınlar etkileniyor. Erkeklerde KHH sıklıkla teşhis edilir. Bunun nedeni açık değildir. Bir sürü kadının jinekolog görmeye gidebilir ve kontraseptif "hap" reçetesi verilebilir duzenli adet için. Ama bir net teşhis alamayabilirler. Bazı hastalar teşhis almaz ve GnRH eksikliği için bir uzman görmez.

Kaç kişiyi etkiliyor?

KHH nadirdir, bu nedenle doğru bir tahminde bulunmak zordur. KHH, yaklaşık 1'de 4000 ila 10.000 kişide görülüyor. Bu, Avrupa'da KHH hastalarının (yaklaşık 74,000) tümünün Almanya'nın Berlin'deki Olimpiyat Stadyumunu dolduracağı anlamına geliyor.

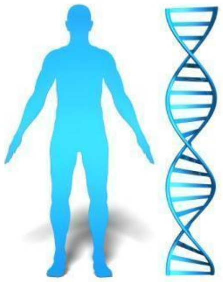

KHH kalıtsal mı? KHH kalıtsal olabilir. Bu, **nesiller boyunca ve aileler arasında geçirilebilir anlamına gelir.** Birçok durumda, belirli bir aile geçmişi yoktur. Genetik bir neden, çok zaman yüzde elliye kadar (% 50) tespit edilebilir. Araştırma devam etmekte olup KHH'nin genetik nedenleri hakkında daha fazla şey keşfedilmelidir. Çoğu durumda, sağlık hizmeti sunucuları bir hastanın KHH'yi çocuklarına geçirimesi güvenilir ve doğru bir şekilde tahmin etmek zordur.

Hastaların yarısı bilinen bir genetik nedene, yarısının genetik nedeni bilinmemektedir.

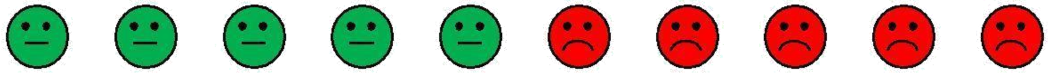

KHH karmaşıktır, çünkü bazı durumlarda KHH, farklı genlerdeki iki veya daha fazla değişiklik (mutasyon) kombinasyonundan kaynaklanabilir. Bu **KHH'nin çocuklara geçip geçmeyeceğini tahmin etmeyi zorlaştırıyor**. Bir gen ANOS1 (KAL1) için, öngörmek basittir. Bunu daha iyi anlamak ve teşhisi geliştirmek için daha fazla araştırmaya ihtiyaç vardır

KHH nasıl teşhis edilir?

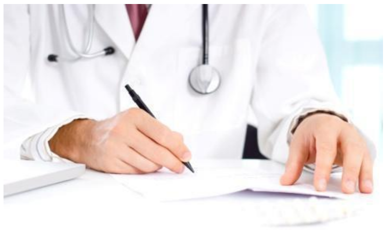

KHH teşhisi zor. Ergenlik normal zamanlamasında geniş bir aralık vardır. Bu ergenlik ertelenip, (ancak normaldir) karar vermeyi zorlaştırır, veya nedeni KHH olup, tıbbi tedaviye gerek duyulursa. Ergenlik gelişimini izlemek için düzenli randevulara ihtiyaç vardır. Hastalara daima kusurlu koku duyusu sorulmalıdır. KHH'ye işaret eden işaretler şunlardır:

erkek çocuklar - düşük testosteron, LH ve FSH ile 16 yaşına kadar ergenlik belirtisi yok.

kızlar - 14-15 yaşlarına kadar göğüs gelişiminde yok, azalma ve düşük östrojen, LH ve FSH ile 16-17 yaşlarına kadar menstrual dönem (kanama) yok

Düşük hormon düzeyine neden olan başka bir sorun olmadığından emin olmak için diğer testler yapılmalıdır. Testler arasında kan testleri ve görüntüleme çalışmaları (röntgen, ultrason ve MR

Aranacak ilk işaretler var mı?

Birçok kişi için ergenlik başarısızlığından Önce KHH belirtisi görülmez.

Bazı erkek bebekler KHH,mikropenis veya inmemiş testisleri (kriptorşidizm) işaret eden işaretler gösterebilir.

Mikropenis bebeklik döneminde / çocukluk çağında hormon tedavisi ile tedavi edilebilir.

Gelecekte doğurganlığa yardımcı olmak için, inmemiş testisler, yaşamın ilk yılında erken cerrahi ile düzeltilmelidir.

Bazılarında meydana gelen diğer belirtiler var, KHH nedeniyle değil ancak tüm hastalarda görülmeyebilir.

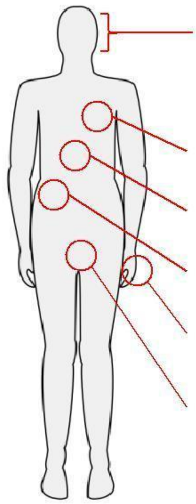

Ergenliğin olmaması birisini nasıl etkileyebilir?

Eşler aynı zamanda ergenlik dönemine girmemek çok stresli ve bazen de travmatik olabilir. Bu KHH'li bazı hastalar için büyük bir problem olabilir. Etki, kişiden kişiye değişmekle birlikte bazı durumlarda ciddi olabilir. Ergenlik çağları ergenlik dönemine giren ergenlik çağındakiler için zor olabilir, ancak ergenlik dönemine girmez ve akran grubu tarafından geride bırakıldığında olası konular ortaya çıkabilir:

- Düşük benlik saygısı, az güven
- Utangaçlık, akranlarla etkileşimde zorluk
- Kaygı, depresyon
- Zayıf vücut imgesi

Anormal bir koku alma duygusu, tat alma duygusu ve yiyecek keyfini önleyebilir . İnsanlar gaz algılayamayabilir (veya gıdalar kötü olduğunda). Bazıları vücut kokusu hakkında gergin olabilir.

Ne yapabilirsin?

Genel olarak, bunlar üstesinden gelinebilir. Psikolojik danışma ve terapi faydalı olabilir.

1. sağlık uzmanınızla konuşun - zor zaman geçirdiğini anlamayabilirler. Danışmanlık ve destek bulmanıza yardımcı olabilirler.

2. Diğer hastalarla bağlantı kurun - çevrimiçi gruplar (Facebook, RareConnect.org) kullanarak ve yüz yüze destek grupları çok yardımcı olabilir. Bunlar, hastaların kendileri için önemli olan konularda konuşabileceği yerlerdir. Diğer hastalar, günlük olarak KHH ile yaşamanın nasıl bir şey olduğunu anlar ve pratik tavsiye ve destek sağlayabilir.

Sağlıklı olmak için ne yapmalısın?

KHH'ye sahip olmak hayatınızı kısaltmamalıdır. Sağlıklı kalabilmek için yapabileceğiniz şeyler vardır:

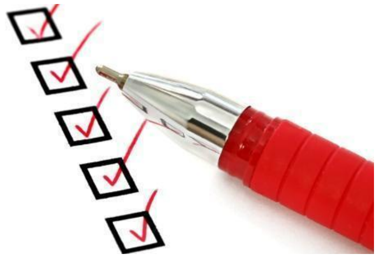

- Devam eden sağlık ve ziyaretler
- Reçete edilen şekliyle tedavinizi yapın
- sağlıklı beslenme
- düzenli egzersiz yapmak
- tütün kullanmayın

KHH için hangi tedaviler mevcuttur?

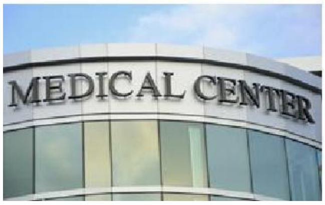

KHH iyileştirilebilir mi?

Şimdilik KHH için herhangi bir tedavi mevcut değil. Doğuştan gelen (genetik) bir rahatsızlığı tedavi etmek çok zordur.
Hipotalamustan GnRH üretimini geri getirebilir miyiz diye araştırma devam ediyor. Bu araştırma halen çok erken bir aşamadadır ancak bunun gelecekte mümkün olacağı umulmaktadır.

KHH için tedaviler var mı?
Erkekler için sakal gelişimi ve kadınlarda meme gelişimi gibi ergenlik belirtilerini geliştirmek için tedaviler mevcuttur. Çoğu durumda doğurganlık geliştirmeye yardımcı olan özel hormon tedavileri.

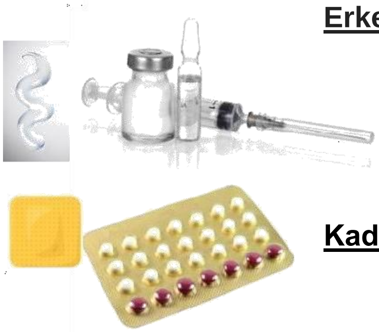

<u>Erkekler:</u> Testosteron (cilde uygulanan jel veya enjeksiyon) ergenlik belirtileri geliştirmek için en yaygın tedavidir. Tedavi, büyümeyi, sesin derinleşmesini, sakal büyümesini, penis büyümesini, cinsel dürtüsülüğü (cinsel tacizu) ve cinsel fonksiyonu tetikler; ancak doğurganlığı artırmaz

<u>Kadınlar</u>: Düşük doz östrojen (yama veya haplar) büyümeyi sağlar ve göğüsler, seksüel gelişim (libido) ve dişil bir figür ile birlikte progesteron ile birlikte düzenli menstrüel periyotlara (kanama) neden olur, ancak doğurganlığa neden olmamasına yardımcı olur.

Tedaviye ilk başladığında, doz ayarlaması aşamalı olarak yapılır. Bu, hızlı sonuçlar bekleyecek bazı insanlar için sinir bozucu olabilir. Bununla birlikte, büyümeyi (kadınlarda meme gelişimi) en üst düzeye çıkarmak önemlidir

Tedaviden sonra KHH'li birkaç kişi iyileşir ve normal hormon seviyeleri üretebilir. Buna bir tersine çevirme denir. Bunun nedenleri anlaşılamamıştır ve kimin böyle bir tersine dönüş yapacağını tahmin edemiyoruz. Bu iyileşme her zaman sürmez.
Dolayısıyla, bir sağlık uzmanı tarafından yakından takip edilmesi önemlidir.

KHH'li yüzde 10 (%10) hastadan 1'inde bir değişiklik var

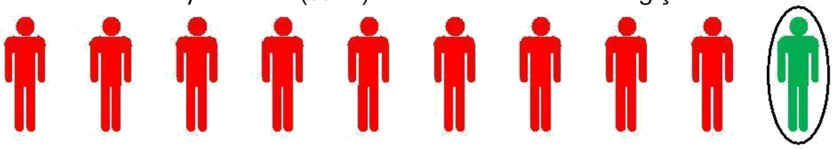

KHH'li bir kişi bereketli olabilir mi?

Evet, KHH tedavi edilebilir bir infertilite şeklidir. KHH'ye sahip olanların çoğunluğu, hepsinden değil, uzmanlaşmış hormon tedavisi ile verimli hale gelebilir

- Özel tedavi haftada birkaç kez hormon enjeksiyonu veya taşınabilir bir GnRH pompası (diyabet için kullanılan pompa gibi) olabilir.
- Doğurganlık tedavisi, deneyimli uzmanların (üreme endokrinologları)
- in vitro fertilizasyon (IVF) gibi bazen (fakat her zaman değil) yardımcı doğurganlığa ihtiyaç duyulmaktadır.

Doğurganlık: Genel olarak, 4 hastanın 3'ü (% 75) başarılı

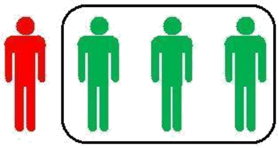

<u>Erkekler:</u> sperm geliştirmek 2 yıla kadar sürebilir
<u>Kadınlar:</u> Bazı durumlar da doğurganlık birkaç ay içinde başarılır

KHH'ye işlem yapılmıyorsa herhangi bir risk var mı?

Testosteron ve östrojen hayat boyu hayati hormon değilken, yokluğu (veya eksikliği) sağlık, cinsel işlev ve yaşam kalitesini ciddi şekilde etkileyebilir

Erkeklerde ve kadınlarda

• Düşük kemik yoğunluğu (osteoporoz) için risk artışı - bu, kemiklerin zayıf olduğu ve kırıkların normalden daha yüksek bir riski olduğu anlamına gelir. KHH tedavi edilmezse, osteoporoz herhangi bir yaşta bir insanı etkileyebilir. KHH olan bazı insanlar osteoporoz için ekstra tedaviye ihtiyaç duyar.

• Cinsel işlevin azalması ve arzunun düşük hormon düzeylerinin bir sonucudur

• Yorgunluk, duygu azalması ve depresyon tedavi dışı hastalar arasında sık görülür

Tedavisi olmayan erkekler de pre-diyabet veya şeker hastalığı gibi metabolik problemleri olma riski yüksektir. Tedavide bulunmakla bu riskler azaltılabilir

***Kilit noktaları*:**

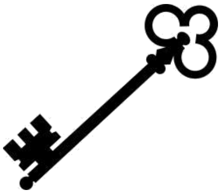

KHH, GnRH eksikliğinin neden olduğu nadir durumdur

• KHH ergenlik ve kısırlığa neden olur

• KHH hayatınızı kısaltmamalıdır

• KHH teşhisi güçtür

• birçok insan geç gençlik yılına veya erken erişkinliğe kadar teşhis edilmez

• Pek çok nadir hastalığın aksine tedaviler mevcuttur

• Yaşam boyu sürecek tedaviye ve doktorunuzla düzenli ziyaretlere ihtiyacınız var

• sizin için en iyi olanı kararlaştırmak için tedavi seçenekleri hakkında doktorunuzla konuşun

Tedaviyi durdurmak sağlığınız ve refahınız üzerinde olumsuz etkilere neden olur

• Çoğu durumda, özel hormon tedavisi ile doğurganlık mümkündür.

• KHH'yi çocuğunuza geçirebilirsiniz, bu nedenle genetik danışmanlık önerilir

• KHH bazı insanlar için psikolojik olarak zor

• Sağlık hizmeti sunucularınızdan ve hasta gruplarınızdan destek
